# Supplementary material for: What are the barriers to the diagnosis and management of chronic respiratory disease in sub-Saharan Africa? A qualitative study with healthcare workers, national and regional policy stakeholders in five countries
Source: BMJ Open. 2022 Jul 29;12(7):e052105. doi: 10.1136/bmjopen-2021-052105 (PMC9345041; doi:10.1136/bmjopen-2021-052105)
Supplement: Supplementary data [file bmjopen-2021-052105supp001.pdf]

Appendix 1: Key informant interview guide for national-level Stakeholders-Kenya

1. Please describe to me your role in this organization. *Probe[How long have you been in this role? Which areas do you work within Kenya? Do you have other work outside the country?]*

2. Overall, how long have you been involved in NCD-related activities? Probe (what exactly drew you to NCD work?)

NCD management is gaining attention across the world. In which ways are you involved in making policies for NCD control in Kenya? *(Probe: is there a specific policy that you are particularly involved in? which one? How are you involved? Policy content, research, advocacy, financing, service provision)*

3.

4. Is chronic respiratory disease one of the NCDs you work with? (If yes – what do you mean by chronic respiratory disease? Please describe the work you do in relation to this? Whose responsibility is CRD in Kenya?

5. What do you think of the health policy on CRD in Kenya? (*Probe:* Is it appropriate? How well is it implemented? What are the key challenges in its implementation? What do you think could be done differently to make implementation better?).

6.

7. Please tell me who your partners are within Kenya. (Explain 'partners' to mean any organizations that you collaborate in any way on CRD work. Probe: national government, county governments, international organizations, CBO, politicians, media, any others?)

8. *If national/county governments are not mentioned in 4- what is your relationship with national/county government? How accessible are you to government decision makers? Are there counties that you partner with? Do you have representation in any of the government committees?*

9. How do you relate with the partners you have mentioned? *(Probe for joint policy development activities, representation in technical committees locally and internationally, advocacy, research, financing, political support).*

10. Among these partners, who do you think is the most powerful in influencing NCD policy development in Kenya? *(Probe: Why do you say so? Is there a partner that you feel should be included in NCD management efforts in Kenya? Why?)*

11. What has worked well in terms of patient care pathways? Why do you say so?

12. What has not worked well so far in terms of patient care pathways? *(Probe: Why do you say so? In your opinion, what could be done differently?)*

13. Over the next 5 years, what are your priorities in NCD control? What about for chronic respiratory disease? What opportunities do you see?

Thank the respondent and end the interview.

Appendix 2: Key informant interviews for policy-makers in national and county governments

**Institution/ Department**	
**Duration**	

1. Please describe to me your role in this department Probe[How long have you been in this role?]
2. Overall, how long have you been involved in NCD-related activities? *(Probe: what exactly drew you to NCD work?)*
3. NCD management is gaining attention across the world. In which ways are you involved in making policies for NCD control in Kenya? (Probe: is there a specific policy that you are particularly involved in? which one? How are you involved?)
4. Is chronic respiratory disease one of the NCDs you work with? (probes - If yes – what do you mean by chronic respiratory disease? Can you describe the work you do in relation to this? If no – can you share more about why not?)
5. Whose responsibility is CRD in Kenya?
6. Please tell me who your key partners are within Kenya. (Explain 'partners' to mean any organizations that you collaborate in any way on CRD work. Probe: national government, county governments, international organizations, CBO, politicians, media, any others?)
7. How do you relate with the partners you have mentioned? (Probe for joint policy development activities, representation in technical committees locally and internationally, advocacy, research, financing, political support).
8. Among these partners, who do you think is most influential CRD policy development in Kenya? Why do you say so? Is there a partner that you feel should be included in CRD management efforts in Kenya? Why?
9. (As appropriate) What is your relationship with national/county government in CRD management? How do you ensure that policy guidelines are adhered to? Do you offer any trainings?

10. What has worked well in terms of patient care pathways? Why do you say so?

11. What has not worked well in terms of patient care pathways so far? Why do you say so?

10. Over the next 5 years, what are your priorities in NCD control? What about for chronic respiratory disease? What opportunities do you see?

Thank the respondent and end the interview.
