# Supplementary material for: What are the barriers to the diagnosis and management of chronic respiratory disease in sub-Saharan Africa? A qualitative study with healthcare workers, national and regional policy stakeholders in five countries
Source: BMJ Open. 2022 Jul 29;12(7):e052105. doi: 10.1136/bmjopen-2021-052105 (PMC9345041; doi:10.1136/bmjopen-2021-052105)
Supplement: Supplementary data [file bmjopen-2021-052105supp002.pdf]

APPENDIX 2_KEY INFORMANT INTERVIEWS WITH STAKEHOLDERS_SUDAN AND TANZANIA

IMPALA Phase1 Topic Guide Two_V2.0_23July2018

Interview Topic Guide for Health Workers (including patient vignettes)

Interview ID NO: ______________ Facilitator Initials: ___________ Note-taker Initials: ________

Type of Participant ________________ Audio file ID _________

Country/Health facility: _____________________ Date of Interview ___________________

Where do you work? ____________________________

What is your job title? ____

How long have you worked as (job title)? __________

Current Disease Priorities within the Health Facility

Can you tell me more about the services that you provide at this health facility?

What are the main priority diseases and illnesses for this facility?

Why are these the main priorities?

Can you tell me about the context of diseases such as Asthma, COPD and TB at this facility?

Readiness for integration of CLD services

Can you tell me more about the services you provide for patients with TB and other lung diseases at this facility?

- Prompt specifically for: Asthma, COPD, occupational lung disease
- How does this link to the community health system? e.g. community health workers, informal health providers etc.
- Please tell me more about how CLDs are diagnosed?
- Please tell me more about how CLDs are managed?

How ready do you feel your health facility is to be able to properly manage and integrate CLDs into routine service/programme delivery?

- ***Case Detection:***
    i. What equipment do you feel is needed to diagnose and manage patients with CLDs?
    ii. What are the challenges with ensuring this equipment if available, functional and used within your facility? What helps?

iii. Do you have a standard case definition for any chronic lung diseases (asthma, COPD etc) to guide you in case detection? (if yes, get some details and request a copy).

iv. Based on your experience in this facility can you comment on how long it takes to detect CLD? (probe for early/late detection, severity of cases when received)

- ***Standardised treatment and effective drug supply, including medical products and technology:***

  i. Are there algorithms for staff in your facility to follow to diagnose and manage patients with CLDs? If yes, who was involved with developing these? Who showed you how to use them? What do you think about these algorithms?

  ii. Are drugs for managing CLDs included in essential drug lists for your facility? Why? Why not? How available are these medicines? How often do you receive medicines? How much do they cost?

- ***Service delivery***

  i. Can you tell me more about the services currently available for patients with TB and other chronic lung diseases (e.g. asthma and COPD) at your facility?

  ii. How do you think the quality of these services could be improved?

  iii. Based on your experiences, what do you think effective CLD care looks like?

  iv. What do you think are the main needs for patients with CLDs?

    v. How prepared is your facility to meet these needs? What changes need to be made to meet these needs?

  b. How do you think services for CLD could be integrated with other services? (e.g. TB/NCDs etc).

- ***Health workforce***

  i. Who do you think should be involved in the management of CLD within your facility? What should their roles and responsibilities be?

  ii. Are there adequate numbers of staff to manage CLDs in your facility? Why? Why not?

  iii. How are staff currently trained to manage CLDs? (pre-service training, in-service training?) How does this vary depending on job role? How could this be improved?

  iv. What supervision structures are in place for health staff involved in the management of CLD within this facility? What works well about supervision? What could be improved?

  v. What is needed to ensure staff are ready to diagnose and manage patients with CLDs?

- ***Reporting, recording of CLD cases and analysing CLD data for action***

  i. What data is currently captured in relation to CLD in existing health information management systems?

  ii. Where is CLD data reported? And how often? (probe for the flow of information)

iii. What analysis is done regarding CLDs? How is this information used?

What are the main barriers to diagnosing and managing patients with CLDs?

- How can these barriers be overcome?

What are the things that help with diagnosis and management currently?

What would make it easier to diagnose and manage patients with CLDs?

What do you think would be the main benefits of integrating the management of CLDs within existing health programmes?

- Which programmes or services would allow for integration?

What do you think would be the main challenges of integrating the management of CLDs within existing health programmes?

- How can these potential threats be avoided?

The following type of patient vignettes will also be used with health workers to assess their current skills for CLD management. It will be made clear to the participants that this is not a test and the results of the study will not be shared with

EXERCISES – Module 9

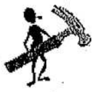

Revise what you have learnt and practise filling in the Asthma Treatment Card and Quarterly Reports.

The following patients have arrived with respiratory symptoms and are suspected cases of asthma. Answer the questions. If it is a case of persistent asthma, record the information on the Asthma Treatment Card.

Patient 1
Man, 60 years old. Retired from work in textile industry.
Clinical signs:
- episodes of coughing with breathlessness and sputum expectoration for the last 3 weeks
- has increased purulence of sputum for the last 5 days
- traces of blood in expectoration once or twice
- temperature has been 38 degrees for the last 3 days

History:
- episodes of coughing in the morning for the last 5 years
- breathlessness after effort for the last year
- temperature has been normal, but 5 kg weight loss over the last year
- no history of allergic disease or tuberculosis
- no known co-morbidity
- Smoking: ex-smoker (smoked 30 cig/day over period of more than 20 years; stopped smoking 3 years ago)

Clinical examination:
- Normal apart from some ronchi and wheezing.

1. What is or what are your suspected diagnoses?……………………………………
2. What do you decide to do? What do you prescribe?
………………………………………………………………………………………………
………………………………………………………………………………………………
………………………………………………………………………………………………

After 8 days, patient improves: coughing, expectoration and breathlessness decrease. Weight is stable. Bacteriological examinations are negative, in particular AFB smear.
Predicted PEF: 536 l/min
PEF measurements are as follows:
PEF before bronchodilator = 280 l/min
PEF after bronchodilator = 290 l/min
However, as the breathlessness has not completely disappeared, the doctor prescribes a short course of prednisone, with salbutamol as needed. After this treatment, the patient comes back and the PEF is measured:
PEF after bronchodilator after 8 days of prednisone = 300 l/min

supervisors/colleagues etc.

3. What is the patient's PEF variability?
………………………………………………………………………………………………
………………………………………………………………………………………………
4. What is the most probable diagnosis?
………………………………………………………………………………………………
5. What is the severity of the disease?
………………………………………………………………………………………………
………………………………………………………………………………………………

If your diagnosis is asthma, complete an Asthma Treatment Card.

6. Teach/Check patient inhaler technique

7. Teach/Check patient ability to self-control attacks
8. How would you manage the patient if he had an acute asthma attack?
9. Please describe how you would respond if the patient told you he was no longer able to carry out his work, due to his breathlessness and he was feeling quite low as a result
    a. What else might you want to consider when managing this patient given his recent low mood?
    b. Are there any other service sectors that you might want to link this patient with?
