# Supplementary material for: What are the barriers to the diagnosis and management of chronic respiratory disease in sub-Saharan Africa? A qualitative study with healthcare workers, national and regional policy stakeholders in five countries
Source: BMJ Open. 2022 Jul 29;12(7):e052105. doi: 10.1136/bmjopen-2021-052105 (PMC9345041; doi:10.1136/bmjopen-2021-052105)
Supplement: Supplementary data [file bmjopen-2021-052105supp003.pdf]

Appendix 3_a: Key informant interview guide for Stakeholders- Uganda

1. Please describe your current role in this organization. Probe [How long have you been in this role? What other roles /positions related to non-communicable diseases do you hold?]

2. Overall, how long have you been involved in NCD-related activities? Probe [how did you come to be involved in NCD work?)

3. NCDs are recognised for being the biggest cause of disease worldwide and are steadily gaining attention in LMICs where they cause avertable premature deaths, what policies are in place for their management in Uganda? [what is the extent of attention received for NCDs in Uganda?]

4. What role do you play in making policies for NCD management in Uganda? [How involved are you in the policy making process? What specific policy stage where you most involved; content, research, advocacy, financing, service provision?]

5. In what ways do you think the policy for NCD management in Uganda is lacking? In which ways is it strong? [what changes would you make to the policy if you had to?]

6. How would you define Chronic lung diseases? In your opinion how big of a problem are CLDs in Uganda?

7. What specific policies are in place for CLD management in Uganda? [Apart from TB policy that is explicit and managed by the TB control program? What guides CLD management at health facilities in Uganda?]

8. Who are some of your partners within Uganda. ['Partners' here refers to any institutions, persons, organizations that you collaborate in any way on CLD work. Probe: national government partners, international organizations and donors, NGOs (international and local), community based organisations, politicians, media, any others? What roles do they mainly play?]

9. How do you relate with the partners you have mentioned? [Probe for joint policy development activities, representation in technical committees locally and internationally, advocacy, research, financing, political support].

10. Among these partners, who do you think are the most powerful in influencing NCD/CLD policy development in Uganda? Who of the partners have a high interest in CLD policy? [Probe: Why do you say so? Who are other partners that you feel should be included in NCD/CLD management efforts in Uganda? Why?]

11. What are the main sources of funding for NCD/CLD management in Uganda?

12. What in your opinion are barriers to implementation of CLD policy at the frontline/health facility level? [what could enhance the implementation of policies at the health facility level?]

13. Who is responsible for ensuring policies are adhered to? [Probes; What role do you have in ensuring that policy guidelines are adhered to? Do you offer any policy related training to frontline workers and implementers of the policies?]

14. What role can patients and their families play in the policy development process for NCD/CLD management in Uganda?

15. Over the next 5 years, what are your priorities in NCD/CLD management in Uganda?

Thank the respondent and end the interview.

Appendix 3_b: Key informant interviews for clinical services directors/facility in-charges at KCCA health facilities- Uganda

1. Please describe your role (s) in this health facility? [Probes: How long have you held this role?]

2. How would you define chronic lung diseases?

3. I am interested in what policies are in place for the management of CLD in this facility and how management of CLDs happens in this facility. Do you have guidelines specific to CLD management? What are your experiences in implementing those guidelines? [Probes; what services for the diagnosis and management of CLD are available? Screening, diagnostics, treatment, follow up.]

4. What was the role your role in development of CLD management policy? What was your role in designing the implementation of the CLD policy? [Probes; if not involved who was? How does the implementation work?]

5. What has worked well so far in treating CLD patients in this facility? [Explain each of the things that have worked well, what has not worked well? Further probes; drugs and medications, patient literacy, patient education and family support?]

6. How can CLD services be improved in this facility?

7. What in your opinion can be done to improve the policies and their implementation? [Do you think you should be involved as a frontline manager in the development of CLD policy? Why? Who else should be involved?]

8. What role can patients play in the development of CLD policy and in the implementation of policy?

9. What in your opinion is the role of involving frontline health workers in developing policies?

10. What is the place of training on a policy at implementation level? [Probes; Were health workers here trained in CLD management? How often does such training occur and who organises the trainings?]

(Respondent may ask questions)
