# Supplementary material for: What are the barriers to the diagnosis and management of chronic respiratory disease in sub-Saharan Africa? A qualitative study with healthcare workers, national and regional policy stakeholders in five countries
Source: BMJ Open. 2022 Jul 29;12(7):e052105. doi: 10.1136/bmjopen-2021-052105 (PMC9345041; doi:10.1136/bmjopen-2021-052105)
Supplement: Supplementary data [file bmjopen-2021-052105supp004.pdf]

Appendix 4_ In-depth interview for healthcare workers: clinical staff e.g. nurse, facility in-charge, CRD specialist

1. Please describe to me your roles in this department (probe: how long have you been in this role? How big is your team? Please describe their roles?)

2. Do you see patients with chronic respiratory diseases? What conditions do you mostly see? How would you know if someone has CRD?. What challenges do you face in diagnosing CRD?

3. What makes people with chronic respiratory symptoms come here? (probe- are they referred here? From where? Who refers them?) Please describe what happens to these patients from the time they come in. (Probe: Screening and diagnostics, treatment, follow up, health education).

4. How does referral work here? Where are patients referred from? Where to? How frequently do you refer patients? After referral, do you maintain contact with the patient? How? Do you get feedback? What happens after discharge?

5. Please tell me what has worked well so far in: diagnosis of g CRD patients

6. Please tell me what has **not** worked well in diagnosis of CRD patients. (For each of the observations, why do you say so?)
7. Please tell me what has worked well so far in: treatment of CRD patients.

8. Please tell me what has **not** worked well in treatment of CRD patients. (For each of the observations, why do you say so?) Probe availability of drugs, timeliness of county governments, affordability of drugs, follow ups).

9. Did you receive any training specific to CRD patients? When was that? Do you have guidelines specific to CRD management? What are your experiences in implementing those guidelines?

10. What are your views of how services for CRD can be improved?

Appendix 4: In-depth interview guide for healthcare workers: Pharmacist

1. Please describe to me your roles in this department (how long have you been in this role? How big is your team? Please describe their roles?)

2. I am interested in understanding the process of treatment for patients who come with chronic respiratory problems. Please describe how these patients get services from the pharmacy. (Probe: prescriptions, drugs access).

3. Please tell me about the preferred drugs for CRD. Which of these do you stock? How do you procure these drugs? What are your experiences of availability of these drugs? Are there any that are covered for by NHIF?

4. Apart from drugs, do you procure any equipment used in the diagnosis of CRD e.g. peak flow meters?

5. Please tell me what has worked well so far in treating CRD patients.

6. Please tell me what has not worked well in treating CRD patients. *(Probe For each of the observations, why do you say so? availability of drugs, timeliness of county governments, affordability of drugs, follow ups).*

7. Did you receive any training on CRD management? (If yes: when?)

8. Do you have guidelines specific to CRD management? What are your experiences in implementing those guidelines?

9. What are your views of how services for CRD can be improved in this county?

Appendix 5: Interview guide for Community Health Extension Officer (CHEW)

1. Please tell me about your role as a CHEW.
2. Is chronic respiratory disease an issue in this CHU?
3. What is your involvement in CRD in your CHU?
4. Please describe how referrals for people with CRD to this facility works (probe: how patients are referred, any documentation from the CHV, records kept by CHEW).
5. How frequently are CRD patients referred? How does it work? What is your involvement?
6. After initial treatment, how do you follow up with patients' treatment? *(Probe: how do you get feedback about patients' condition, supporting follow up)*
7. Did you receive any specific training on CRD management? (If yes, when? Do you have any guidelines for supporting your community work? Have the CHV been trained in CRD case detection?)
8. Do you conduct any outreach services on CRD? How frequently do you do this? What is involved in the outreach?
9. Apart from county government, who are the other partners that you work with? (Probe: international organizations, local organizations)
10. What has worked well so far? Why do you say so?
11. What has not worked well so far? Why do you say so? What could be done differently to improve partnership between the health facility and community?

Appendix 6: In-depth Interview guide for Laboratory staff:

1. Please describe your roles in this department (how long have you been in this role? How big is your team? What does your day to day job involve?).

2. Please describe how patients with cough and/or breathlessness are referred to the lab? Please describe how sputum tests are done here? How frequently do you conduct these tests? Please describe the procedures involved in tests for people with cough and/or breathlessness?

3. In your experience, what are the major outcomes of the tests? How long does it take to get results? How are these results communicated to the patient? After lab, where does the patient go to?

4. Probe: what happens to patients with smear negative results for TB? Are other tests done? Are there patients who keep coming back to this facility? What do you do for them?

5. Please tell me what has worked well so far in testing people with chronic respiratory symptoms.

6. Please tell me what has not worked well in testing CRD patients. (Probe for each of the observations, why do you say so? availability of equipment, reagents).

7. Did you receive any training on CRD management? (If yes: when?)

8. Do you have guidelines specific to CRD management? What are your experiences in implementing those guidelines?

9. What are your views of how services for CRD can be improved in this county?
