# Supplementary material for: What are the barriers to the diagnosis and management of chronic respiratory disease in sub-Saharan Africa? A qualitative study with healthcare workers, national and regional policy stakeholders in five countries
Source: BMJ Open. 2022 Jul 29;12(7):e052105. doi: 10.1136/bmjopen-2021-052105 (PMC9345041; doi:10.1136/bmjopen-2021-052105)
Supplement: Supplementary data [file bmjopen-2021-052105supp006.pdf]

Appendix 6_Topic Guide- Malawi

I really want to begin to understand diagnosis and treatment of chronic respiratory disease in the Malawi health system, and understand factors that have advanced and interfered with diagnosis and treatment

1. Tell me how your **job relates** to chronic respiratory disease (excluding TB). Probe: what is your experience with CRD

2. Tell me about medication supply and availability.
   Probe: what is your perspective on regulation, what is your experience and understanding of common obstacles that occur.
   Access to medication, constraints of:
   Market forces/transparency/donor agenda and funding

3. How do you see Malawian health system changing in recent years addressing chronic health conditions?
   Probe. Specifically CRD, what barriers seen in other countries experiencing.
   - Chronic Care clinic piloted in 2014 in area 25,
   - Package of essential NCD diagnosis and treatment in primary care was trailed in Kasungu District 2012, scale up 2015

4. How do you see clinicians using new Malawi **treatment guidelines** for Chronic respiratory disease diagnosis, and treatment? Spirometry/ Peak flow meter

5. What are some **problem-solving techniques** you use or see clinicians using to provide care to patients seeking medical care who do not have TB?

6. Can you tell me **influencers on actors and action** of NCD and CRD policy? Expand on system thinking in health decision making and policy design?
   Actors/context/process
   Policy communities/networks/venue
   Actors /institution that make decision

7. What do you consider to be the key issues that you see in Malawian health system that create barriers to better treatment of chronic respiratory disease?

   Tell me, have you heard about the Triage Project here in Malawi
   Probe: when did you become involved, what were some key events you saw happen, have you seen any predicted or unpredicted changes or challenges
   Anything else you would like to add?
