# Supplementary material for: What are the barriers to the diagnosis and management of chronic respiratory disease in sub-Saharan Africa? A qualitative study with healthcare workers, national and regional policy stakeholders in five countries
Source: BMJ Open. 2022 Jul 29;12(7):e052105. doi: 10.1136/bmjopen-2021-052105 (PMC9345041; doi:10.1136/bmjopen-2021-052105)
Supplement: Author's manuscript [file bmjopen-2021-052105.draft_revisions.pdf]

BMJ Open

BMJ Open is committed to open peer review. As part of this commitment we make the peer review history of every article we publish publicly available.

When an article is published we post the peer reviewers' comments and the authors' responses online. We also post the versions of the paper that were used during peer review. These are the versions that the peer review comments apply to.

The versions of the paper that follow are the versions that were submitted during the peer review process. They are not the versions of record or the final published versions. They should not be cited or distributed as the published version of this manuscript.

BMJ Open is an open access journal and the full, final, typeset and author-corrected version of record of the manuscript is available on our site with no access controls, subscription charges or pay-per-view fees (http://bmjopen.bmj.com).

If you have any questions on BMJ Open's open peer review process please email info.bmjopen@bmj.com

BMJ Open

Perspectives of healthcare workers, national and regional policy stakeholders on the management of chronic lung disease in five sub-Saharan African countries: tale of a vicious cycle of neglect

Journal:	*BMJ Open*
Manuscript ID	bmjopen-2021-052105
Article Type:	Original research
Date Submitted by the Author:	29-Apr-2021

Complete List of Authors:	MULUPI, STEPHEN; Liverpool School of Tropical Medicine, International Public Health ; KEMRI, Centre for Respiratory Diseases Research<br>Ayakaka, Irene; Liverpool School of Tropical Medicine, International Public Health; Makerere University, Lung Institute<br>Tolhurst, Rachel; Liverpool School of Tropical Medicine, International Public Health<br>Kozak, Nicole; Liverpool School of Tropical Medicine, International Public Health ; REACH Trust Malawi<br>Shayo, Elizabeth; Liverpool School of Tropical Medicine, International Public Health ; National Institute of Medical Research, Tanzania<br>Abdalla, Elhafiz; Epi-Lab<br>Osman, Rashid; Epi-Lab<br>Egere, Uzochukwu; Liverpool School of Tropical Medicine<br>Mpagama, Stellah; 4. Kibong'oto Infectious Diseases Hospital/Kilimanjaro Christian Medical University<br>Chinouya, Martha; Liverpool School of Tropical Medicine, Department of International Public Health<br>Chikaphupha, Kingsley; REACH Trust, Malawi<br>Elsony, Asma; Epi-Lab<br>Meme, Helen; Kenya Medical Research Institute (KEMRI), Centre for Respiratory Diseases Research<br>Oronje, Rose; African Institute for Development Policy (AFIDEP)<br>Ntinginya, Nyanda; National Institute for Medical Research (NIMR)-Mbeya Medical Research Centre ,<br>Obasi, Angela; Liverpool School of Tropical Medicine, International Public Health ; Liverpool University Hospitals NHS Foundation Trust<br>Taegtmeyer, Miriam; Liverpool School of Tropical Medicine, International Public Health ; Liverpool University Hospitals NHS Foundation Trust, Tropical Infectious Diseases Unit

Keywords:	Asthma < THORACIC MEDICINE, Health policy < HEALTH SERVICES ADMINISTRATION & MANAGEMENT, Protocols & guidelines < HEALTH SERVICES ADMINISTRATION & MANAGEMENT, EDUCATION & TRAINING (see Medical Education & Training), PRIMARY CARE, Chronic airways disease < THORACIC MEDICINE

I, the Submitting Author has the right to grant and does grant on behalf of all authors of the Work (as defined in the below author licence), an exclusive licence and/or a non-exclusive licence for contributions from authors who are: i) UK Crown employees; ii) where BMJ has agreed a CC-BY licence shall apply, and/or iii) in accordance with the terms applicable for US Federal Government officers or employees acting as part of their official duties; on a worldwide, perpetual, irrevocable, royalty-free basis to BMJ Publishing Group Ltd ("BMJ") its licensees and where the relevant Journal is co-owned by BMJ to the co-owners of the Journal, to publish the Work in this journal and any other BMJ products and to exploit all rights, as set out in our licence.

The Submitting Author accepts and understands that any supply made under these terms is made by BMJ to the Submitting Author unless you are acting as an employee on behalf of your employer or a postgraduate student of an affiliated institution which is paying any applicable article publishing charge ("APC") for Open Access articles. Where the Submitting Author wishes to make the Work available on an Open Access basis (and intends to pay the relevant APC), the terms of reuse of such Open Access shall be governed by a Creative Commons licence – details of these licences and which Creative Commons licence will apply to this Work are set out in our licence referred to above.

Other than as permitted in any relevant BMJ Author's Self Archiving Policies, I confirm this Work has not been accepted for publication elsewhere, is not being considered for publication elsewhere and does not duplicate material already published. I confirm all authors consent to publication of this Work and authorise the granting of this licence.

For peer review only

Perspectives of healthcare workers, national and regional policy stakeholders on the management of chronic lung disease in five sub-Saharan African countries: tale of a vicious cycle of neglect

Authors: Stephen Mulupi*[1,2], Irene Ayakaka*[1,3], Rachel Tolhurst[1], Nicole Kozak[1,4], Elizabeth Shayo[1,5], Elhafiz Abdalla[6], Rashid Osman[6], Uzochukwu Egere[1,] Stellah Mpagama[7], Martha J. Chinouya[1], Kingsley Chikaphupha[4] , Asma El Sony[6], Hellen Meme[2] , Rose Oronje[8], Nyanda Elias Ntinginya[5], Angela I. Obasi[1,9], Miriam Taegtmeyer[1,10]

*Joint first authors

Corresponding author: Stephen Mulupi

E-mail: stephen.mulupi@lstmed.ac.uk

Mobile phone number +254720640330

Affiliations:

1. Liverpool School of Tropical Medicine, Department of International Public Health, Liverpool, UK
2. Kenya Medical Research Institute, Centre for Respiratory Diseases Research (KEMRI-CRDR)
3. Makerere University Lung institute, (MLI)
4. REACH Trust, Malawi
5. National Institute for Medical Research, Tanzania
6. EpiLab, Sudan
7. Kibong'oto Infectious Disease Hospital, Tanzania
8. African Institute for Development Policy (AFIDEP)
9. AXESS Sexual Health, Liverpool University Hospitals NHS Foundation Trust, Liverpool, UK
10. Tropical Infectious Diseases Unit, Liverpool University Hospitals NHS Foundation Trust, Liverpool, UK

Contact information for authors:

1. Stephen Mulupi: stephen.mulupi@lstmed.ac.uk
2. Irene Ayakaka: irene.ayakaka@lstmed.ac.uk
3. Rachel Tolhurst: rachel.tolhurst@lstmed.ac.uk
4. Nicole Kozak: molemoe@gmail.com
5. Elizabeth Shayo: bshayo@yahoo.com
6. Elhafiz Abdalla: hafizhussein@gmail.com
7. Rashid Osman: rashildo@hotmail.com
8. Uzochukwu Egere: uzochukwu.egere@lstmed.ac.uk
9. Stellah Mpagama: sempagama@yahoo.com
10. Martha J. Chinouya: martha.chinouya@lstmed.ac.uk
11. Kingsley Chikaphupha: kchikaphupha@gmail.com.
12. Asma El Sony: asmaelsony@gmail.com
13. Hellen Meme: helenmeme@gmail.com
14. Rose Oronje: rose.oronje@afidep.org
15. Nyanda Elias Ntinginya: nelias@nimr-mmrc.org
16. Angela I. Obasi: Angela.Obasi@lstmed.ac.uk
17. Miriam Taegtmeyer: miriam.taegtmeyer@lstmed.ac.uk

For peer review only - http://bmjopen.bmj.com/site/about/guidelines.xhtml

On behalf of The NIHR International Multidisciplinary Programme to Address Lung Health and TB in Africa (IMPALA) Consortium: Emmanuel Addo-Yobo, Brian Allwood, Hastings Banda, Imelda Bates, Amsalu Binegdie, Adegoke Falade, Jahangir Khan, Maia Lesosky, Bertrand Mbatchou, Kevin Mortimer, Beatrice Mutayoba, Louis Niessen, Jamie Rylance, S Bertel Squire, William Worodria, Heather Zar, Eliya Zulu, Lindsay Zurba

ABSTRACT

Objectives To explore the perspectives of policy stakeholders on the diagnosis and management of chronic lung diseases (CLDs) in Kenya, Malawi, Sudan, Tanzania, and Uganda.

Settings Primary, secondary, and tertiary health facilities, government agencies, and civil society organisations in 5 sub-Saharan African countries.

Participants We purposively selected 60 national, and district level policy stakeholders, and 49 healthcare workers and conducted key-informant interviews (KII) and in-depth interviews (IDI) respectively, between 2018 and 2019.

Findings

We identified intersecting vicious cycles of neglect of CLDs at strategic policy and healthcare facility levels. Lack of reliable data on CLDs burden, due to weak information systems and diagnostic capacity, negatively affected CLDs inclusion in policy, this in turn was reflected by low budgetary allocations for diagnostic equipment, training, and medicines. At the healthcare facility level, inadequate budgetary allocations constrained diagnostic capacity, quality of service delivery and collection of appropriate data, compounding the lack of routine data on burden of disease.

Conclusion

Health systems are ill-equipped to respond to the rising burden of CLD, an issue that has been brought into sharp focus as countries plan for post-COVID-19 lung diseases. CLDs are under-diagnosed, under-reported and underfunded leading to a vicious cycle of invisibility and neglect. Appropriate diagnosis and management of CLDs require health systems strengthening, particularly at the primary healthcare level.

Strengths and limitations

Strengths

- The five studies were conducted independently by the country study teams, allowing for contextual country flexibilities.
- The KII and IDI are appropriate in investigating and triangulating perspectives of study participants, enhancing rigour in the data collection.

Limitations

- Contextual differences in study sites e.g. number and levels of hospitals and rural/ urban variations may limit comparisons in healthcare experiences.
- Exclusion of patients' perspectives in demanding and accessing health services for CLDs.

For peer review only - http://bmjopen.bmj.com/site/about/guidelines.xhtml

For peer review only

For peer review only - http://bmjopen.bmj.com/site/about/guidelines.xhtml

1. Introduction

Non-communicable lung diseases (NCLD) account for approximately 4 million deaths per year world-wide, [1, 2] with the burden of NCLD morbidity and mortality rising steeply in low- and middle-income countries (LMICs).[3, 4] The high prevalence of NCLD in sub-Saharan Africa (SSA) is driven in part by high rates of recurrent childhood respiratory infections and pulmonary TB which are important precursors of NCLD. [5] [6-8] A hospital-based study in Tanzania among post-TB patients estimated 74% prevalence of abnormal lung function. [9] Other common risk factors include exposure to tobacco smoke, indoor pollution from biomass fuels and occupational exposures in the mining industry, sugar, and tobacco plantations. [10, 11] In Uganda, the age-adjusted prevalence for any chronic respiratory condition in both rural and urban settings is 20% [12] and in Malawi over 40% of adults randomly sampled in an urban population had abnormal lung function. [13]

The management of NCLD in SSA is undermined by weak health systems characterised by lack of appropriate medical equipment and low diagnostic capacity. [14, 15] Healthcare workers trained in the management of NCLD are scarce and medicines are either unavailable or unaffordable by many patients.[16, 17] Existing lung health pathways in SSA focus on TB with smear negative patients repeatedly visiting health facilities or being referred from one facility to another seeking definitive diagnosis. A wide range of policy design, implementation and service delivery gaps undermine NCLD care pathways. We present policymaker and healthcare worker perspectives on NCLD policies and services from in-depth and key informant interviews conducted in five countries in SSA. The five linked studies formed baseline assessments for country-level research within the National Institute for Health Research (NIHR), International Multidisciplinary Programme to Address Lung Health and TB in Africa (IMPALA[1]).

Methods

In-depth qualitative interviews were conducted in Kenya, Malawi, Sudan, Tanzania, and Uganda 2018-19. Ministry of Health officials and other healthcare policy stakeholders were purposively sampled for their roles and experiences in policy design and implementation alongside health workers experienced in the management of NCLD (table 1). Public healthcare facilities were selected to include both urban and rural settings and different facility levels (table 2). All served populations with high prevalence rates of TB and other lung conditions.

Table 1: The distribution of participants across the five case study countries.

**Informants**	**Kenya**	**Malawi**	**Sudan**	**Tanzania**	**Uganda**
National, regional and district level policy stakeholders	15	13	14	13	5
Health care workers	14	5*	14	10	6
**Total**	**29**	**18**	**28**	**23**	**11**

***Senior clinicians**

[1] https://www.lstmed.ac.uk/impala

For peer review only - http://bmjopen.bmj.com/site/about/guidelines.xhtml

Table 2: Distribution of healthcare facilities in the five case study countries

Public health system level	Kenya	Malawi	Sudan	Tanzania	Uganda	Total
Primary healthcare facilities (health centre and dispensary level)	3	1	2	8	6	20
District level hospitals	1	1	8	1	0	11
National/regional referral hospitals	0	2	0	1	2	5
**Total number of facilities**	**4**	**4**	**10**	**10**	**8**	**36**

Role of the funding source

This research was funded by the (NIHR IMPALA, grant reference 16/136/35) using UK aid from the UK Government to support global health research. The views expressed in this publication are those of the author(s) and not necessarily those of the NIHR or the UK Department of Health and Social Care.

Data collection

Key informant interviews (KIIs) with policy stakeholders explored opinions about prioritization of NCLD; (un)availability and operationalization of NCLD policies; systemic factors enhancing or impeding provision of healthcare services for NCLD; and opinions on how systems could be improved. In-depth interviews with healthcare workers, explored their experiences in diagnosing and managing NCLD; availability of diagnostic equipment and medicines; experience of training; and their perceptions of what has worked well or not, in management of NCLD. Interview guides were developed broadly aligning with the health systems' building blocks. [18] Interviews were conducted May 2018 to March 2019, by experienced qualitative researchers in English, or the local languages as appropriate, and digitally recorded with consent. Recordings were transcribed verbatim and translated for analysis.

Intercountry analysis.

Data collection was preceded by a common training in policy (2018) and followed by joint analysis meetings that discussed similarities and differences between country-specific study findings. Respective country teams re-read transcripts to confirm accuracy and identify emerging themes. Initial codebooks were developed from the broad interview guide topics, then updated inductively as novel codes emerged. A final codebook was discussed, further refined, and applied to the data, using NVivo v11.0 (QSR International 1999). An exploration of emerging patterns led to the identification of final themes with selected quotes used to illustrate specific findings.

Ethics

The Liverpool School of Tropical Medicine Ethics Committee approved these studies separately (Kenya: protocol 18-054; Uganda: protocol 18-037; Malawi: protocol M1803; Tanzania and Sudan; protocol 18-043). Additionally, each approval was by in-country committees[2]. All participants gave written, informed consent.

[2] Kenya Medical Research Institute (KEMRI/SERU/CRDR/037/3717); Malawi National Health Science Research committee (Protocol # 18/04/2021); Sudan: Ref 44/T/Kh/1; Tanzania: Medical Research Coordinating

For peer review only - http://bmjopen.bmj.com/site/about/guidelines.xhtml

Findings

We used the World Health Organization (WHO) health systems building blocks,[18] to frame our exploration at strategic and service delivery levels. A picture emerged of neglect of NCLD at each level of the system and for each building block with consequences across the whole system.

Strategic level

Variable availability and awareness of policy

NCLD policy availability varied between countries. Kenya and Tanzania have fully developed and adopted policy strategies for lung health[19, 20] and the national and district stakeholders were aware of this; however, few health care workers were aware of the policies or where to find them. There was no stand-alone policy for NCLD in Uganda and in Malawi; NCLD was embedded within NCD policy, which was in draft, with limited awareness among all levels of participants. In Sudan, there was no specific policy for NCLD.

> *"I do not know whether there is an existing policy for TB and NCLD. There is a general strategy; the national health policy that generally addresses chronic diseases." (KII-F, Sudan Federal-MoH official)*

Lack of NCLD data

In all five contexts, policy stakeholders associated the limited data on burden of disease with low visibility and low domestic budget allocations for NCLD as shown by this typical quote:

> *"For COPD we don't have data, we don't know its prevalence, we don't know its incidence, we don't know its mortality" (IDI national informant, Tanzania).*

None of the study countries had comprehensive population surveys on NCLD, but Sudan, Kenya and Uganda reported research evidence about asthma, COPD and the growing burden of NCLD. In Malawi, participants noted that NCLD was not included in the recent nationwide burden of disease study and went further to highlight the lack of data on cost-effectiveness.

> *" So [NCLDs] never feature in the cost effectiveness analyses that allow for their prioritization, benchmarked against the other priorities where there is evidence….for chronic respiratory disease we have to recognize that the evidence base for the country is very tiny. So being able to advocate beyond, the NCD Department into the Treasury, into the Health Sector Strategic Plan, is multiple steps away" (Malawi-KI 18, researcher).*

Neither was routine data able to fill the evidence gaps due to a lack of appropriate data collection tools and shortages of health information and records officers. Participants in all the five countries emphasized the need to address these gaps in developing investment cases for NCLD at national level.

> "*You perform a study and show them the results. How many are affected? How many are disabled? How many lose their jobs? How many houses lose support? If you provide such work, you might be able to convince the (national treasury) officials*" *(KII-S5, Gezira State, Sudan MoH official).*

Lack of donor prioritisation decreases budgetary allocation

The health system financing arrangements in all the study countries were reported as being heavily dependent on external partners. NCLD was perceived to be given low priority by donors because of their generally non-infectious nature and the perception that they are less fatal than infectious diseases such as malaria, HIV/AIDS and TB, and maternal and child health conditions. Even within the NCD departments in the study countries, efforts and resources are majorly directed towards cancer, diabetes, hypertension and other cardiovascular diseases.

Committee of the National Institute for Medical Research, (NIMR/HQ/R.8a/V.IX/2922) and Uganda (TASO) IRB:TASOREC /030/18-UG-REC-009 and the Uganda National Council for Science and Technology HS232ES).

For peer review only - http://bmjopen.bmj.com/site/about/guidelines.xhtml

Current priorities

Positive steps in data collection and policy development were also described. In response to a global spotlight on NCDs and recent increased donor interest, the Kenyan and Tanzanian governments plan to close the evidence gap by capturing NCLD data in routine health facility tools, and subsequent nationwide surveys like the Demographic and Health Surveys.

> *"We are refining the tools to better capture the NCDs and have specific NCD registers like in the general outpatient settings (KII, Kenya-MOH, national level)*

At the time of data collection, Kenya was in the process of ratifying the Protocol to eliminate illicit trade in tobacco products, and Uganda had banned tobacco smoking in public, tobacco advertising, promotion and sponsorship, and limited sales. This suggests a heightened prioritization of an important risk-factor of NCLD on the policy agenda, with potential for downstream policy interventions.

Service level

Key service level barriers arising from policy failures included challenges in accessing NCLD diagnostics and medicines, limited reporting and low confidence and skill in diagnosis and management. There was a disconnect between the perceptions of policy stakeholders, which reflected what was available on paper, and the front-line health workers, who reported feeling ill-equipped to attend patients in practice.

Lack of diagnostic equipment

Participants universally agreed that the entry point to NCLD diagnosis in their TB-endemic contexts required the exclusion of active TB disease in chronic cough. Diagnostic capacity was defined as a combination of sputum screening, with the availability of radiology, lung function testing, skilled staff and diagnostic and treatment algorithms for the common NCLDs (asthma, post TB lung disease and COPD). While each study country had chest clinics at tertiary hospitals with some level of equipment and expertise, only TB screening had been systematically decentralised at lower levels.

Challenges were described in each aspect of diagnosis, with patients often paying out-of-pocket costs. All countries reported delays in sputum screening. In some instances, these delays extended to more than a week and were associated with additional costs to patients and subsequent loss to follow up.

Finally, the widespread lack of (or dysfunctional) x-ray equipment and lack of qualified personnel reported in lower level public facilities in Kenya, Malawi, Uganda, and Tanzania, meant that patients had to seek chest x-rays (and CT scans) from private healthcare providers, whose costs were unaffordable to most. The lack of peak flow meters and spirometry machines meant that patients requiring lung function testing were referred to the national teaching and referral hospitals.

> *"Yeah there was a time, we also [provided spirometry] but …our machine broke down … when we see they are needing that spirometry we send them to Kenyatta National Hospital (Healthcare Worker, Kenya).*

In Tanzania, participants noted that despite the recommendation to have peak flow meters at the primary healthcare level, none were available and not a single facility (including the referral hospital) offered spirometry. In Kenya, Malawi, Sudan, and Uganda, the use of spirometry was reported to be limited to tertiary hospitals with international research collaborations, where training and equipment are maintained through grants from external partners. Although some policy level stakeholders thought otherwise, health workers at the lower level hospitals in all countries expressed dissatisfaction with the lack of peak flow meters and spirometers. In Malawi, there were conflicting reports on the distribution of spirometry services across facilities. Whilst policymakers claimed that all tertiary hospitals had spirometers, healthcare workers reported that spirometers were only available at one teaching hospital as part of a research project.

Lack of NCLD training and guideline dissemination

For peer review only - http://bmjopen.bmj.com/site/about/guidelines.xhtml

According to majority of key informants in Kenya, Uganda, and Tanzania most healthcare workers had not received training on the latest diagnostic procedures for NCLD, including the use of spirometry:

> *"Honestly, we have not received any training for NCLD; we used to go for TB trainings only" (IDI, Healthcare worker, Tanzania)*

Malawian participants described a successful pilot decentralising NCLD training, but this was yet to be scaled-up nationally. Participants in all contexts emphasized the need improve in-service training countrywide. In-service training opportunities were *ad hoc* and often *'erratic'* (Sudan); mostly supported by specific programmes, including research projects (e.g. Uganda), non-governmental organizations and pharmaceutical companies promoting specific products; and characterised by low coverage of necessary staff.

> *"…we started a program with some collaborative partners, … they bring health workers from some districts and we come and do modules to understand for example what asthma is, how its diagnosed what is the best treatment, because in [medical] school frankly, people don't learn so much for the benefit of the wider community (MOH consultant physician Uganda)."*

Most of the available training focused on TB. In Tanzania this was to the exclusion NCLD. In Kenya, NCLD were added on to TB training. Healthcare workers in Kenya reported difficulties in accessing professional training, within the devolved government setup. Lack of financial sponsorship from government, and a requirement that they resign if they applied for fulltime study imposed steep opportunity costs.

Standard processes of communicating guidelines from national policy level to service delivery levels were felt to be inadequate; for example, healthcare workers in Kenya described learning about asthma management guidelines incidentally, during internet use or informal discussions with colleagues. There was limited awareness of treatment guideline updates with many clinicians still prescribing available oral salbutamol instead of inhalers, despite lack of evidence for efficacy. Likewise, in Tanzania, although the NCLD guidelines were available on the Ministry of Health website and copies were seen at the sites, participants seemed unaware of their content.

Limited availability of drugs and lack of confidence in management

Restrictive policy guidelines and user-fees imposed barriers to accessing corticosteroid inhalers. For example, beclomethasone is classified as a central facility level medicine in Malawi, that required international procurement protocols prone to delays of 4-7 months. In Tanzania only bronchodilators, injectable and oral steroids for acute asthma are allowed at lower levels of service provision. Similarly, in Uganda some medicines were restricted at the primary level. In contrast in Kenya bronchodilators and corticosteroids have been included in pharmacy order forms for primary health care facilities where they are provided free, implying shifts in practice to enhance access. In Sudan, asthma medications including inhalers and corticosteroids are supposed to be provided free of charge especially for patients in acute attacks. However, these are often not available in emergency rooms at both district and national levels, and when available, they are not always free of charge.

> *"Some of the services for asthma might be provided for free in the emergency room, but the majority of it is not provided for free although it should [be]" (KII-S4, Gezira State MoH official).*

Additionally, respondents from all five countries reported frequent drug stockouts even of the few NCLD treatments included on essential medicines lists. At the time of data collection, the Kenya Medical Supplies Authority had stopped supplies to counties with outstanding bills. At primary care level in Tanzania and Uganda drugs other than oral salbutamol were unavailable.

Frontline clinicians at primary and district level felt ill-equipped to meet the complexity of cases. Healthcare workers explained that they lacked confidence in managing chronic NCLD, although acute asthma attacks would be treated (nebulisers, oral aminophylline and adrenalin injections were all mentioned).

For peer review only - http://bmjopen.bmj.com/site/about/guidelines.xhtml

> *"we end up using treatments we are not supposed to (for example oral salbutamol) because that is what is available (Uganda - Clinical officer HC III).*

Key informants in Kenya and Uganda, mentioned measures to enhance treatment capacity through review of the curricula for in-service training of clinical and medical officers, and for Kenya, the development of 'module 13' for community health volunteers, on identifying and managing asthma.

> *"we are working closely with KMTC itself, the Kenya Clinical Officers Council and the Nursing Council of Kenya to give NCD's more prominence in the pre-service training (KII_MOH, Kenya)*

Most healthcare workers, however referred chronic asthma and other undiagnosed NCLD conditions to higher level facilities, but even at this level limited capacity for management was described. All five countries reported very few pulmonologists. Malawi for example, had only one specialist respiratory clinician, who often did not receive referrals because of low awareness among healthcare workers.

Poor reporting and reduced health care worker awareness

Low healthcare workers' diagnostic capacities implied that subsequent data inputs into the health information system may not provide reliable evidence of the NCLD disease burden. Furthermore, the appropriate coding of diseases overall, is undermined by shortage of health information officers, and essential reporting tools.

> *"…then you find most of our medical personnel record "chronic cough" which is not a diagnosis … we are championing for this cadre of staff (health records information officers) because they are very critical otherwise, we would continue making wrong decisions based on poor data (KII-Non-State Actor, Kenya)*

This poor reporting was felt in turn to reduce awareness of NCLD among health care workers of the need for clinical follow-up and of the overall scale of the problem.

DISCUSSION

Based on data from five Sub Saharan African countries (Kenya, Malawi Tanzania, Sudan, and Uganda) we identified how neglect of NCLD at strategic and service levels form two intersecting vicious cycles as illustrated in Figure 1.

Figure 1: Chronic Lung Disease in Sub-Saharan Africa - Intersecting vicious cycles of neglect

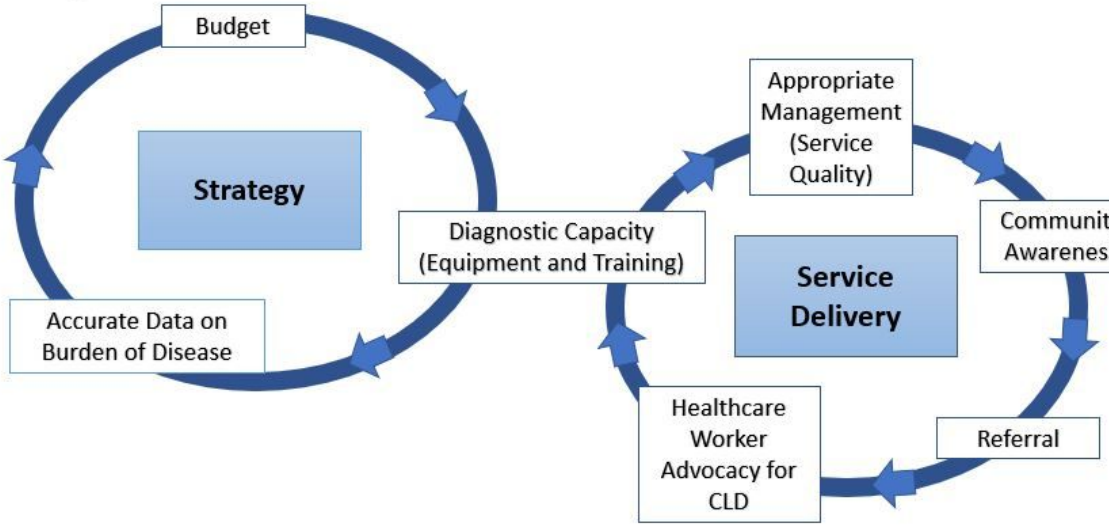

Whilst specific weaknesses varied by context, common cycles were discernible. At strategic policy level, low diagnostic capacity, weak recording and reporting systems limited the availability of reliable data on the burden of lung diseases, negatively affecting inclusion in policy, and in turn budgetary

For peer review only - http://bmjopen.bmj.com/site/about/guidelines.xhtml

allocations for diagnostic equipment, training, and medicines. At the service level, lack of budgetary allocations for equipment and training of staff constrained diagnostic capacity, which, along with limited availability of appropriate medicines reduced service delivery quality and collection of appropriate data within healthcare facilities. Providers felt frustrated at their lack of capacity to diagnose and treat these conditions; they were aware that that their own skills to support patients with NCLD over time were limited.

To strengthen health systems in these countries and others in similar contexts, it will be important to break these vicious cycles,[21] although the most strategic entry points may vary by country. For example, variations in the development of policy strategies specific to lung health suggest policy change is more important in some contexts with NCD policy and TB control policy both providing possible avenues of entry[22]. Improvements in diagnostic capacity, along with improving accuracy of reporting, may create impetus for policy commitments and corresponding budgetary allocations, required to improve availability of medicines. Concurrent improvements in systems 'software' such as communication, advocacy, and accountability are required to realise policy changes in practice.[23, 24] For example, strategic communication of local data and accountability for responsiveness to this is needed, not only on burden of disease and service use but also on stockouts, broken equipment, or vacant posts.[25] Ultimately a team approach (including managers, clinical providers, health information officers and community actors) will be needed to ensure a coherent approach and feed-back loops. Effective communication of guidelines and protocols needs to be accompanied by operational plans to enact them. For example, current GINA guidelines for asthma control advocate use of inhaled corticosteroid-formoterol for the management of mild asthma and yet many of the study countries restrict availability at the primary health care facilities most likely to see cases.[26]

It is likely to be too simplistic to assume that changes at one point in the cycle will lead to the desired changes in a linear way throughout the cycle, since challenges in a complex system are likely to be multi-causal. [27] Evidence is only one, often small, element in policymaking. [28] In turn, written policy may not be translated into budgetary allocations or materialise as equipment and medicine availability due to intense budgetary competition; the role of donor priorities; 'leakage' of resources through the system; procurement and maintenance challenges, amongst others. Decentralisation offers opportunities for local (burden of disease) data and demands to influence budgetary allocations, although it may also mean greater political struggles over priorities [29, 30] and increased fragmentation across the national health system. In many contexts, inadequate human resources and high staff attrition rates in rural areas provides a significant challenge to maintaining trained providers. Interventions in one part of the system may have unintended consequences in another; [24] where provider workloads are high, improved diagnostic capacity for certain diseases may deprioritise others, including by increasing service utilisation. Efforts will therefore be needed to track changes throughout the system, and beyond NCLD. Finally, management of NCLD, like other chronic conditions, requires continuity of care, effective linkage between health facilities and community systems, and patient empowerment. [11, 31] Significant communication is therefore required by providers, including community health workers, to increase awareness of NCLD and support patients with transition to self-management of their conditions.

Health systems in LMICs are currently designed to respond to acute, mostly communicable, illnesses usually through disease-specific vertical programmes. Despite some adaptation to treat people living with HIV, these systems often respond poorly to chronic conditions such as NCLD, that require sustained supply of medicines, continuity of care, and strong linkages between healthcare facilities and community health systems, with earmarked funding. Rather than adding a new vertical programme, or a new set of diseases to existing programmes, we advocate for a system-wide approach to a range of chronic diseases.

Limitations

The five studies were conducted independently, by different research teams with variations in the topic guides allowing for context, a larger number of district hospitals included in Sudan and a focus on urban sites in Kenya, Malawi and Uganda with the inclusion of rural sites in Tanzania, and Sudan. While these independently conducted studies established similar findings in diagnosis and management of NCLD,

For peer review only - http://bmjopen.bmj.com/site/about/guidelines.xhtml

suggesting important systemic challenges across the SSA countries, the findings should nevertheless be interpreted with caution, as environmental exposures and service delivery contexts may differ with the distribution of healthcare facilities and professionals relatively higher in urban settings. We did not include NCLD patients, community health workers and at-risk community members' perspectives in our study but focused on the overall, issues that affect the planning and care of the patients.

Recommendations and conclusions
The COVID-19 pandemic has brought into sharp focus the urgent need to reform and strengthen healthcare systems to effectively respond to people with chronic health conditions, including NCLD, an important risk factor to COVID-19 severity. In the five SSA countries studied, interlinked gaps at the policy strategic level, and healthcare delivery levels, undermine appropriate provision of services for people with NCLD. Lack of diagnostic capacity is a major link between the vicious cycles, influencing both the ability to manage cases within services and lack of accurate data on NCLD to inform policy responses and resource allocation. Improvements are required across all key elements of the service delivery systems, including pre- and in-service training for diagnosis and management, guideline dissemination, diagnostic equipment, recording and reporting. Additionally, there is urgent need to enhance reliable, affordable access to drugs, particularly the inhaled corticosteroid-formoterol inhaler in the early stages of asthma management in children, adolescents, and adults. Enhancing collection of population-level data to ascertain the true burden of disease may be an important entry point to drive policy change ensuring that people living with NCLD are not 'left behind' in the development of Universal Healthcare.

Author contributions

1. Stephen Mulupi- literature search, data collection, analysis, interpretation, writing
2. Irene Ayakaka: literature search, data collection & analysis, interpretation, writing
3. Nicole Kozak: literature search data collection & analysis, writing
4. Elizabeth Shayo: Data collection & analysis, writing
5. Hafiz Hussein: Data collection & analysis, writing
6. Rashid Osman: Data collection & analysis, writing
7. Stella Mpagama: Data collection & analysis, writing
8. Rachel Tolhurst: Study design, data analysis, interpretation, writing
9. Uzochukwu Egere: Study design, interpretation, writing
10. Martha Chinouya: Study design, interpretation, writing
11. Kingsley Chikaphupha: data analysis, interpretation, writing
12. Asma El Sony: Interpretation, writing
13. Hellen Meme: Interpretation, writing
14. Rose Oronje: Interpretation, writing
15. Nyanda Elias Ntinginya: Interpretation, writing
16. Angela Obasi: Study design, Interpretation, writing
17. Miriam Taegtmeyer: Study design, data analysis, interpretation, writing

Declaration of competing interests
None declared

Patient and Public involvement:

Patients or the public WERE involved in the design, or conduct, or reporting, or dissemination plans of our research

For peer review only - http://bmjopen.bmj.com/site/about/guidelines.xhtml

For peer review only

References

1. World Health Organization. Non communicable diseases, Fact sheet. In: WHO, editor. 2017.
2. Soriano JB, Kendrick PJ, Paulson KR, Gupta V, Abrams EM, Adedoyin RA, et al. Prevalence and attributable health burden of chronic respiratory diseases, 1990–2017: a systematic analysis for the Global Burden of Disease Study 2017. The Lancet Respiratory Medicine. 2020;8(6):585-96
3. Marais BJ, Loennroth K, Lawn SD, Migliori GB, Mwaba P, Glaziou P, et al. Tuberculosis comorbidity with communicable and non-communicable diseases: integrating health services and control efforts. The Lancet infectious diseases. 2013;13(5):436-48
4. Samb B, Desai N, Nishtar S, Mendis S, Bekedam H, Wright A, et al. Prevention and management of chronic disease: a litmus test for health-systems strengthening in low-income and middle-income countries. The Lancet. 2010;376(9754):1785-97
5. World Health Organization. Global Tuberculosis Report, 2019. Geneva: World Health Organization; 2019.
6. Byrne AL, Marais BJ, Mitnick CD, Lecca L, Marks GB. Tuberculosis and chronic respiratory disease: a systematic review. International Journal of Infectious Diseases. 2015;32:138-46
7. Amaral AF, Coton S, Kato B, Tan WC, Studnicka M, Janson C, et al. Tuberculosis associates with both airflow obstruction and low lung function: BOLD results. European Respiratory Journal. 2015;46(4):1104-12
8. Menezes AMB, Hallal PC, Perez-Padilla R, Jardim J, Muino A, Lopez M, et al. Tuberculosis and airflow obstruction: evidence from the PLATINO study in Latin America. European Respiratory Journal. 2007;30(6):1180-5
9. Manji M, Shayo G, Mamuya S, Mpembeni R, Jusabani A, Mugusi F. Lung functions among patients with pulmonary tuberculosis in Dar es Salaam – a cross-sectional study. BMC Pulmonary Medicine. 2016;16(1):58
10. Banda HT, Thomson R, Mortimer K, Bello GA, Mbera GB, Malmborg R, et al. Community prevalence of chronic respiratory symptoms in rural Malawi: Implications for policy. PloS one. 2017;12(12):e0188437
11. Bischoff A, Ekoe T, Perone N, Slama S, Loutan L. Chronic disease management in Sub-Saharan Africa: whose business is it? International Journal of Environmental Research and Public Health. 2009;6(8):2258-70
12. Siddharthan T, Grigsby M, Morgan B, Kalyesubula R, Wise RA, Kirenga B, et al. Prevalence of chronic respiratory disease in urban and rural Uganda. World Health Organization Bulletin of the World Health Organization. 2019;97(5):318-27
13. Meghji J, Nadeau G, Davis KJ, Wang D, Nyirenda MJ, Gordon SB, et al. Noncommunicable lung disease in sub-Saharan Africa. A community-based cross-sectional study of adults in urban Malawi. American journal of respiratory and critical care medicine. 2016;194(1):67-76
14. van Gemert F, van der Molen T, Jones R, Chavannes N. The impact of asthma and COPD in sub-Saharan Africa. Primary Care Respiratory Journal. 2011;20(3):240
15. Arokiasamy P, Kowal P, Capistrant BD, Gildner TE, Thiele E, Biritwum RB, et al. Chronic noncommunicable diseases in 6 low-and middle-income countries: findings from wave 1 of the World Health Organization's study on global Ageing and adult health (SAGE). American journal of epidemiology. 2017;185(6):414-28
16. Kibirige D, Sanya RE, Nantanda R, Worodria W, Kirenga B. Availability and affordability of medicines and diagnostic tests recommended for management of asthma and chronic obstructive pulmonary disease in sub-Saharan Africa: a systematic review. Allergy, Asthma & Clinical Immunology. 2019;15(1):14
17. Kibirige D, Kampiire L, Atuhe D, Mwebaze R, Katagira W, Muttamba W, et al. Access to affordable medicines and diagnostic tests for asthma and COPD in sub Saharan Africa: the Ugandan perspective. BMC pulmonary medicine. 2017;17(1):179
18. World Health Organization. Monitoring the building blocks of health systems: a handbook of indicators and their measurement strategies: World Health Organization; 2010.
19. Government of the United Republic of Tanzania. STRATEGIC AND ACTION PLAN FOR THE PREVENTION AND CONTROL OF NON COMMUNICABLE DISEASES IN TANZANIA 2016 – 2020. Ministry of Health, Community Development, Gender, Elderly and Children

For peer review only

2016.04-03-2020
20. Ministry of Health. NATIONAL STRATEGIC PLAN FOR TUBERCULOSIS, LEPROSY AND LUNG HEALTH 2019-2023. NATIONAL TUBERCULOSIS, LEPROSY AND LUNG DISEASE PROGRAM; 2019.
21. English M, Irimu G, Agweyu A, Gathara D, Oliwa J, Ayieko P, et al. Building learning health systems to accelerate research and improve outcomes of clinical care in low-and middle-income countries. PLoS medicine. 2016;13(4):e1001991
22. Juma PA, Mohamed SF, Mwagomba BLM, Ndinda C, Mapa-Tassou C, Oluwasanu M, et al. Non-communicable disease prevention policy process in five African countries authors. BMC public health. 2018;18(1):961
23. Gilson L. Trust and the development of health care as a social institution. Social science & medicine. 2003;56(7):1453-68
24. De Savigny D, Adam T. Systems thinking for health systems strengthening: World Health Organization; 2009.
25. Sacks E, Morrow M, Story WT, Shelley KD, Shanklin D, Rahimtoola M, et al. Beyond the building blocks: integrating community roles into health systems frameworks to achieve health for all. BMJ global health. 2019;3(Suppl 3):e001384
26. Global Initiative For Asthma. Pocket guide for asthma management and prevention (For adults and children older than 5 years). In: GINA, editor. Fontana, USA 2020.
27. Pourbohloul B, Kieny M-P. Complex systems analysis: towards holistic approaches to health systems planning and policy. SciELO Public Health; 2011.
28. Maher A, Sridhar D. Political priority in the global fight against non–communicable diseases. Journal of global health. 2012;2(2)
29. McCollum R, Limato R, Otiso L, Theobald S, Taegtmeyer M. Health system governance following devolution: comparing experiences of decentralisation in Kenya and Indonesia. BMJ global health. 2018;3(5)
30. Barasa EW, Cleary S, English M, Molyneux S. The influence of power and actor relations on priority setting and resource allocation practices at the hospital level in Kenya: a case study. BMC health services research. 2016;16(1):536
31. Rabkin M, El-Sadr WM. Why reinvent the wheel? Leveraging the lessons of HIV scale-up to confront non-communicable diseases. Global public health. 2011;6(3):247-56

For peer review only

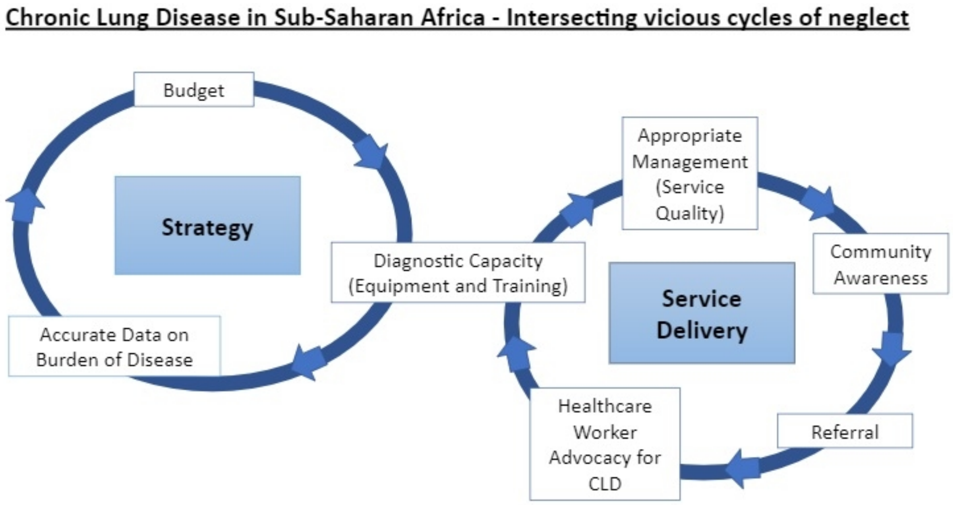

200x103mm (96 x 96 DPI)

Standards for Reporting Qualitative Research (SRQR)

O'Brien B.C., Harris, I.B., Beckman, T.J., Reed, D.A., & Cook, D.A. (2014). Standards for reporting qualitative research: a synthesis of recommendations. *Academic Medicine, 89(9)*, 1245-1251.

No. Topic	Item	Page
**Title and abstract**		
S1 Title	Concise description of the nature and topic of the study identifying the study as qualitative or indicating the approach (e.g., ethnography, grounded theory) or data collection methods (e.g., interview, focus group) is recommended	Title page
S2 Abstract	Summary of key elements of the study using the abstract format of the intended publication; typically includes objective, methods, results, and conclusions	Please refer to abstract page-2
**Introduction**		
S3 Problem formulation	Description and significance of the problem/phenomenon studied; review of relevant theory and empirical work; problem statement	Please refer to Page 3
S4 Purpose or research question	Purpose of the study and specific objectives or questions	Page 3
**Methods**		
S5 Qualitative approach and research paradigm	Qualitative approach (e.g., ethnography, grounded theory, case study, phenomenology, narrative research) and guiding theory if appropriate; identifying the research paradigm (e.g., positivist, constructivist/interpretivist) is also recommended	
S6 Researcher characteristics and reflexivity	Researchers' characteristics that may influence the research, including personal attributes, qualifications/experience, relationship with participants, assumptions, or presuppositions; potential or actual interaction between researchers' characteristics and the research questions, approach, methods, results, or transferability	Please refer to last sentence in the data collection section:<br><br>"Interviews were conducted by experienced qualitative researchers" pp4
S7 Context	Setting/site and salient contextual factors; rationale[a]	Context is described in 2nd paragraph of the

For peer review only

		introduction- pp3
S8 Sampling strategy	How and why research participants, documents, or events were selected; criteria for deciding when no further sampling was necessary (e.g., sampling saturation); rationale[a]	Methods section pp3. Healthcare workers and policy makers sampled for their roles in management of chronic lung diseases and policy making, respectively.
S9 Ethical issues pertaining to human subjects	Documentation of approval by an appropriate ethics review board and participant consent, or explanation for lack thereof; other confidentiality and data security issues	"Ethics" section, pp4.
S10 Data collection methods	Types of data collected; details of data collection procedures including (as appropriate) start and stop dates of data collection and analysis, iterative process, triangulation of sources/methods, and modification of procedures in response to evolving study findings; rationale[a]	Page 4, data collection section
S11 Data collection instruments and technologies	Description of instruments (e.g., interview guides, questionnaires) and devices (e.g., audio recorders) used for data collection; if/how the instrument(s) changed over the course of the study	Data collection, page 4
S12 Units of study	Number and relevant characteristics of participants, documents, or events included in the study; level of participation (could be reported in results)	Table 1 and 2
S13 Data processing	Methods for processing data prior to and during analysis, including transcription, data entry, data management and security, verification of data integrity, data coding, and anonymization/deidentification of excerpts	Intercountry analysis, page 4
S14 Data analysis	Process by which inferences, themes, etc., were identified and developed, including researchers involved in data analysis; usually references a specific paradigm or approach; rationale[a]	Please refer to the Intercountry analysis pp4, researcher roles listed under 'Author contributions' page 10
S15 Techniques to enhance trustworthiness	Techniques to enhance trustworthiness and credibility of data analysis (e.g., member checking, audit trail, triangulation); rationale[a]	Please refer to the Data collection section- verbatim transcripts (page 4); excerpts from transcripts used to illustrate specific findings (page 5)
**Results/Findings**		
S16 Synthesis and interpretation	Main findings (e.g., interpretations, inferences, and themes); might include development of a theory or model, or integration with prior research or theory	Findings section- data analysis uses the WHO health systems building blocks
S17 Links to empirical data	Evidence (e.g., quotes, field notes, text excerpts, photographs) to substantiate analytic findings	Verbatim quotes used in the Findings section

For peer review only

**Discussion**		
S18 Integration with prior work, implications, transferability, and contribution(s) to the field	Short summary of main findings; explanation of how findings and conclusions connect to, support, elaborate on, or challenge conclusions of earlier scholarship; discussion of scope of application/generalizability; identification of unique contribution(s) to scholarship in a discipline or field	Please refer to discussion section, pp 8
S19 Limitations	Trustworthiness and limitations of findings	Please refer to limitations section, page 9
**Other**		
S20 Conflicts of interest	Potential sources of influence or perceived influence on study conduct and conclusions; how these were managed	None declared
S21 Funding	Sources of funding and other support; role of funders in data collection, interpretation, and reporting	Role of funding source, page 4

[a]The rationale should briefly discuss the justification for choosing that theory, approach, method, or technique rather than other options available, the assumptions and limitations implicit in those choices, and how those choices influence study conclusions and transferability. As appropriate, the rationale for several items might be discussed together.

For peer review only

BMJ Open

What are the barriers to the diagnosis and management of chronic respiratory disease in sub-Saharan Africa? a qualitative study with healthcare workers, national and regional policy stakeholders in five countries

Journal:	*BMJ Open*
Manuscript ID	bmjopen-2021-052105.R1
Article Type:	Original research
Date Submitted by the Author:	04-Jan-2022

Complete List of Authors:	MULUPI, STEPHEN; Liverpool School of Tropical Medicine, International Public Health ; KEMRI, Centre for Respiratory Diseases Research<br>Ayakaka, Irene; Liverpool School of Tropical Medicine, International Public Health; Makerere University, Lung Institute<br>Tolhurst, Rachel; Liverpool School of Tropical Medicine, International Public Health<br>Kozak, Nicole; Liverpool School of Tropical Medicine, International Public Health ; REACH Trust Malawi<br>Shayo, Elizabeth; Liverpool School of Tropical Medicine, International Public Health ; National Institute of Medical Research, Tanzania<br>Abdalla, Elhafiz; Epi-Lab<br>Osman, Rashid; Epi-Lab<br>Egere, Uzochukwu; Liverpool School of Tropical Medicine<br>Mpagama, Stellah; 4. Kibong'oto Infectious Diseases Hospital/Kilimanjaro Christian Medical University<br>Chinouya, Martha; Liverpool School of Tropical Medicine, Department of International Public Health<br>Chikaphupha, Kingsley; REACH Trust, Malawi<br>Elsony, Asma; Epi-Lab<br>Meme, Helen; Kenya Medical Research Institute (KEMRI), Centre for Respiratory Diseases Research<br>Oronje, Rose; African Institute for Development Policy (AFIDEP)<br>Ntinginya, Nyanda; National Institute for Medical Research (NIMR)-Mbeya Medical Research Centre ,<br>Obasi, Angela; Liverpool School of Tropical Medicine, International Public Health ; Liverpool University Hospitals NHS Foundation Trust<br>Taegtmeyer, Miriam; Liverpool School of Tropical Medicine, International Public Health ; Liverpool University Hospitals NHS Foundation Trust, Tropical Infectious Diseases Unit

**Primary Subject Heading**:	Health services research
Secondary Subject Heading:	Health policy
Keywords:	Asthma < THORACIC MEDICINE, Health policy < HEALTH SERVICES ADMINISTRATION & MANAGEMENT, Protocols & guidelines < HEALTH SERVICES ADMINISTRATION & MANAGEMENT, EDUCATION & TRAINING

	(see Medical Education & Training), PRIMARY CARE, Chronic airways disease < THORACIC MEDICINE

SCHOLARONE™
Manuscripts

I, the Submitting Author has the right to grant and does grant on behalf of all authors of the Work (as defined in the below author licence), an exclusive licence and/or a non-exclusive licence for contributions from authors who are: i) UK Crown employees; ii) where BMJ has agreed a CC-BY licence shall apply, and/or iii) in accordance with the terms applicable for US Federal Government officers or employees acting as part of their official duties; on a worldwide, perpetual, irrevocable, royalty-free basis to BMJ Publishing Group Ltd ("BMJ") its licensees and where the relevant Journal is co-owned by BMJ to the co-owners of the Journal, to publish the Work in this journal and any other BMJ products and to exploit all rights, as set out in our licence.

The Submitting Author accepts and understands that any supply made under these terms is made by BMJ to the Submitting Author unless you are acting as an employee on behalf of your employer or a postgraduate student of an affiliated institution which is paying any applicable article publishing charge ("APC") for Open Access articles. Where the Submitting Author wishes to make the Work available on an Open Access basis (and intends to pay the relevant APC), the terms of reuse of such Open Access shall be governed by a Creative Commons licence – details of these licences and which Creative Commons licence will apply to this Work are set out in our licence referred to above.

Other than as permitted in any relevant BMJ Author's Self Archiving Policies, I confirm this Work has not been accepted for publication elsewhere, is not being considered for publication elsewhere and does not duplicate material already published. I confirm all authors consent to publication of this Work and authorise the granting of this licence.

For peer review only

What are the barriers to the diagnosis and management of chronic respiratory disease in sub-Saharan Africa? a qualitative study with healthcare workers, national and regional policy stakeholders in five countries

Authors: Stephen Mulupi*[1,2], Irene Ayakaka*[1,3], Rachel Tolhurst[1], Nicole Kozak[1,4], Elizabeth Shayo[1,5], Elhafiz Abdalla[6], Rashid Osman[6], Uzochukwu Egere[1], Stellah Mpagama[7], Martha J. Chinouya[1], Kingsley Chikaphupha[4], Asma El Sony[6], Hellen Meme[2], Rose Oronje[8], Nyanda Elias Ntinginya[5], Angela I. Obasi[1,9], Miriam Taegtmeyer[1,10]

*Joint first authors

Corresponding author: Stephen Mulupi

E-mail: stephen.mulupi@lstmed.ac.uk

Mobile phone number +254720640330

Affiliations:

1. Liverpool School of Tropical Medicine, Department of International Public Health, Liverpool, UK
2. Kenya Medical Research Institute, Centre for Respiratory Diseases Research (KEMRI-CRDR)
3. Makerere University Lung institute, (MLI)
4. REACH Trust, Malawi
5. National Institute for Medical Research, Tanzania
6. EpiLab, Sudan
7. Kibong'oto Infectious Disease Hospital, Tanzania
8. African Institute for Development Policy (AFIDEP)
9. AXESS Sexual Health, Liverpool University Hospitals NHS Foundation Trust, Liverpool, UK
10. Tropical Infectious Diseases Unit, Liverpool University Hospitals NHS Foundation Trust, Liverpool, UK

Contact information for authors:

1. Stephen Mulupi: stephen.mulupi@lstmed.ac.uk
2. Irene Ayakaka: irene.ayakaka@lstmed.ac.uk
3. Rachel Tolhurst: rachel.tolhurst@lstmed.ac.uk
4. Nicole Kozak: molemoe@gmail.com
5. Elizabeth Shayo: bshayo@yahoo.com
6. Elhafiz Abdalla: hafizhussein@gmail.com
7. Rashid Osman: rashildo@hotmail.com
8. Uzochukwu Egere: uzochukwu.egere@lstmed.ac.uk
9. Stellah Mpagama: sempagama@yahoo.com
10. Martha J. Chinouya: martha.chinouya@lstmed.ac.uk
11. Kingsley Chikaphupha: kchikaphupha@gmail.com.
12. Asma El Sony: asmaelsony@gmail.com
13. Hellen Meme: helenmeme@gmail.com
14. Rose Oronje: rose.oronje@afidep.org
15. Nyanda Elias Ntinginya: nelias@nimr-mmrc.org
16. Angela I. Obasi: Angela.Obasi@lstmed.ac.uk
17. Miriam Taegtmeyer: miriam.taegtmeyer@lstmed.ac.uk

On behalf of The NIHR International Multidisciplinary Programme to Address Lung Health and TB in Africa (IMPALA) Consortium: Emmanuel Addo-Yobo, Brian Allwood, Hastings Banda, Imelda Bates, Amsalu Binegdie, Adegoke Falade, Jahangir Khan, Maia Lesosky, Bertrand

For peer review only

Mbatchou, Kevin Mortimer, Beatrice Mutayoba, Louis Niessen, Jamie Rylance, S Bertel Squire, William Worodria, Heather Zar, Eliya Zulu, Lindsay Zurba

For peer review only

ABSTRACT

Objectives Chronic respiratory diseases (CRDs) are among the four main non-communicable diseases globally. The rising burden of CRD in low-and-middle-income countries will strain already weak health systems. This study investigated perspectives of healthcare workers and other health policy stakeholders on the barriers to effective diagnosis and management CRDs in Kenya, Malawi, Sudan, Tanzania, and Uganda.

Study design: qualitative descriptive

Settings Primary, secondary, and tertiary health facilities, government agencies, and civil society organisations in five sub-Saharan African countries.

Participants 60 national, and district-level policy stakeholders, and 49 healthcare workers, purposefully sampled for their policy decision-making or health provision roles. We conducted key-informant and in-depth interviews respectively, (2018-2019). Data were analysed through framework approach.

Results

We identified intersecting vicious cycles of neglect of CRD at strategic policy and healthcare facility levels. Lack of reliable data on CRD burden, due to weak information systems and diagnostic capacity, negatively affected CRD inclusion in policy, reflected by low budgetary allocations for services. Inadequate budgetary allocations (health facility level) constrained diagnostic capacity, quality of service delivery and compounded lack of routine data on CRD burden.

Conclusion

Health systems in the five countries are ill-equipped to respond to CRD; CRD are under-diagnosed, under-reported and underfunded leading to a vicious cycle of invisibility and neglect.

Strengths and limitations

Strengths

- The five studies were conducted independently by the country teams, allowing them to factor local health system issues.
- The inclusion of five countries with very similar emerging themes allows for comparison between countries and demonstrates how widespread barriers to CRD are.
- Triangulation of different study participants' perspectives enhances rigour in our analysis.

Limitations

- Differences in sampling the study sites e.g., number and levels of hospitals and rural/ urban variations limited comparisons.
- We were not able investigate patients' perspectives in demanding and accessing health services for CRDs.

For peer review only - http://bmjopen.bmj.com/site/about/guidelines.xhtml

1. Introduction

Chronic respiratory disease (CRD) accounts for approximately 4 million deaths per year world-wide[1, 2]. Globally the most prevalent CRDs are asthma and chronic obstructive pulmonary disease (COPD) [3] but the definition includes other non-infectious lung and airway disease such as bronchiectasis and post-TB lung disease. The burden of CRD morbidity and mortality is rising steeply in low- and middle-income countries (LMICs)[4, 5]. The high prevalence of CRD in sub-Saharan Africa (SSA) is driven in part by high rates of recurrent childhood respiratory infections and pulmonary TB which are important precursors of CRD[6-9]. A hospital-based study in Tanzania among post-TB patients estimated 74% prevalence of abnormal lung function[10]. Other common risk factors include exposure to tobacco smoke, indoor pollution from biomass fuels and occupational exposures in the mining industry, sugar, and tobacco plantations[11, 12]. In Uganda, the age-adjusted prevalence for any chronic respiratory condition in both rural and urban settings is 20% [13] and in Malawi over 40% of adults randomly sampled in an urban population had abnormal lung function[14].

The management of CRD in SSA is undermined by weak health systems characterised by lack of appropriate medical equipment and low diagnostic capacity[15, 16]. Healthcare workers trained in the management of CRD are scarce and medicines are either unavailable or unaffordable by many patients[17, 18]. Existing lung health pathways in SSA focus on TB with smear negative patients repeatedly visiting health facilities or being referred from one facility to another seeking definitive diagnosis. A wide range of policy design, implementation and service delivery gaps undermine CRD care pathways. Importantly, there is limited understanding in how specific policy and health facility level factors interact and shape health service delivery in SSA contexts.

In this qualitative study, we sought to understand and characterize the views and perspectives of policymakers and healthcare workers on CRD policies and services in five countries in SSA. We present the findings that show clear linkage of policy-level factors to service delivery experiences. We contribute evidence to inform the design of future policy interventions to strengthen the delivery of services and ultimately improve CRD management in similar settings. The five linked studies formed baseline assessments for country-level research within the National Institute for Health Research (NIHR), International Multidisciplinary Programme to Address Lung Health and TB in Africa (IMPALA[1]).

Methods

Five studies were independently conducted in Kenya, Malawi, Sudan, Tanzania, and Uganda between 2018 and 2019 based on a constructivist philosophical paradigm using qualitative study methods[19, 20] to collect data and to perform data validation, analysis and interpretation to answer the study questions. Ministry of Health officials and other healthcare policy stakeholders were purposively sampled for their roles and experiences in policy design and implementation alongside health workers experienced in the management of CRD (Table 1).To secure interview appointments participants were contacted through e-mail, phone or verbally, as appropriate.. Public healthcare facilities were selected to include both urban and rural settings and different facility levels (Table 2). All served populations with high prevalence rates of TB and other lung conditions.

[1] https://www.lstmed.ac.uk/impala

For peer review only - http://bmjopen.bmj.com/site/about/guidelines.xhtml

Table 1: The distribution of participants across the five case study countries.

Informants	Kenya	Malawi	Sudan	Tanzania	Uganda
National, regional and district level policy stakeholders	15	13	14	13	5
Health care workers	14	5*	14	10	6
**Total**	**29**	**18**	**28**	**23**	**11**

***Senior clinicians**

Table 2: Distribution of healthcare facilities in the five case study countries

Public health system level	Kenya	Malawi	Sudan	Tanzania	Uganda	Total
Primary healthcare facilities (health centre and dispensary level)	3	1	2	8	6	20
District level hospitals	1	1	8	1	0	11
National/regional referral hospitals	0	2	0	1	2	5
**Total number of facilities**	**4**	**4**	**10**	**10**	**8**	**36**

Role of the funding source

This research was funded by the (NIHR IMPALA, grant reference 16/136/35) using UK aid from the UK Government to support global health research. The views expressed in this publication are those of the author(s) and not necessarily those of the NIHR or the UK Department of Health and Social Care.

Data collection

Key informant interviews (KIIs) with policy stakeholders explored opinions about prioritization of CRD; (un)availability and operationalization of CRD policies; systemic factors enhancing or impeding provision of healthcare services for CRD; and opinions on how systems could be improved. In-depth interviews with healthcare workers, explored their experiences in diagnosing and managing CRD; availability of diagnostic equipment and medicines; experience of training; and their perceptions of what has worked well or not, in management of CRD. Semi-structured interview guides (Appendices 1-6) were developed broadly aligning with the health systems' building blocks[21]. Interviews were conducted May 2018 to March 2019, by experienced qualitative researchers in English, or the local languages as appropriate, and digitally recorded with consent. These face-to-face interviews took place in quiet, private rooms within the workplaces of the participants, to protect participants' confidentiality, minimize disruptions and ensure quality of audio recordings. The discussions took around 40 minutes. The researchers in Sudan, Malawi and Tanzania, had no prior relationships with the participants. In Malawi a female white Canadian researcher (NK) conducted some of the interviews (others were done by a Chichewa speaking research assistant) and came from an outsider perspective. The researcher from Uganda (IA) is a respected female medical doctor who was known to some of the participants. This positionality required critical reflection on being an "insider" to the health system during the research period. Similarly in Kenya, affiliation of the male researcher (SM) to the Kenya Medical Research Institute, a government agency, may have shaped trust and response to the study, though the effect on issues is unknown. The researchers jotted notes about participants' comments and the researcher's own thoughts during the interviews; they used memoing as soon as possible after an interview and during the transcription of recordings. Recordings

For peer review only - http://bmjopen.bmj.com/site/about/guidelines.xhtml

were transcribed verbatim and translated to English for analysis. Field notes complemented the audio recordings.

Intercountry analysis.

Data collection was preceded by a common training in policy (2018) for the researchers and followed by joint analysis meetings that discussed similarities and differences between country-specific study findings; the codes and topics were identified within each country and combined during analysis and the respective country teams re-read transcripts to confirm accuracy and identify emerging themes. Authors SM, IA, RT, MT, ES, participated in the coding meetings and determination of the final codebook. Initial codebooks were developed from the broad interview guide topics, then updated inductively as novel codes emerged. A final codebook was discussed, further refined, and applied to the data, using the framework approach [22], with analysis supported by NVivo™ v11.0 (QSR International 1999) software. An exploration of emerging patterns led to the identification of final themes with selected quotes used to illustrate specific findings.

Ethics

The Liverpool School of Tropical Medicine Ethics Committee approved these studies separately (Kenya: protocol 18-054; Uganda: protocol 18-037; Malawi: protocol M1803; Tanzania and Sudan; protocol 18-043). Additionally, each approval was by in-country committees[2]. All participants gave written, informed consent.

Findings

The World Health Organization (WHO), in 2007 proposed [a framework describing health system functions in terms of six core interdependent components or "building blocks"]: (i) service delivery; (ii) health workforce; (iii) health information systems; (iv) access to essential medicines; (v) financing; and (vi) leadership/governance[21]. Effective coordination, and optimal performance of these health system blocks would theoretically enhance achievement of health system goals, i.e. "improving health and health equity, in ways that are responsive, financially fair, and make the best, or most efficient, use of available resources"[21]. We used the WHO health systems building blocks to frame our exploration at strategic and service delivery levels. A picture emerged of neglect of CRD at each level of the systems and for each building block with consequences across the whole system.

Strategic level

Variable availability and awareness of policy

CRD policy availability varied between countries. Kenya and Tanzania have fully developed and adopted policy strategies for lung health[23, 24] and the national and district stakeholders were aware of this; however, few health care workers were aware of the policies or where to find them. There was no stand-alone policy for CRD in Uganda and in Malawi; CRD was embedded within NCD policy, which was in draft, with limited awareness among all levels of participants. In Sudan, there was no specific policy for CRD.

> *"I do not know whether there is an existing policy for TB and CRD. There is a general strategy; the national health policy that generally addresses chronic diseases." (KII-F, Sudan Federal-MoH official)*

Lack of CRD data

[2] Kenya Medical Research Institute (KEMRI/SERU/CRDR/037/3717); Malawi National Health Science Research committee (Protocol # 18/04/2021); Sudan: Ref 44/T/Kh/1; Tanzania: Medical Research Coordinating Committee of the National Institute for Medical Research, (NIMR/HQ/R.8a/V.IX/2922) and Uganda (TASO) IRB:TASOREC /030/18-UG-REC-009 and the Uganda National Council for Science and Technology HS232ES).

For peer review only - http://bmjopen.bmj.com/site/about/guidelines.xhtml

In all five contexts, policy stakeholders associated the limited data on burden of disease with low visibility and low domestic budget allocations for CRD as shown by this typical quote:

> *"For COPD we don't have data, we don't know its prevalence, we don't know its incidence, we don't know its mortality" (IDI national informant, Tanzania).*

None of the study countries had comprehensive population surveys on CRD, but Sudan, Kenya and Uganda reported research evidence about asthma, COPD and the growing burden of CRD. In Malawi, participants noted that CRD was not included in the recent nationwide burden of disease study and went further to highlight the lack of data on cost-effectiveness.

> *" So [CRDs] never feature in the cost effectiveness analyses that allow for their prioritization, benchmarked against the other priorities where there is evidence….for chronic respiratory disease we have to recognize that the evidence base for the country is very tiny. So being able to advocate beyond, the NCD Department into the Treasury, into the Health Sector Strategic Plan, is multiple steps away" (Malawi-KI 18, researcher).*

Neither was routine data able to fill the evidence gaps due to a lack of appropriate data collection tools and shortages of health information and records officers. Participants in all the five countries emphasized the need to address these gaps in developing investment cases for CRD at national level.

> "*You perform a study and show them the results. How many are affected? How many are disabled? How many lose their jobs? How many houses lose support? If you provide such work, you might be able to convince the (national treasury) officials*" *(KII-S5, Gezira State, Sudan MoH official).*

Lack of donor prioritisation decreases budgetary allocation

The health system financing arrangements in all the study countries were reported as being heavily dependent on external partners. CRD was perceived to be given low priority by donors because of their generally non-infectious nature and the perception that they are less fatal than infectious diseases such as malaria, HIV/AIDS and TB, and maternal and child health conditions. Even within the NCD departments in the study countries, efforts and resources are majorly directed towards cancer, diabetes, hypertension and other cardiovascular diseases.

> *"We have some reasonable data on the burden of asthma but asthma doesn't do one thing, doesn't kill people that much and so because it doesn't kill, it doesn't get the attention it should get… everybody gets worried about things that kill (TB, Malaria, HIV/AIDS, Diabetes, Cancer)and not those that give you chronic illness" (Kenya- KII-KD)*

Current priorities

Positive steps in data collection and policy development were also described. In response to a global spotlight on NCDs and recent increased donor interest, the Kenyan and Tanzanian governments plan to close the evidence gap by capturing CRD data in routine health facility tools, and subsequent nationwide surveys like the Demographic and Health Surveys.

> *"We are refining the tools to better capture the NCDs and have specific NCD registers like in the general outpatient settings (KII, Kenya-MOH, national level)*

At the time of data collection, Kenya was in the process of ratifying the Protocol to eliminate illicit trade in tobacco products, and Uganda had banned tobacco smoking in public, tobacco advertising, promotion and sponsorship, and limited sales. This suggests a heightened prioritization of an important risk-factor of CRD on the policy agenda, with potential for downstream policy interventions.

For peer review only - http://bmjopen.bmj.com/site/about/guidelines.xhtml

Service level

Key service level barriers arising from policy failures included challenges in accessing CRD diagnostics and medicines, limited reporting, and low confidence and skill in diagnosis and management. There was a disconnect between the perceptions of policy stakeholders, which reflected what was available on paper, and the front-line health workers, who reported feeling ill-equipped to attend patients in practice.

Lack of diagnostic equipment

Participants universally agreed that the entry point to CRD diagnosis in their TB-endemic contexts required the exclusion of active TB disease in chronic cough. Diagnostic capacity was defined as a combination of sputum screening, with the availability of radiology, lung function testing, skilled staff and diagnostic and treatment algorithms for the common CRDs (asthma, post TB lung disease and COPD). While each study country had chest clinics at tertiary hospitals with some level of equipment and expertise, only TB screening had been systematically decentralised at lower levels.

Challenges were described in each aspect of diagnosis, with patients often paying out-of-pocket costs. All countries reported delays in sputum screening. In some instances, these delays extended to more than a week and were associated with additional costs to patients and subsequent loss to follow up.

Finally, the widespread lack of (or dysfunctional) x-ray equipment and lack of qualified personnel reported in lower-level public facilities in Kenya, Malawi, Uganda, and Tanzania, meant that patients had to seek chest x-rays (and CT scans) from private healthcare providers, whose costs were unaffordable to most. The lack of peak flow meters and spirometry machines meant that patients requiring lung function testing were referred to the national teaching and referral hospitals.

> *"Yeah, there was a time, we also [provided spirometry] but …our machine broke down … when we see they are needing that spirometry we send them to Kenyatta National Hospital (Healthcare Worker, Kenya).*

In Tanzania, participants noted that despite the recommendation to have peak flow meters at the primary healthcare level, none were available and not a single facility (including the referral hospital) offered spirometry. In Kenya, Malawi, Sudan, and Uganda, the use of spirometry was reported to be limited to tertiary hospitals with international research collaborations, where training and equipment are maintained through grants from external partners. Although some policy level stakeholders thought otherwise, health workers at the lower-level hospitals in all countries expressed dissatisfaction with the lack of peak flow meters and spirometers. In Malawi, there were conflicting reports on the distribution of spirometry services across facilities. Whilst policymakers claimed that all tertiary hospitals had spirometers, healthcare workers reported that spirometers were only available at one teaching hospital as part of a research project.

Lack of CRD training and guideline dissemination

According to majority of key informants in Kenya, Uganda, and Tanzania most healthcare workers had not received training on the latest diagnostic procedures for CRD, including the use of spirometry:

> *"Honestly, we have not received any training for CRD; we used to go for TB trainings only" (IDI, Healthcare worker, Tanzania)*

Malawian participants described a successful pilot decentralising CRD training, but this was yet to be scaled-up nationally. Participants in all contexts emphasized the need improve in-service training countrywide. In-service training opportunities were *ad hoc* and often *'erratic'* (Sudan); mostly supported by specific programmes, including research projects (e.g. Uganda), non-governmental organizations and pharmaceutical companies promoting specific products; and characterised by low coverage of necessary staff.

> *"…we started a program with some collaborative partners, … they bring health workers from some districts, and we come and do modules to understand for example what asthma is, how*

For peer review only - http://bmjopen.bmj.com/site/about/guidelines.xhtml

its diagnosed what is the best treatment, because in [medical] school frankly, people don't learn so much for the benefit of the wider community (MOH consultant physician Uganda)."

Most of the available training focused on TB. In Tanzania this was to the exclusion CRD. In Kenya, CRD were added on to TB training. Healthcare workers in Kenya reported difficulties in accessing professional training, within the devolved government setup. Lack of financial sponsorship from government, and a requirement that they resign if they applied for fulltime study imposed steep opportunity costs.

Standard processes of communicating guidelines from national policy level to service delivery levels were felt to be inadequate; for example, healthcare workers in Kenya described learning about asthma management guidelines incidentally, during internet use or informal discussions with colleagues. There was limited awareness of treatment guideline updates with many clinicians still prescribing available oral salbutamol instead of inhalers, despite lack of evidence for efficacy. Likewise, in Tanzania, although the CRD guidelines were available on the Ministry of Health website and copies were seen at the sites, participants seemed unaware of their content.

Limited availability of drugs and lack of confidence in management

Restrictive policy guidelines and user-fees imposed barriers to accessing corticosteroid inhalers. For example, beclomethasone is classified as a central facility level medicine in Malawi, that required international procurement protocols prone to delays of 4-7 months. In Tanzania only bronchodilators, injectable and oral steroids for acute asthma are allowed at lower levels of service provision. Similarly, in Uganda some medicines were restricted at the primary level. In contrast in Kenya bronchodilators and corticosteroids have been included in pharmacy order forms for primary health care facilities where they are provided free, implying shifts in practice to enhance access. In Sudan, asthma medications including inhalers and corticosteroids are supposed to be provided free of charge especially for patients in acute attacks. However, these are often not available in emergency rooms at both district and national levels, and when available, they are not always free of charge.

"Some of the services for asthma might be provided for free in the emergency room, but the majority of it is not provided for free although it should [be]" (KII-S4, Gezira State MoH official).

Additionally, respondents from all five countries reported frequent drug stockouts even of the few CRD treatments included on essential medicines lists. At the time of data collection, the Kenya Medical Supplies Authority had stopped supplies to counties with outstanding bills. At primary care level in Tanzania and Uganda drugs other than oral salbutamol were unavailable.

Frontline clinicians at primary and district level felt ill-equipped to meet the complexity of cases. Healthcare workers explained that they lacked confidence in managing chronic CRD, although acute asthma attacks would be treated (nebulisers, oral aminophylline and adrenalin injections were all mentioned).

"We end up using treatments we are not supposed to (for example oral salbutamol) because that is what is available (Uganda - Clinical officer HC III).

Key informants in Kenya and Uganda, mentioned measures to enhance treatment capacity through review of the curricula for in-service training of clinical and medical officers, and for Kenya, the development of 'module 13' for community health volunteers, on identifying and managing asthma.

"We are working closely with KMTC (Kenya Medical Training College) itself, the Kenya Clinical Officers Council and the Nursing Council of Kenya to give NCD's more prominence in the pre-service training (KII_MOH, Kenya)

Most healthcare workers, however referred chronic asthma and other undiagnosed CRD conditions to higher level facilities, but even at this level limited capacity for management was described. All five countries reported very few pulmonologists. Malawi for example, had only one specialist respiratory clinician, who often did not receive referrals because of low awareness among healthcare workers.

Poor reporting and reduced health care worker awareness

For peer review only - http://bmjopen.bmj.com/site/about/guidelines.xhtml

Low healthcare workers' diagnostic capacities implied that subsequent data inputs into the health information system may not provide reliable evidence of the CRD disease burden. Furthermore, the appropriate coding of diseases overall, is undermined by shortage of health information officers, and essential reporting tools.

> *"...then you find most of our medical personnel record "chronic cough" which is not a diagnosis ... we are championing for this cadre of staff (health records information officers) because they are very critical otherwise, we would continue making wrong decisions based on poor data (KII-Non-State Actor, Kenya)*

This poor reporting was felt in turn to reduce awareness of CRD among health care workers of the need for clinical follow-up and of the overall scale of the problem.

DISCUSSION

Based on data from five Sub-Saharan African countries (Kenya, Malawi, Sudan, Tanzania, and Uganda) we identified how neglect of CRD at strategic and service levels form two intersecting vicious cycles as illustrated in Figure 1.

Figure 1

Whilst specific weaknesses varied by context, common cycles were discernible. At strategic policy level, low diagnostic capacity, weak recording and reporting systems limited the availability of reliable data on the burden of lung diseases, negatively affecting inclusion in policy, and in turn budgetary allocations for diagnostic equipment, training, and medicines. At the service level, lack of budgetary allocations for equipment and training of staff constrained diagnostic capacity, which, along with limited availability of appropriate medicines reduced service delivery quality and collection of appropriate data within healthcare facilities. Providers felt frustrated at their lack of capacity to diagnose and treat these conditions; they were aware that that their own skills to support patients with CRD over time were limited.

To strengthen health systems in these countries and others in similar contexts, it will be important to break these vicious cycles,[25] although the most strategic entry points may vary by country. For example, variations in the development of policy strategies specific to lung health suggest policy change is more important in some contexts with NCD policy and TB control policy both providing possible avenues of entry[26]. Improvements in diagnostic capacity, along with improving accuracy of reporting, may create impetus for policy commitments and corresponding budgetary allocations, required to improve availability of medicines. Concurrent improvements in systems 'software' such as communication, advocacy, and accountability are required to realise policy changes in practice[27, 28]. For example, strategic communication of local data and accountability for responsiveness to this is needed, not only on burden of disease and service use but also on stockouts, broken equipment, or vacant posts[29]. Ultimately a team approach (including managers, clinical providers, health information officers and community actors) will be needed to ensure a coherent approach and feed-back loops. The World Health Organization, and civil society groups, such as professional thoracic societies and researchers have an important role of developing a sustainable integrated response through their guidelines and practice in LMIC contexts. Effective communication of guidelines and protocols needs to be accompanied by operational plans to enact them. For example, it is now clear that treating asthma patients with short acting beta agonists alone is associated with risk (exacerbations and thus likely deaths) even in mild asthma. This has led to a revision of the current Global Initiative for Asthma (GINA) guidelines for asthma control advocate use of inhaled corticosteroid-formoterol for the management of mild asthma and yet many of the study countries restrict availability at the primary health care facilities most likely to see cases[30].

It is likely to be too simplistic to assume that changes at one point in the cycle will lead to the desired changes in a linear way throughout the cycle, since challenges in a complex system are likely to be

For peer review only - http://bmjopen.bmj.com/site/about/guidelines.xhtml

multi-causal[31]. Evidence is only one, often small, element in policymaking[32]. In turn, written policy may not be translated into budgetary allocations or materialise as equipment and medicine availability due to intense budgetary competition; the role of donor priorities; 'leakage' of resources through the system; procurement and maintenance challenges, amongst others. Decentralisation offers opportunities for local (burden of disease) data and demands to influence budgetary allocations, and for enhancing primary healthcare services bringing CRD services closer to patients' residence. Decentralization may however also mean greater political struggles over priorities[33, 34] and increased fragmentation across the national health system. In many contexts, inadequate human resources and high staff attrition rates in rural areas provides a significant challenge to maintaining trained providers. Interventions in one part of the system may have unintended consequences in another[28]; where provider workloads are high, improved diagnostic capacity for certain diseases may deprioritise others, including by increasing service utilisation. Efforts will therefore be needed to track changes throughout the system, and beyond CRDs.

Finally, management of CRD, like other chronic conditions, requires continuity of care, effective linkage between health facilities and community systems, and patient empowerment[12, 35]. Significant communication is therefore required by providers, including community health workers, to increase awareness of CRD and support patients with transition to self-management of their conditions.

Health systems in LMICs are currently designed to respond to acute, mostly communicable, illnesses usually through disease-specific vertical programmes. Despite some adaptation to treat people living with HIV, these systems often respond poorly to chronic conditions such as CRD. Key features of care models for other non communicable diseases which could be adapted are having ear marked funding for organized and equipped healthcare teams, sustained supply of medicines, continuity of care, and strong linkages between healthcare facilities and community health systems. Integration of policy and services has benefits of a more holistic approach in addressing multimorbidity and can for example reduce poor prescribing practice [36]. Rather than adding a new vertical programme, or a new set of diseases to existing programmes, we advocate for a system-wide approach to a range of chronic diseases.

Strengths and limitations

The main strengths and limitations both relate to the fact that the five studies were conducted independently, by different research teams with variations in the topic guides allowing for contextual insights. This allowed us to compare and contrast local strategies in dealing with CRD and share experiences and learn from them. The KII and IDI are appropriate in investigating and triangulating perspectives of study participants, enhancing rigour in the data collection. A larger number of district hospitals were sampled in Sudan and a focus on urban sites in Kenya, Malawi and Uganda with the inclusion of rural sites in Tanzania, and Sudan. While these independently conducted studies established similar findings in diagnosis and management of CRD, suggesting important systemic challenges across the SSA countries, the findings should nevertheless be interpreted with caution, as environmental exposures and service delivery contexts may differ with the distribution of healthcare facilities and professionals relatively higher in urban settings. We did not include CRD patients, community health workers and at-risk community members' perspectives in our study but focused on the overall, issues that affect the planning and care of the patients. Due to constraints in data collection, saturation was not achieved in the Ugandan site.

Recommendations and conclusions

The COVID-19 pandemic has brought into sharp focus the urgent need to reform and strengthen healthcare systems to effectively respond to people with chronic health conditions, including CRD, an important risk factor to COVID-19 severity. In the five SSA countries studied, interlinked gaps at the policy strategic level, and healthcare delivery levels, undermine appropriate provision of services for people with CRD. Lack of diagnostic capacity is a major link between the vicious cycles, influencing both the ability to manage cases within services and lack of accurate data on CRD to inform policy responses and resource allocation. Improvements are required across all key elements of the service

For peer review only - http://bmjopen.bmj.com/site/about/guidelines.xhtml

delivery systems, including pre- and in-service training for diagnosis and management, guideline dissemination, diagnostic equipment, recording and reporting. Additionally, there is urgent need to enhance reliable, affordable access to drugs, particularly the inhaled corticosteroid-formoterol inhaler in the early stages of asthma management in children, adolescents, and adults[37]. Enhancing collection of population-level data to ascertain the true burden of disease may be an important entry point to drive policy change ensuring that people living with CRD are not 'left behind' in the development of universal healthcare.

For peer review only

For peer review only - http://bmjopen.bmj.com/site/about/guidelines.xhtml

Author contributions

1. Stephen Mulupi- literature search, data collection, analysis, interpretation, writing
2. Irene Ayakaka: literature search, data collection & analysis, interpretation, writing
3. Nicole Kozak: literature search data collection & analysis, writing
4. Elizabeth Shayo: Data collection & analysis, writing
5. Hafiz Hussein: Data collection & analysis, writing
6. Rashid Osman: Data collection & analysis, writing
7. Stella Mpagama: Data collection & analysis, writing
8. Rachel Tolhurst: Study design, data analysis, interpretation, writing
9. Uzochukwu Egere: Study design, interpretation, writing
10. Martha Chinouya: Study design, interpretation, writing
11. Kingsley Chikaphupha: data analysis, interpretation, writing
12. Asma El Sony: Interpretation, writing
13. Hellen Meme: Interpretation, writing
14. Rose Oronje: Interpretation, writing
15. Nyanda Elias Ntinginya: Interpretation, writing
16. Angela Obasi: Study design, Interpretation, writing
17. Miriam Taegtmeyer: Study design, data analysis, interpretation, writing

peer review only

Declaration of competing interests
None declared

Patient and Public involvement:

Patients or the public were NOT involved in the design, or conduct, or reporting, or dissemination plans of our research

For peer review only - http://bmjopen.bmj.com/site/about/guidelines.xhtml

References

1. World Health Organization. Non communicable diseases, Fact sheet. In: WHO, editor. 2017.
2. Soriano JB, Abajobir AA, Abate KH, Abera SF, Agrawal A, Ahmed MB, et al. Global, regional, and national deaths, prevalence, disability-adjusted life years, and years lived with disability for chronic obstructive pulmonary disease and asthma, 1990–2015: a systematic analysis for the Global Burden of Disease Study 2015. The Lancet Respiratory Medicine. 2017;5(9):691-706
3. Soriano JB, Kendrick PJ, Paulson KR, Gupta V, Abrams EM, Adedoyin RA, et al. Prevalence and attributable health burden of chronic respiratory diseases, 1990–2017: a systematic analysis for the Global Burden of Disease Study 2017. The Lancet Respiratory Medicine. 2020;8(6):585-96
4. Marais BJ, Loennroth K, Lawn SD, Migliori GB, Mwaba P, Glaziou P, et al. Tuberculosis comorbidity with communicable and non-communicable diseases: integrating health services and control efforts. The Lancet infectious diseases. 2013;13(5):436-48
5. Samb B, Desai N, Nishtar S, Mendis S, Bekedam H, Wright A, et al. Prevention and management of chronic disease: a litmus test for health-systems strengthening in low-income and middle-income countries. The Lancet. 2010;376(9754):1785-97
6. World Health Organization. Global Tuberculosis Report, 2019. Geneva: World Health Organization; 2019.
7. Byrne AL, Marais BJ, Mitnick CD, Lecca L, Marks GB. Tuberculosis and chronic respiratory disease: a systematic review. International Journal of Infectious Diseases. 2015;32:138-46
8. Amaral AF, Coton S, Kato B, Tan WC, Studnicka M, Janson C, et al. Tuberculosis associates with both airflow obstruction and low lung function: BOLD results. European Respiratory Journal. 2015;46(4):1104-12
9. Menezes AMB, Hallal PC, Perez-Padilla R, Jardim J, Muino A, Lopez M, et al. Tuberculosis and airflow obstruction: evidence from the PLATINO study in Latin America. European Respiratory Journal. 2007;30(6):1180-5
10. Manji M, Shayo G, Mamuya S, Mpembeni R, Jusabani A, Mugusi F. Lung functions among patients with pulmonary tuberculosis in Dar es Salaam – a cross-sectional study. BMC Pulmonary Medicine. 2016;16(1):58
11. Banda HT, Thomson R, Mortimer K, Bello GA, Mbera GB, Malmborg R, et al. Community prevalence of chronic respiratory symptoms in rural Malawi: Implications for policy. PloS one. 2017;12(12):e0188437
12. Bischoff A, Ekoe T, Perone N, Slama S, Loutan L. Chronic disease management in Sub-Saharan Africa: whose business is it? International Journal of Environmental Research and Public Health. 2009;6(8):2258-70
13. Siddharthan T, Grigsby M, Morgan B, Kalyesubula R, Wise RA, Kirenga B, et al. Prevalence of chronic respiratory disease in urban and rural Uganda. World Health Organization Bulletin of the World Health Organization. 2019;97(5):318-27
14. Meghji J, Nadeau G, Davis KJ, Wang D, Nyirenda MJ, Gordon SB, et al. Noncommunicable lung disease in sub-Saharan Africa. A community-based cross-sectional study of adults in urban Malawi. American journal of respiratory and critical care medicine. 2016;194(1):67-76
15. van Gemert F, van der Molen T, Jones R, Chavannes N. The impact of asthma and COPD in sub-Saharan Africa. Primary Care Respiratory Journal. 2011;20(3):240
16. Arokiasamy P, Kowal P, Capistrant BD, Gildner TE, Thiele E, Biritwum RB, et al. Chronic noncommunicable diseases in 6 low-and middle-income countries: findings from wave 1 of the World Health Organization's study on global Ageing and adult health (SAGE). American journal of epidemiology. 2017;185(6):414-28
17. Kibirige D, Sanya RE, Nantanda R, Worodria W, Kirenga B. Availability and affordability of medicines and diagnostic tests recommended for management of asthma and chronic obstructive pulmonary disease in sub-Saharan Africa: a systematic review. Allergy, Asthma & Clinical Immunology. 2019;15(1):14

For peer review only - http://bmjopen.bmj.com/site/about/guidelines.xhtml

18. Kibirige D, Kampiire L, Atuhe D, Mwebaze R, Katagira W, Muttamba W, et al. Access to affordable medicines and diagnostic tests for asthma and COPD in sub Saharan Africa: the Ugandan perspective. BMC pulmonary medicine. 2017;17(1):179
19. Crotty M. Introduction: The research process. The foundations of social research: Meaning and perspective in the research process. 1998:1-17
20. Scotland J. Exploring the philosophical underpinnings of research: Relating ontology and epistemology to the methodology and methods of the scientific, interpretive, and critical research paradigms. English language teaching. 2012;5(9):9-16
21. World Health Organization. EVERYBODY'S BUSINESS. STRENGTHENING HEALTH SYSTEMS TO IMPROVE HEALTH OUTCOMES: WHO'S FRAMEWORK FOR ACTION. 2007
22. Ritchie J, Spencer L, Bryman A, Burgess RG. Analysing qualitative data. 1994.
23. Government of the United Republic of Tanzania.

Strategic and Action Plan for the Prevention And Control of Non Communicable Diseases in Tanzania 2016 – 2020. Ministry of Health, Community Development, Gender, Elderly and Children

2016.04-03-2020
24. Ministry of Health. National Strategic Plan for Tuberculosis, Leprosy and Lung Health 2019-2023. National Tuberculosis, Leprosy and Lung Disease Program; 2019.
25. English M, Irimu G, Agweyu A, Gathara D, Oliwa J, Ayieko P, et al. Building learning health systems to accelerate research and improve outcomes of clinical care in low-and middle-income countries. PLoS medicine. 2016;13(4):e1001991
26. Juma PA, Mohamed SF, Mwagomba BLM, Ndinda C, Mapa-Tassou C, Oluwasanu M, et al. Non-communicable disease prevention policy process in five African countries authors. BMC public health. 2018;18(1):961
27. Gilson L. Trust and the development of health care as a social institution. Social science & medicine. 2003;56(7):1453-68
28. De Savigny D, Adam T. Systems thinking for health systems strengthening: World Health Organization; 2009.
29. Sacks E, Morrow M, Story WT, Shelley KD, Shanklin D, Rahimtoola M, et al. Beyond the building blocks: integrating community roles into health systems frameworks to achieve health for all. BMJ global health. 2019;3(Suppl 3):e001384
30. Global Initiative For Asthma. Pocket guide for asthma management and prevention (For adults and children older than 5 years). In: GINA, editor. Fontana, USA 2020.
31. Pourbohloul B, Kieny M-P. Complex systems analysis: towards holistic approaches to health systems planning and policy. SciELO Public Health; 2011. p. 242-.
32. Maher A, Sridhar D. Political priority in the global fight against non–communicable diseases. Journal of global health. 2012;2(2)
33. McCollum R, Limato R, Otiso L, Theobald S, Taegtmeyer M. Health system governance following devolution: comparing experiences of decentralisation in Kenya and Indonesia. BMJ global health. 2018;3(5):e000939
34. Barasa EW, Cleary S, English M, Molyneux S. The influence of power and actor relations on priority setting and resource allocation practices at the hospital level in Kenya: a case study. BMC health services research. 2016;16(1):536
35. Rabkin M, El-Sadr WM. Why reinvent the wheel? Leveraging the lessons of HIV scale-up to confront non-communicable diseases. Global public health. 2011;6(3):247-56
36. Epping-Jordan J, Pruitt S, Bengoa R, Wagner EH. Improving the quality of health care for chronic conditions. BMJ Quality & Safety. 2004;13(4):299-305
37. Reddel HK, Bacharier LB, Bateman ED, Brightling CE, Brusselle GG, Buhl R, et al. Global Initiative for Asthma (GINA) Strategy 2021–Executive summary and rationale for key changes. The Journal of Allergy and Clinical Immunology: In Practice. 2021

For peer review only - http://bmjopen.bmj.com/site/about/guidelines.xhtml

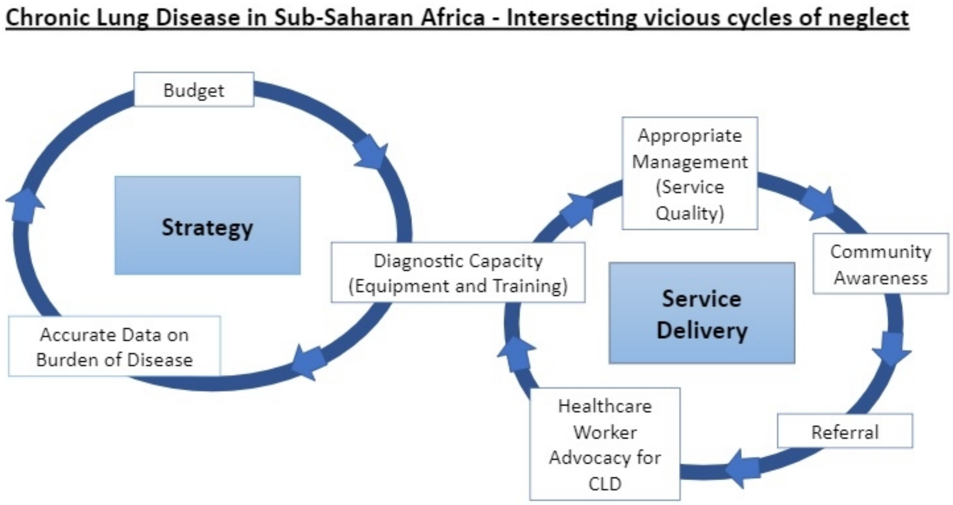

200x103mm (96 x 96 DPI)

Appendix 1_ In-depth interview for healthcare workers: clinical staff e.g. nurse, facility in-charge, CRD specialist

1. Please describe to me your roles in this department (probe: how long have you been in this role? How big is your team? Please describe their roles?)

2. Do you see patients with chronic respiratory diseases? What conditions do you mostly see? How would you know if someone has CRD?. What challenges do you face in diagnosing CRD?

3. What makes people with chronic respiratory symptoms come here? (probe- are they referred here? From where? Who refers them?) Please describe what happens to these patients from the time they come in. (Probe: Screening and diagnostics, treatment, follow up, health education).

4. How does referral work here? Where are patients referred from? Where to? How frequently do you refer patients? After referral, do you maintain contact with the patient? How? Do you get feedback? What happens after discharge?

5. Please tell me what has worked well so far in: diagnosis of g CRD patients

6. Please tell me what has **not** worked well in diagnosis of CRD patients. (For each of the observations, why do you say so?)

7. Please tell me what has worked well so far in: treatment of CRD patients.

8. Please tell me what has **not** worked well in treatment of CRD patients. (For each of the observations, why do you say so?) Probe availability of drugs, timeliness of county governments, affordability of drugs, follow ups).

9. Did you receive any training specific to CRD patients? When was that? Do you have guidelines specific to CRD management? What are your experiences in implementing those guidelines?

10. What are your views of how services for CRD can be improved?

For peer review only

For peer review only

Appendix 4: In-depth interview guide for healthcare workers: Pharmacist

1. Please describe to me your roles in this department (how long have you been in this role? How big is your team? Please describe their roles?)

2. I am interested in understanding the process of treatment for patients who come with chronic respiratory problems. Please describe how these patients get services from the pharmacy. (Probe: prescriptions, drugs access).

3. Please tell me about the preferred drugs for CRD. Which of these do you stock? How do you procure these drugs? What are your experiences of availability of these drugs? Are there any that are covered for by NHIF?

4. Apart from drugs, do you procure any equipment used in the diagnosis of CRD e.g. peak flow meters?

5. Please tell me what has worked well so far in treating CRD patients.

6. Please tell me what has not worked well in treating CRD patients. *(Probe For each of the observations, why do you say so? availability of drugs, timeliness of county governments, affordability of drugs, follow ups).*

7. Did you receive any training on CRD management? (If yes: when?)

8. Do you have guidelines specific to CRD management? What are your experiences in implementing those guidelines?

9. What are your views of how services for CRD can be improved in this county?

For peer review only

Appendix 5: Interview guide for Community Health Extension Officer (CHEW)

1. Please tell me about your role as a CHEW.
2. Is chronic respiratory disease an issue in this CHU?
3. What is your involvement in CRD in your CHU?
4. Please describe how referrals for people with CRD to this facility works (probe: how patients are referred, any documentation from the CHV, records kept by CHEW).
5. How frequently are CRD patients referred? How does it work? What is your involvement?
6. After initial treatment, how do you follow up with patients' treatment? *(Probe: how do you get feedback about patients' condition, supporting follow up)*
7. Did you receive any specific training on CRD management? (If yes, when? Do you have any guidelines for supporting your community work? Have the CHV been trained in CRD case detection?)
8. Do you conduct any outreach services on CRD? How frequently do you do this? What is involved in the outreach?
9. Apart from county government, who are the other partners that you work with? (Probe: international organizations, local organizations)
10. What has worked well so far? Why do you say so?
11. What has not worked well so far? Why do you say so? What could be done differently to improve partnership between the health facility and community?

For peer review only

Appendix 6: In-depth Interview guide for Laboratory staff:

1. Please describe your roles in this department (how long have you been in this role? How big is your team? What does your day to day job involve?).

2. Please describe how patients with cough and/or breathlessness are referred to the lab? Please describe how sputum tests are done here? How frequently do you conduct these tests? Please describe the procedures involved in tests for people with cough and/or breathlessness?

3. In your experience, what are the major outcomes of the tests? How long does it take to get results? How are these results communicated to the patient? After lab, where does the patient go to?

4. Probe: what happens to patients with smear negative results for TB? Are other tests done? Are there patients who keep coming back to this facility? What do you do for them?

5. Please tell me what has worked well so far in testing people with chronic respiratory symptoms.

6. Please tell me what has not worked well in testing CRD patients. (Probe for each of the observations, why do you say so? availability of equipment, reagents).

7. Did you receive any training on CRD management? (If yes: when?)

8. Do you have guidelines specific to CRD management? What are your experiences in implementing those guidelines?

9. What are your views of how services for CRD can be improved in this county?

For peer review only

For peer review only

Appendix 2: Key informant interview guide for national-level Stakeholders-Kenya

1. Please describe to me your role in this organization. *Probe[How long have you been in this role? Which areas do you work within Kenya? Do you have other work outside the country?]*

2. Overall, how long have you been involved in NCD-related activities? Probe (what exactly drew you to NCD work?)

NCD management is gaining attention across the world. In which ways are you involved in making policies for NCD control in Kenya? *(Probe: is there a specific policy that you are particularly involved in? which one? How are you involved? Policy content, research, advocacy, financing, service provision)*

3.

4. Is chronic respiratory disease one of the NCDs you work with? (If yes – what do you mean by chronic respiratory disease? Please describe the work you do in relation to this? Whose responsibility is CRD in Kenya?

5. What do you think of the health policy on CRD in Kenya? (*Probe:* Is it appropriate? How well is it implemented? What are the key challenges in its implementation? What do you think could be done differently to make implementation better?).

6.

7. Please tell me who your partners are within Kenya. (Explain 'partners' to mean any organizations that you collaborate in any way on CRD work. Probe: national government, county governments, international organizations, CBO, politicians, media, any others?)

8. *If national/county governments are not mentioned in 4- what is your relationship with national/county government? How accessible are you to government decision makers? Are there counties that you partner with? Do you have representation in any of the government committees?*

9. How do you relate with the partners you have mentioned? *(Probe for joint policy development activities, representation in technical committees locally and internationally, advocacy, research, financing, political support).*

10. Among these partners, who do you think is the most powerful in influencing NCD policy development in Kenya? *(Probe: Why do you say so? Is there a partner that you feel should be included in NCD management efforts in Kenya? Why?)*

For peer review only

11. What has worked well in terms of patient care pathways? Why do you say so?

12. What has not worked well so far in terms of patient care pathways? *(Probe: Why do you say so? In your opinion, what could be done differently?)*

13. Over the next 5 years, what are your priorities in NCD control? What about for chronic respiratory disease? What opportunities do you see?

Thank the respondent and end the interview.

For peer review only

Appendix 2: Key informant interviews for policy-makers in national and county governments

**Institution/ Department**	
**Duration**	

1. Please describe to me your role in this department Probe[How long have you been in this role?]
2. Overall, how long have you been involved in NCD-related activities? *(Probe: what exactly drew you to NCD work?)*
3. NCD management is gaining attention across the world. In which ways are you involved in making policies for NCD control in Kenya? (Probe: is there a specific policy that you are particularly involved in? which one? How are you involved?)
4. Is chronic respiratory disease one of the NCDs you work with? (probes - If yes – what do you mean by chronic respiratory disease? Can you describe the work you do in relation to this? If no – can you share more about why not?)
5. Whose responsibility is CRD in Kenya?
6. Please tell me who your key partners are within Kenya. (Explain 'partners' to mean any organizations that you collaborate in any way on CRD work. Probe: national government, county governments, international organizations, CBO, politicians, media, any others?)
7. How do you relate with the partners you have mentioned? (Probe for joint policy development activities, representation in technical committees locally and internationally, advocacy, research, financing, political support).
8. Among these partners, who do you think is most influential CRD policy development in Kenya? Why do you say so? Is there a partner that you feel should be included in CRD management efforts in Kenya? Why?
9. (As appropriate) What is your relationship with national/county government in CRD management? How do you ensure that policy guidelines are adhered to? Do you offer any trainings?

For peer review only

10. What has worked well in terms of patient care pathways? Why do you say so?

11. What has not worked well in terms of patient care pathways so far? Why do you say so?

10. Over the next 5 years, what are your priorities in NCD control? What about for chronic respiratory disease? What opportunities do you see?

Thank the respondent and end the interview.

For peer review only

For peer review only

Appendix 3_Topic Guide- Malawi

Topic Guide (June 11th, 2018)

I really want to begin to understand diagnosis and treatment of chronic respiratory disease in the Malawi health system, and understand factors that have advanced and interfered with diagnosis and treatment

1. Tell me how your **job relates** to chronic respiratory disease (excluding TB). Probe: what is your experience with CRD

2. Tell me about medication supply and availability.
   Probe: what is your perspective on regulation, what is your experience and understanding of common obstacles that occur.
   Access to medication, constraints of:
   Market forces/transparency/donor agenda and funding

3. How do you see Malawian health system changing in recent years addressing chronic health conditions?
   Probe. Specifically CRD, what barriers seen in other countries experiencing.
   - Chronic Care clinic piloted in 2014 in area 25,
   - Package of essential NCD diagnosis and treatment in primary care was trailed in Kasungu District 2012, scale up 2015

4. How do you see clinicians using new Malawi **treatment guidelines** for Chronic respiratory disease diagnosis, and treatment? Spirometry/ Peak flow meter

5. What are some **problem-solving techniques** you use or see clinicians using to provide care to patients seeking medical care who do not have TB?

6. Can you tell me **influencers on actors and action** of NCD and CRD policy? Expand on system thinking in health decision making and policy design?
   Actors/context/process
   Policy communities/networks/venue
   Actors /institution that make decision

7. What do you consider to be the key issues that you see in Malawian health system that create barriers to better treatment of chronic respiratory disease?

   Tell me, have you heard about the Triage Project here in Malawi
   Probe: when did you become involved, what were some key events you saw happen, have you seen any predicted or unpredicted changes or challenges
   Anything else you would like to add?

For peer review only

For peer review only

For peer review only - http://bmjopen.bmj.com/site/about/guidelines.xhtml

Appendix 4_ Interview guides for Ugandan site

Appendix 7: Key informant interview guide for Stakeholders

1. Please describe your current role in this organization. Probe [How long have you been in this role? What other roles /positions related to non-communicable diseases do you hold?]

2. Overall, how long have you been involved in NCD-related activities? Probe [how did you come to be involved in NCD work?)

3. NCDs are recognised for being the biggest cause of disease worldwide and are steadily gaining attention in LMICs where they cause avertable premature deaths, what policies are in place for their management in Uganda? [what is the extent of attention received for NCDs in Uganda?]

4. What role do you play in making policies for NCD management in Uganda? [How involved are you in the policy making process? What specific policy stage where you most involved; content, research, advocacy, financing, service provision?]

5. In what ways do you think the policy for NCD management in Uganda is lacking? In which ways is it strong? [what changes would you make to the policy if you had to?]

6. How would you define Chronic lung diseases? In your opinion how big of a problem are CLDs in Uganda?

7. What specific policies are in place for CLD management in Uganda? [Apart from TB policy that is explicit and managed by the TB control program? What guides CLD management at health facilities in Uganda?]

8. Who are some of your partners within Uganda. ['Partners' here refers to any institutions, persons, organizations that you collaborate in any way on CLD work. Probe: national government partners, international organizations and donors, NGOs (international and local), community based organisations, politicians, media, any others? What roles do they mainly play?]

9. How do you relate with the partners you have mentioned? [Probe for joint policy development activities, representation in technical committees locally and internationally, advocacy, research, financing, political support].

10. Among these partners, who do you think are the most powerful in influencing NCD/CLD policy development in Uganda? Who of the partners have a high interest in CLD policy? [Probe: Why do you say so? Who are other partners that you feel should be included in NCD/CLD management efforts in Uganda? Why?]

11. What are the main sources of funding for NCD/CLD management in Uganda?

12. What in your opinion are barriers to implementation of CLD policy at the frontline/health facility level? [what could enhance the implementation of policies at the health facility level?]

13. Who is responsible for ensuring policies are adhered to? [Probes; What role do you have in ensuring that policy guidelines are adhered to? Do you offer any policy related training to frontline workers and implementers of the policies?]

14. What role can patients and their families play in the policy development process for NCD/CLD management in Uganda?

15. Over the next 5 years, what are your priorities in NCD/CLD management in Uganda?

For peer review only

Thank the respondent and end the interview.

Appendix 8: Key informant interviews for clinical services directors/facility in-charges at KCCA health facilities

1. Please describe your role (s) in this health facility? [Probes: How long have you held this role?]

2. How would you define chronic lung diseases?

3. I am interested in what policies are in place for the management of CLD in this facility and how management of CLDs happens in this facility. Do you have guidelines specific to CLD management? What are your experiences in implementing those guidelines? [Probes; what services for the diagnosis and management of CLD are available? Screening, diagnostics, treatment, follow up.]

4. What was the role your role in development of CLD management policy? What was your role in designing the implementation of the CLD policy? [Probes; if not involved who was? How does the implementation work?]

5. What has worked well so far in treating CLD patients in this facility? [Explain each of the things that have worked well, what has not worked well? Further probes; drugs and medications, patient literacy, patient education and family support?]

6. How can CLD services be improved in this facility?

7. What in your opinion can be done to improve the policies and their implementation? [Do you think you should be involved as a frontline manager in the development of CLD policy? Why? Who else should be involved?]

8. What role can patients play in the development of CLD policy and in the implementation of policy?

9. What in your opinion is the role of involving frontline health workers in developing policies?

10. What is the place of training on a policy at implementation level? [Probes; Were health workers here trained in CLD management? How often does such training occur and who organises the trainings?]

(Respondent may ask questions)

For peer review only

Appendix_5_Interview_guide healthcare workers_Sudan and Tanzania

IMPALA Phase1 Topic Guide Two_V2.0_23July2018

Interview Topic Guide for Health Workers (including patient vignettes)

Interview ID NO: _______________ Facilitator Initials: ___________ Note-taker Initials: _________

Type of Participant _________________ Audio file ID _________

Country/Health facility: ______________________ Date of Interview ____________________

Where do you work? ______________________________

What is your job title? ____

How long have you worked as (job title)? __________

Current Disease Priorities within the Health Facility

Can you tell me more about the services that you provide at this health facility?

What are the main priority diseases and illnesses for this facility?

Why are these the main priorities?

Can you tell me about the context of diseases such as Asthma, COPD and TB at this facility?

Readiness for integration of CLD services

Can you tell me more about the services you provide for patients with TB and other lung diseases at this facility?

- Prompt specifically for: Asthma, COPD, occupational lung disease
- How does this link to the community health system? e.g. community health workers, informal health providers etc.
- Please tell me more about how CLDs are diagnosed?
- Please tell me more about how CLDs are managed?

How ready do you feel your health facility is to be able to properly manage and integrate CLDs into routine service/programme delivery?

- ***Case Detection:***
  i. What equipment do you feel is needed to diagnose and manage patients with CLDs?
  ii. What are the challenges with ensuring this equipment if available, functional and used within your facility? What helps?

For peer review only

iii. Do you have a standard case definition for any chronic lung diseases (asthma, COPD etc) to guide you in case detection? (if yes, get some details and request a copy).

iv. Based on your experience in this facility can you comment on how long it takes to detect CLD? (probe for early/late detection, severity of cases when received)

- ***Standardised treatment and effective drug supply, including medical products and technology:***

i. Are there algorithms for staff in your facility to follow to diagnose and manage patients with CLDs? If yes, who was involved with developing these? Who showed you how to use them? What do you think about these algorithms?

ii. Are drugs for managing CLDs included in essential drug lists for your facility? Why? Why not? How available are these medicines? How often do you receive medicines? How much do they cost?

- ***Service delivery***

i. Can you tell me more about the services currently available for patients with TB and other chronic lung diseases (e.g. asthma and COPD) at your facility?

ii. How do you think the quality of these services could be improved?

iii. Based on your experiences, what do you think effective CLD care looks like?

iv. What do you think are the main needs for patients with CLDs?

v. How prepared is your facility to meet these needs? What changes need to be made to meet these needs?

b. How do you think services for CLD could be integrated with other services? (e.g. TB/NCDs etc).

- ***Health workforce***

i. Who do you think should be involved in the management of CLD within your facility? What should their roles and responsibilities be?

ii. Are there adequate numbers of staff to manage CLDs in your facility? Why? Why not?

iii. How are staff currently trained to manage CLDs? (pre-service training, in-service training?) How does this vary depending on job role? How could this be improved?

iv. What supervision structures are in place for health staff involved in the management of CLD within this facility? What works well about supervision? What could be improved?

v. What is needed to ensure staff are ready to diagnose and manage patients with CLDs?

- ***Reporting, recording of CLD cases and analysing CLD data for action***

i. What data is currently captured in relation to CLD in existing health information management systems?

ii. Where is CLD data reported? And how often? (probe for the flow of information)

For peer review only

iii. What analysis is done regarding CLDs? How is this information used?

What are the main barriers to diagnosing and managing patients with CLDs?

- How can these barriers be overcome?

What are the things that help with diagnosis and management currently?

What would make it easier to diagnose and manage patients with CLDs?

What do you think would be the main benefits of integrating the management of CLDs within existing health programmes?

- Which programmes or services would allow for integration?

What do you think would be the main challenges of integrating the management of CLDs within existing health programmes?

- How can these potential threats be avoided?

For peer review only

The following type of patient vignettes will also be used with health workers to assess their current skills for CLD management. It will be made clear to the participants that this is not a test and the results of the study will not be shared with supervisors/colleagues etc.

EXERCISES – Module 9

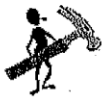

Revise what you have learnt and practise filling in the Asthma Treatment Card and Quarterly Reports.

The following patients have arrived with respiratory symptoms and are suspected cases of asthma. Answer the questions. If it is a case of persistent asthma, record the information on the Asthma Treatment Card.

Patient 1

Man, 60 years old. Retired from work in textile industry.

Clinical signs:

- episodes of coughing with breathlessness and sputum expectoration for the last 3 weeks
- has increased purulence of sputum for the last 5 days
- traces of blood in expectoration once or twice
- temperature has been 38 degrees for the last 3 days

History:

- episodes of coughing in the morning for the last 5 years
- breathlessness after effort for the last year
- temperature has been normal, but 5 kg weight loss over the last year
- no history of allergic disease or tuberculosis
- no known co-morbidity
- Smoking: ex-smoker (smoked 30 cig/day over period of more than 20 years; stopped smoking 3 years ago)

Clinical examination:

- Normal apart from some ronchi and wheezing.

1. What is or what are your suspected diagnoses?…………………………………

2. What do you decide to do? What do you prescribe?

……………………………………………………………………………………

……………………………………………………………………………………

……………………………………………………………………………………

After 8 days, patient improves: coughing, expectoration and breathlessness decrease. Weight is stable. Bacteriological examinations are negative, in particular AFB smear.
Predicted PEF: 536 l/min
PEF measurements are as follows:
PEF before bronchodilator = 280 l/min
PEF after bronchodilator = 290 l/min
However, as the breathlessness has not completely disappeared, the doctor prescribes a short course of prednisone, with salbutamol as needed. After this treatment, the patient comes back and the PEF is measured:
PEF after bronchodilator after 8 days of prednisone = 300 l/min

3. What is the patient's PEF variability?

……………………………………………………………………………………

……………………………………………………………………………………

4. What is the most probable diagnosis?

……………………………………………………………………………………

5. What is the severity of the disease?

……………………………………………………………………………………

……………………………………………………………………………………

If your diagnosis is asthma, complete an Asthma Treatment Card.

6. Teach/Check patient inhaler technique

7. Teach/Check patient ability to self-control attacks
8. How would you manage the patient if he had an acute asthma attack?
9. Please describe how you would respond if the patient told you he was no longer able to carry out his work, due to his breathlessness and he was feeling quite low as a result
    a. What else might you want to consider when managing this patient given his recent low mood?
    b. Are there any other service sectors that you might want to link this patient with?

For peer review only

APPENDIX 6_KEY INFORMANT INTERVIEWS WITH STAKEHOLDERS_SUDAN AND TANZANIA

IMPALA Phase1 Topic Guide Two_V2.0_23July2018

Interview Topic Guide for Health Workers (including patient vignettes)

Interview ID NO: _______________ Facilitator Initials: ____________ Note-taker Initials: _________	
Type of Participant _________________ Audio file ID __________	
Country/Health facility: _______________________	Date of Interview _____________________
Where do you work? ______________________________	
What is your job title? ____	
How long have you worked as (job title)? ___________	

Current Disease Priorities within the Health Facility

Can you tell me more about the services that you provide at this health facility?

What are the main priority diseases and illnesses for this facility?

Why are these the main priorities?

Can you tell me about the context of diseases such as Asthma, COPD and TB at this facility?

Readiness for integration of CLD services

Can you tell me more about the services you provide for patients with TB and other lung diseases at this facility?

- Prompt specifically for: Asthma, COPD, occupational lung disease
- How does this link to the community health system? e.g. community health workers, informal health providers etc.
- Please tell me more about how CLDs are diagnosed?
- Please tell me more about how CLDs are managed?

How ready do you feel your health facility is to be able to properly manage and integrate CLDs into routine service/programme delivery?

- ***Case Detection:***
    i. What equipment do you feel is needed to diagnose and manage patients with CLDs?
    ii. What are the challenges with ensuring this equipment if available, functional and used within your facility? What helps?

For peer review only

iii. Do you have a standard case definition for any chronic lung diseases (asthma, COPD etc) to guide you in case detection? (if yes, get some details and request a copy).

iv. Based on your experience in this facility can you comment on how long it takes to detect CLD? (probe for early/late detection, severity of cases when received)

- ***Standardised treatment and effective drug supply, including medical products and technology:***

i. Are there algorithms for staff in your facility to follow to diagnose and manage patients with CLDs? If yes, who was involved with developing these? Who showed you how to use them? What do you think about these algorithms?

ii. Are drugs for managing CLDs included in essential drug lists for your facility? Why? Why not? How available are these medicines? How often do you receive medicines? How much do they cost?

- ***Service delivery***

i. Can you tell me more about the services currently available for patients with TB and other chronic lung diseases (e.g. asthma and COPD) at your facility?

ii. How do you think the quality of these services could be improved?

iii. Based on your experiences, what do you think effective CLD care looks like?

iv. What do you think are the main needs for patients with CLDs?

v. How prepared is your facility to meet these needs? What changes need to be made to meet these needs?

b. How do you think services for CLD could be integrated with other services? (e.g. TB/NCDs etc).

- ***Health workforce***

i. Who do you think should be involved in the management of CLD within your facility? What should their roles and responsibilities be?

ii. Are there adequate numbers of staff to manage CLDs in your facility? Why? Why not?

iii. How are staff currently trained to manage CLDs? (pre-service training, in-service training?) How does this vary depending on job role? How could this be improved?

iv. What supervision structures are in place for health staff involved in the management of CLD within this facility? What works well about supervision? What could be improved?

v. What is needed to ensure staff are ready to diagnose and manage patients with CLDs?

- ***Reporting, recording of CLD cases and analysing CLD data for action***

i. What data is currently captured in relation to CLD in existing health information management systems?

ii. Where is CLD data reported? And how often? (probe for the flow of information)

For peer review only

iii. What analysis is done regarding CLDs? How is this information used?

What are the main barriers to diagnosing and managing patients with CLDs?

- How can these barriers be overcome?

What are the things that help with diagnosis and management currently?

What would make it easier to diagnose and manage patients with CLDs?

What do you think would be the main benefits of integrating the management of CLDs within existing health programmes?

- Which programmes or services would allow for integration?

What do you think would be the main challenges of integrating the management of CLDs within existing health programmes?

- How can these potential threats be avoided?

For peer review only

The following type of patient vignettes will also be used with health workers to assess their current skills for CLD management. It will be made clear to the participants that this is not a test and the results of the study will not be shared with

EXERCISES – Module 9

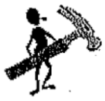

Revise what you have learnt and practise filling in the Asthma Treatment Card and Quarterly Reports.

The following patients have arrived with respiratory symptoms and are suspected cases of asthma. Answer the questions. If it is a case of persistent asthma, record the information on the Asthma Treatment Card.

Patient 1
Man, 60 years old. Retired from work in textile industry.
Clinical signs:
- episodes of coughing with breathlessness and sputum expectoration for the last 3 weeks
- has increased purulence of sputum for the last 5 days
- traces of blood in expectoration once or twice
- temperature has been 38 degrees for the last 3 days

History:
- episodes of coughing in the morning for the last 5 years
- breathlessness after effort for the last year
- temperature has been normal, but 5 kg weight loss over the last year
- no history of allergic disease or tuberculosis
- no known co-morbidity
- Smoking: ex-smoker (smoked 30 cig/day over period of more than 20 years; stopped smoking 3 years ago)

Clinical examination:
- Normal apart from some ronchi and wheezing.

1. What is or what are your suspected diagnoses?…………………………………

2. What do you decide to do? What do you prescribe?
…………………………………………………………………………………………
…………………………………………………………………………………………
…………………………………………………………………………………………

After 8 days, patient improves: coughing, expectoration and breathlessness decrease. Weight is stable. Bacteriological examinations are negative, in particular AFB smear.
Predicted PEF: 536 l/min
PEF measurements are as follows:
PEF before bronchodilator = 280 l/min
PEF after bronchodilator = 290 l/min
However, as the breathlessness has not completely disappeared, the doctor prescribes a short course of prednisone, with salbutamol as needed. After this treatment, the patient comes back and the PEF is measured:
PEF after bronchodilator after 8 days of prednisone = 300 l/min

supervisors/colleagues etc.

3. What is the patient's PEF variability?
…………………………………………………………………………………………
…………………………………………………………………………………………

4. What is the most probable diagnosis?
…………………………………………………………………………………………

5. What is the severity of the disease?
…………………………………………………………………………………………
…………………………………………………………………………………………

If your diagnosis is asthma, complete an Asthma Treatment Card.

6. Teach/Check patient inhaler technique

7. Teach/Check patient ability to self-control attacks
8. How would you manage the patient if he had an acute asthma attack?
9. Please describe how you would respond if the patient told you he was no longer able to carry out his work, due to his breathlessness and he was feeling quite low as a result
    a. What else might you want to consider when managing this patient given his recent low mood?
    b. Are there any other service sectors that you might want to link this patient with?

For peer review only

Standards for Reporting Qualitative Research (SRQR)

O'Brien B.C., Harris, I.B., Beckman, T.J., Reed, D.A., & Cook, D.A. (2014). Standards for reporting qualitative research: a synthesis of recommendations. *Academic Medicine, 89(9)*, 1245-1251.

No. Topic	Item	Page
**Title and abstract**		
S1 Title	Concise description of the nature and topic of the study identifying the study as qualitative or indicating the approach (e.g., ethnography, grounded theory) or data collection methods (e.g., interview, focus group) is recommended	Title page
S2 Abstract	Summary of key elements of the study using the abstract format of the intended publication; typically includes objective, methods, results, and conclusions	Please refer to abstract page-2
**Introduction**		
S3 Problem formulation	Description and significance of the problem/phenomenon studied; review of relevant theory and empirical work; problem statement	Please refer to Page 3
S4 Purpose or research question	Purpose of the study and specific objectives or questions	Page 3
**Methods**		
S5 Qualitative approach and research paradigm	Qualitative approach (e.g., ethnography, grounded theory, case study, phenomenology, narrative research) and guiding theory if appropriate; identifying the research paradigm (e.g., positivist, constructivist/interpretivist) is also recommended	
S6 Researcher characteristics and reflexivity	Researchers' characteristics that may influence the research, including personal attributes, qualifications/experience, relationship with participants, assumptions, or presuppositions; potential or actual interaction between researchers' characteristics and the research questions, approach, methods, results, or transferability	Please refer to last sentence in the data collection section:<br><br>"Interviews were conducted by experienced qualitative researchers" pp4
S7 Context	Setting/site and salient contextual factors; rationale[a]	Context is described in 2nd paragraph of the

For peer review only

		introduction- pp3
S8 Sampling strategy	How and why research participants, documents, or events were selected; criteria for deciding when no further sampling was necessary (e.g., sampling saturation); rationale[a]	Methods section pp3. Healthcare workers and policy makers sampled for their roles in management of chronic lung diseases and policy making, respectively.
S9 Ethical issues pertaining to human subjects	Documentation of approval by an appropriate ethics review board and participant consent, or explanation for lack thereof; other confidentiality and data security issues	"Ethics" section, pp4.
S10 Data collection methods	Types of data collected; details of data collection procedures including (as appropriate) start and stop dates of data collection and analysis, iterative process, triangulation of sources/methods, and modification of procedures in response to evolving study findings; rationale[a]	Page 4, data collection section
S11 Data collection instruments and technologies	Description of instruments (e.g., interview guides, questionnaires) and devices (e.g., audio recorders) used for data collection; if/how the instrument(s) changed over the course of the study	Data collection, page 4
S12 Units of study	Number and relevant characteristics of participants, documents, or events included in the study; level of participation (could be reported in results)	Table 1 and 2
S13 Data processing	Methods for processing data prior to and during analysis, including transcription, data entry, data management and security, verification of data integrity, data coding, and anonymization/deidentification of excerpts	Intercountry analysis, page 4
S14 Data analysis	Process by which inferences, themes, etc., were identified and developed, including researchers involved in data analysis; usually references a specific paradigm or approach; rationale[a]	Please refer to the Intercountry analysis pp4, researcher roles listed under 'Author contributions' page 10
S15 Techniques to enhance trustworthiness	Techniques to enhance trustworthiness and credibility of data analysis (e.g., member checking, audit trail, triangulation); rationale[a]	Please refer to the Data collection section- verbatim transcripts (page 4); excerpts from transcripts used to illustrate specific findings (page 5)
**Results/Findings**		
S16 Synthesis and interpretation	Main findings (e.g., interpretations, inferences, and themes); might include development of a theory or model, or integration with prior research or theory	Findings section- data analysis uses the WHO health systems building blocks
S17 Links to empirical data	Evidence (e.g., quotes, field notes, text excerpts, photographs) to substantiate analytic findings	Verbatim quotes used in the Findings section

Discussion		
S18 Integration with prior work, implications, transferability, and contribution(s) to the field	Short summary of main findings; explanation of how findings and conclusions connect to, support, elaborate on, or challenge conclusions of earlier scholarship; discussion of scope of application/generalizability; identification of unique contribution(s) to scholarship in a discipline or field	Please refer to discussion section, pp 8
S19 Limitations	Trustworthiness and limitations of findings	Please refer to limitations section, page 9
**Other**		
S20 Conflicts of interest	Potential sources of influence or perceived influence on study conduct and conclusions; how these were managed	None declared
S21 Funding	Sources of funding and other support; role of funders in data collection, interpretation, and reporting	Role of funding source, page 4

[a]The rationale should briefly discuss the justification for choosing that theory, approach, method, or technique rather than other options available, the assumptions and limitations implicit in those choices, and how those choices influence study conclusions and transferability. As appropriate, the rationale for several items might be discussed together.

For peer review only

What are the barriers to the diagnosis and management of chronic respiratory disease in sub-Saharan Africa? a qualitative study with healthcare workers, national and regional policy stakeholders in five countries

Journal:	*BMJ Open*
Manuscript ID	bmjopen-2021-052105.R2
Article Type:	Original research
Date Submitted by the Author:	09-Jun-2022
Complete List of Authors:	MULUPI, STEPHEN; Liverpool School of Tropical Medicine, International Public Health ; KEMRI, Centre for Respiratory Diseases Research<br>Ayakaka, Irene; Liverpool School of Tropical Medicine, International Public Health; Makerere University, Lung Institute<br>Tolhurst, Rachel; Liverpool School of Tropical Medicine, International Public Health<br>Kozak, Nicole; Liverpool School of Tropical Medicine, International Public Health ; REACH Trust Malawi<br>Shayo, Elizabeth; Liverpool School of Tropical Medicine, International Public Health ; National Institute of Medical Research, Tanzania<br>Abdalla, Elhafiz; Epi-Lab<br>Osman, Rashid; Epi-Lab<br>Egere, Uzochukwu; Liverpool School of Tropical Medicine<br>Mpagama, Stellah; 4. Kibong'oto Infectious Diseases Hospital/Kilimanjaro Christian Medical University<br>Chinouya, Martha; Liverpool School of Tropical Medicine, Department of International Public Health<br>Chikaphupha, Kingsley; REACH Trust, Malawi<br>Elsony, Asma; Epi-Lab<br>Meme, Helen; Kenya Medical Research Institute (KEMRI), Centre for Respiratory Diseases Research<br>Oronje, Rose; African Institute for Development Policy (AFIDEP)<br>Ntinginya, Nyanda; National Institute for Medical Research (NIMR)-Mbeya Medical Research Centre ,<br>Obasi, Angela; Liverpool School of Tropical Medicine, International Public Health ; Liverpool University Hospitals NHS Foundation Trust<br>Taegtmeyer, Miriam; Liverpool School of Tropical Medicine, International Public Health ; Liverpool University Hospitals NHS Foundation Trust, Tropical Infectious Diseases Unit
**Primary Subject Heading**:	Health services research
Secondary Subject Heading:	Health policy
Keywords:	Asthma < THORACIC MEDICINE, Health policy < HEALTH SERVICES ADMINISTRATION & MANAGEMENT, Protocols & guidelines < HEALTH SERVICES ADMINISTRATION & MANAGEMENT, EDUCATION & TRAINING

	(see Medical Education & Training), PRIMARY CARE, Chronic airways disease < THORACIC MEDICINE

SCHOLARONE™
Manuscripts

I, the Submitting Author has the right to grant and does grant on behalf of all authors of the Work (as defined in the below author licence), an exclusive licence and/or a non-exclusive licence for contributions from authors who are: i) UK Crown employees; ii) where BMJ has agreed a CC-BY licence shall apply, and/or iii) in accordance with the terms applicable for US Federal Government officers or employees acting as part of their official duties; on a worldwide, perpetual, irrevocable, royalty-free basis to BMJ Publishing Group Ltd ("BMJ") its licensees and where the relevant Journal is co-owned by BMJ to the co-owners of the Journal, to publish the Work in this journal and any other BMJ products and to exploit all rights, as set out in our licence.

The Submitting Author accepts and understands that any supply made under these terms is made by BMJ to the Submitting Author unless you are acting as an employee on behalf of your employer or a postgraduate student of an affiliated institution which is paying any applicable article publishing charge ("APC") for Open Access articles. Where the Submitting Author wishes to make the Work available on an Open Access basis (and intends to pay the relevant APC), the terms of reuse of such Open Access shall be governed by a Creative Commons licence – details of these licences and which Creative Commons licence will apply to this Work are set out in our licence referred to above.

Other than as permitted in any relevant BMJ Author's Self Archiving Policies, I confirm this Work has not been accepted for publication elsewhere, is not being considered for publication elsewhere and does not duplicate material already published. I confirm all authors consent to publication of this Work and authorise the granting of this licence.

For peer review only

What are the barriers to the diagnosis and management of chronic respiratory disease in sub-Saharan Africa? a qualitative study with healthcare workers, national and regional policy stakeholders in five countries

Authors: Stephen Mulupi*[1,2], Irene Ayakaka*[1,3], Rachel Tolhurst[1], Nicole Kozak[1,4], Elizabeth Shayo[1,5], Elhafiz Abdalla[6], Rashid Osman[6], Uzochukwu Egere[1,] Stellah Mpagama[7], Martha J. Chinouya[1], Kingsley Chikaphupha[4], Asma El Sony[6], Hellen Meme[2], Rose Oronje[8], Nyanda Elias Ntinginya[5], Angela I. Obasi[1,9], Miriam Taegtmeyer[1,10]

*Joint first authors

Corresponding author: Stephen Mulupi

E-mail: stephen.mulupi@lstmed.ac.uk

Mobile phone number +254720640330

Affiliations:

1. Liverpool School of Tropical Medicine, Department of International Public Health, Liverpool, UK
2. Kenya Medical Research Institute, Centre for Respiratory Diseases Research (KEMRI-CRDR)
3. Makerere University Lung institute, (MLI)
4. REACH Trust, Malawi
5. National Institute for Medical Research, Tanzania
6. EpiLab, Sudan
7. Kibong'oto Infectious Disease Hospital, Tanzania
8. African Institute for Development Policy (AFIDEP)
9. AXESS Sexual Health, Liverpool University Hospitals NHS Foundation Trust, Liverpool, UK
10. Tropical Infectious Diseases Unit, Liverpool University Hospitals NHS Foundation Trust, Liverpool, UK

Contact information for authors:

1. Stephen Mulupi: stephen.mulupi@lstmed.ac.uk
2. Irene Ayakaka: irene.ayakaka@lstmed.ac.uk
3. Rachel Tolhurst: rachel.tolhurst@lstmed.ac.uk
4. Nicole Kozak: molemoe@gmail.com
5. Elizabeth Shayo: bshayo@yahoo.com
6. Elhafiz Abdalla: hafizhussein@gmail.com
7. Rashid Osman: rashildo@hotmail.com
8. Uzochukwu Egere: uzochukwu.egere@lstmed.ac.uk
9. Stellah Mpagama: sempagama@yahoo.com
10. Martha J. Chinouya: martha.chinouya@lstmed.ac.uk
11. Kingsley Chikaphupha: kchikaphupha@gmail.com.
12. Asma El Sony: asmaelsony@gmail.com
13. Hellen Meme: helenmeme@gmail.com
14. Rose Oronje: rose.oronje@afidep.org
15. Nyanda Elias Ntinginya: nelias@nimr-mmrc.org
16. Angela I. Obasi: Angela.Obasi@lstmed.ac.uk
17. Miriam Taegtmeyer: miriam.taegtmeyer@lstmed.ac.uk

On behalf of The NIHR International Multidisciplinary Programme to Address Lung Health and TB in Africa (IMPALA) Consortium: Emmanuel Addo-Yobo, Brian Allwood, Hastings Banda, Imelda Bates, Amsalu Binegdie, Adegoke Falade, Jahangir Khan, Maia Lesosky, Bertrand Mbatchou, Kevin Mortimer, Beatrice Mutayoba, Louis Niessen, Jamie Rylance, S Bertel Squire, William Worodria, Heather Zar, Eliya Zulu, Lindsay Zurba

For peer review only - http://bmjopen.bmj.com/site/about/guidelines.xhtml

ABSTRACT

Objectives Chronic respiratory diseases (CRDs) are among the top four non-communicable diseases globally. They are associated with poor health and approximately 4 million deaths every year. The rising burden of chronic respiratory diseases in low-and-middle-income countries will strain already weak health systems. This study aimed to explore the perspectives of healthcare workers and other health policy stakeholders on the barriers to effective diagnosis and management chronic respiratory diseases in Kenya, Malawi, Sudan, Tanzania, and Uganda.

Study design: qualitative descriptive study

Settings Primary, secondary, and tertiary health facilities, government agencies, and civil society organisations in 5 sub-Saharan African countries.

Participants We purposively selected 60 national, and district level policy stakeholders, and 49 healthcare workers, based on their roles in policy decision-making or health provision and conducted key-informant interviews and in-depth interviews respectively, between 2018 and 2019. Data were analysed through framework approach.

Results

We identified intersecting vicious cycles of neglect of chronic respiratory diseases at strategic policy and healthcare facility levels. Lack of reliable data on burden of disease, due to weak information systems and diagnostic capacity, negatively affected inclusion in policy, this in turn was reflected by low budgetary allocations for diagnostic equipment, training, and medicines. At the healthcare facility level, inadequate budgetary allocations constrained diagnostic capacity, quality of service delivery and collection of appropriate data, compounding the lack of routine data on burden of disease.

Conclusion

Health systems in the five countries are ill-equipped to respond to chronic respiratory diseases, an issue that has been brought into sharp focus as countries plan for post-COVID-19 lung diseases. Chronic respiratory diseases are under-diagnosed, under-reported and underfunded leading to a vicious cycle of invisibility and neglect. Appropriate diagnosis and management require health systems strengthening, particularly at the primary healthcare level.

Strengths and limitations

- Strengths and limitations of this study

  Strengths
- The five studies were conducted independently by the country study teams, allowing them to take local health system factors in each country into account when interviewing
- The inclusion of five countries with very similar emerging themes allows for comparison between countries and gives a sense of how widespread barriers to CRD are.
- We were able to triangulate perspectives of different types of study participants, enhancing rigour in our analysis.

  Limitations
- Differences in sampling the study sites e.g., number and levels of hospitals and rural/ urban variations limited comparisons.
- We were not able to patients' perspectives in demanding and accessing health services for CRDs.

For peer review only - http://bmjopen.bmj.com/site/about/guidelines.xhtml

1. Introduction

Chronic respiratory disease (CRD) accounts for approximately 4 million deaths per year world-wide[1, 2]. Globally the most prevalent CRDs are asthma and chronic obstructive pulmonary disease (COPD) [3] but the definition includes other non-infectious lung and airway disease such as bronchiectasis and post-TB lung disease. The burden of CRD morbidity and mortality is rising steeply in low- and middle-income countries (LMICs)[4, 5]. The high prevalence of CRD in sub-Saharan Africa (SSA) is driven in part by high rates of recurrent childhood respiratory infections and pulmonary TB which are important precursors of CRD[6-9]. A hospital-based study in Tanzania among post-TB patients estimated 74% prevalence of abnormal lung function[10]. Other common risk factors include exposure to tobacco smoke, indoor pollution from biomass fuels and occupational exposures in the mining industry, sugar, and tobacco plantations[11, 12]. In Uganda, the age-adjusted prevalence for any chronic respiratory condition in both rural and urban settings is 20% [13] and in Malawi over 40% of adults randomly sampled in an urban population had abnormal lung function[14].

The management of CRD in SSA is undermined by weak health systems characterised by lack of appropriate medical equipment and low diagnostic capacity[15, 16]. Healthcare workers trained in the management of CRD are scarce and medicines are either unavailable or unaffordable by many patients[17, 18]. Existing lung health pathways in SSA focus on TB with smear negative patients repeatedly visiting health facilities or being referred from one facility to another seeking definitive diagnosis. A wide range of policy design, implementation and service delivery gaps undermine CRD care pathways. Importantly, there is limited understanding in how specific policy and health facility level factors interact and shape health service delivery in SSA contexts.

In this qualitative study, we sought to understand and characterize the views and perspectives of policymakers and healthcare workers on CRD policies and services in five countries in SSA. We present the findings that show clear linkage of policy-level factors to service delivery experiences. We contribute evidence to inform the design of future policy interventions to strengthen the delivery of services and ultimately improve CRD management in similar settings. The five linked studies formed baseline assessments for country-level research within the National Institute for Health Research (NIHR), International Multidisciplinary Programme to Address Lung Health and TB in Africa (IMPALA[1]).

Methods

Five studies were independently conducted in Kenya, Malawi, Sudan, Tanzania, and Uganda between 2018 and 2019 based on a constructivist philosophical paradigm using qualitative study methods[19, 20] to collect data and to perform data validation, analysis and interpretation to answer the study questions. Ministry of Health officials and other healthcare policy stakeholders were purposively sampled for their roles and experiences in policy design and implementation alongside health workers experienced in the management of CRD (Table 1).To secure interview appointments participants were contacted through e-mail, phone or verbally, as appropriate. Public healthcare facilities were selected to include both urban

[1] https://www.lstmed.ac.uk/impala

For peer review only - http://bmjopen.bmj.com/site/about/guidelines.xhtml

and rural settings and different facility levels (Table 2). All served populations with high prevalence rates of TB and other lung conditions.

For peer review only

For peer review only - http://bmjopen.bmj.com/site/about/guidelines.xhtml

Table 1: The distribution of participants across the five case study countries.

**Informants**	**Kenya**	**Malawi**	**Sudan**	**Tanzania**	**Uganda**
National, regional and district level policy stakeholders	15	13	14	13	5
Health care workers	14	5*	14	10	6
**Total**	**29**	**18**	**28**	**23**	**11**

***Senior clinicians**

Table 2: Distribution of healthcare facilities in the five case study countries

**Public health system level**	**Kenya**	**Malawi**	**Sudan**	**Tanzania**	**Uganda**	**Total**
Primary healthcare facilities (health centre and dispensary level)	3	1	2	8	6	20
District level hospitals	1	1	8	1	0	11
National/regional referral hospitals	0	2	0	1	2	5
**Total number of facilities**	**4**	**4**	**10**	**10**	**8**	**36**

Role of the funding source

This research was funded by the (NIHR IMPALA, grant reference 16/136/35) using UK aid from the UK Government to support global health research. The views expressed in this publication are those of the author(s) and not necessarily those of the NIHR or the UK Department of Health and Social Care.

Data collection

Key informant interviews (KIIs) with policy stakeholders (appendix 1-3) explored opinions about prioritization of CRD; (un)availability and operationalization of CRD policies; systemic factors enhancing or impeding provision of healthcare services for CRD; and opinions on how systems could be improved. In-depth interviews with healthcare workers (appendix 4-6), explored their experiences in diagnosing and managing CRD; availability of diagnostic equipment and medicines; experience of training; and their perceptions of what has worked well or not, in management of CRD. Semi-structured interview guides were developed broadly aligning with the health systems' building blocks[21]. Interviews were conducted May 2018 to March 2019, by experienced qualitative researchers in English, or the local languages as appropriate, and digitally recorded with consent. These face-to-face interviews took place in quiet, private rooms within the workplaces of the participants, to protect participants' confidentiality, minimize disruptions and ensure quality of audio recordings. The discussions took around 40 minutes. The researchers in Sudan, Malawi and Tanzania, had no prior relationships with the participants. In Malawi a female white Canadian researcher (NK) conducted some of the interviews (others were done by a Chichewa speaking research assistant) and came from an outsider perspective. The researcher from Uganda (IA) is a respected female medical doctor who was known to some of the participants. This positionality required critical reflection on being an "insider" to the health system during the research period. Similarly in Kenya, affiliation of the male researcher (SM) to the Kenya Medical Research Institute, a government agency, may have shaped trust and response to the study, though the effect on issues is unknown. The researchers jotted notes about participants' comments and the researcher's own thoughts during the interviews; they used memoing as soon as possible after an

For peer review only - http://bmjopen.bmj.com/site/about/guidelines.xhtml

interview and during the transcription of recordings. Recordings were transcribed verbatim and translated to English for analysis. Field notes complemented the audio recordings.

Intercountry analysis.

Data collection was preceded by a common training in policy (2018) for the researchers and followed by joint analysis meetings that discussed similarities and differences between country-specific study findings; the codes and topics were identified within each country and combined during analysis and the respective country teams re-read transcripts to confirm accuracy and identify emerging themes. Authors SM, IA, RT, MT, ES, participated in the coding meetings and determination of the final codebook. Initial codebooks were developed from the broad interview guide topics, then updated inductively as novel codes emerged. A final codebook was discussed, further refined, and applied to the data, using the framework approach [22], with analysis supported by NVivo™ v11.0 (QSR International 1999) software. The main themes and subthemes were organised through NVivo nodes and sub nodes, respectively, and subsequently into charts. An exploration of emerging patterns led to the identification of final themes with selected quotes used to illustrate specific findings.

Ethics

The Liverpool School of Tropical Medicine Ethics Committee approved these studies separately (Kenya: protocol 18-054; Uganda: protocol 18-037; Malawi: protocol M1803; Tanzania and Sudan; protocol 18-043). Additionally, each approval was by in-country committees[2]. All participants gave written, informed consent.

Findings

The World Health Organization (WHO), in 2007 proposed a framework describing health system functions in terms of six core interdependent components or "building blocks": (i) service delivery; (ii) health workforce; (iii) health information systems; (iv) access to essential medicines; (v) financing; and (vi) leadership/governance[21]. Effective coordination, and optimal performance of these health system blocks would theoretically enhance achievement of health system goals, i.e. "improving health and health equity, in ways that are responsive, financially fair, and make the best, or most efficient, use of available resources"[21]. We used the WHO health systems building blocks to frame our exploration at strategic and service delivery levels. A picture emerged of neglect of CRD at each level of the systems and for each building block with consequences across the whole system.

<u>Strategic level</u>

Variable availability and awareness of policy

CRD policy availability varied between countries. Kenya and Tanzania have fully developed and adopted policy strategies for lung health[23, 24] and the national and district stakeholders were aware of this; however, few health care workers were aware of the policies or where to find them. There was no stand-alone policy for CRD in Uganda and in Malawi; CRD was embedded within NCD policy, which was in draft, with limited awareness among all levels of participants. In Sudan, there was no specific policy for CRD.

> *"I do not know whether there is an existing policy for TB and CRD. There is a general strategy; the national health policy that generally addresses chronic diseases." (KII-F, Sudan Federal-MoH official)*

---

[2] Kenya Medical Research Institute (KEMRI/SERU/CRDR/037/3717); Malawi National Health Science Research committee (Protocol # 18/04/2021); Sudan: Ref 44/T/Kh/1; Tanzania: Medical Research Coordinating Committee of the National Institute for Medical Research, (NIMR/HQ/R.8a/V.IX/2922) and Uganda (TASO) IRB:TASOREC /030/18-UG-REC-009 and the Uganda National Council for Science and Technology HS232ES).

For peer review only - http://bmjopen.bmj.com/site/about/guidelines.xhtml

Lack of CRD data

In all five contexts, policy stakeholders associated the limited data on burden of disease with low visibility and low domestic budget allocations for CRD as shown by this typical quote:

> *"For COPD we don't have data, we don't know its prevalence, we don't know its incidence, we don't know its mortality" (IDI national informant, Tanzania).*

None of the study countries had comprehensive population surveys on CRD, but Sudan, Kenya and Uganda reported research evidence about asthma, COPD and the growing burden of CRD. In Malawi, participants noted that CRD was not included in the recent nationwide burden of disease study and went further to highlight the lack of data on cost-effectiveness.

> *" So [CRDs] never feature in the cost effectiveness analyses that allow for their prioritization, benchmarked against the other priorities where there is evidence….for chronic respiratory disease we have to recognize that the evidence base for the country is very tiny. So being able to advocate beyond, the NCD Department into the Treasury, into the Health Sector Strategic Plan, is multiple steps away" (Malawi-KI 18, researcher).*

Neither was routine data able to fill the evidence gaps due to a lack of appropriate data collection tools and shortages of health information and records officers. Participants in all the five countries emphasized the need to address these gaps in developing investment cases for CRD at national level.

> "*You perform a study and show them the results. How many are affected? How many are disabled? How many lose their jobs? How many houses lose support? If you provide such work, you might be able to convince the (national treasury) officials*" *(KII-S5, Gezira State, Sudan MoH official).*

Lack of donor prioritisation decreases budgetary allocation

The health system financing arrangements in all the study countries were reported as being heavily dependent on external partners. CRD was perceived to be given low priority by donors because of their generally non-infectious nature and the perception that they are less fatal than infectious diseases such as malaria, HIV/AIDS and TB, and maternal and child health conditions. Even within the NCD departments in the study countries, efforts and resources are majorly directed towards cancer, diabetes, hypertension, and other cardiovascular diseases.

> *"We have some reasonable data on the burden of asthma, but asthma doesn't do one thing, doesn't kill people that much and so because it doesn't kill, it doesn't get the attention it should get… everybody gets worried about things that kill (TB, malaria, HIV/AIDS, diabetes, cancer) and not those that give you chronic illness" (Kenya- KII-KD)*

Current priorities

Positive steps in data collection and policy development were also described. In response to a global spotlight on NCDs and recent increased donor interest, the Kenyan and Tanzanian governments plan to close the evidence gap by capturing CRD data in routine health facility tools, and subsequent nationwide surveys like the Demographic and Health Surveys.

> *"We are refining the tools to better capture the NCDs and have specific NCD registers like in the general outpatient settings (KII, Kenya-MOH, national level)*

At the time of data collection, Kenya was in the process of ratifying the Protocol to eliminate illicit trade in tobacco products, and Uganda had banned tobacco smoking in public, tobacco advertising, promotion and sponsorship, and limited sales. This suggests a heightened prioritization of an important risk-factor of CRD on the policy agenda, with potential for downstream policy interventions.

For peer review only - http://bmjopen.bmj.com/site/about/guidelines.xhtml

Service level

Key service level barriers arising from policy failures included challenges in accessing CRD diagnostics and medicines, limited reporting, and low confidence and skill in diagnosis and management. There was a disconnect between the perceptions of policy stakeholders, which reflected what was available on paper, and the front-line health workers, who reported feeling ill-equipped to attend patients in practice.

Lack of diagnostic equipment

Participants universally agreed that the entry point to CRD diagnosis in their TB-endemic contexts required the exclusion of active TB disease in chronic cough. Diagnostic capacity was defined as a combination of sputum screening, with the availability of radiology, lung function testing, skilled staff and diagnostic and treatment algorithms for the common CRDs (asthma, post TB lung disease and COPD). While each study country had chest clinics at tertiary hospitals with some level of equipment and expertise, only TB screening had been systematically decentralised at lower levels.

Challenges were described in each aspect of diagnosis, with patients often paying out-of-pocket costs. All countries reported delays in sputum screening. In some instances, these delays extended to more than a week and were associated with additional costs to patients and subsequent loss to follow up.

Finally, the widespread lack of (or dysfunctional) x-ray equipment and lack of qualified personnel reported in lower-level public facilities in Kenya, Malawi, Uganda, and Tanzania, meant that patients had to seek chest x-rays (and CT scans) from private healthcare providers, whose costs were unaffordable to most. The lack of peak flow meters and spirometry machines meant that patients requiring lung function testing were referred to the national teaching and referral hospitals.

> *"Yeah, there was a time, we also [provided spirometry] but …our machine broke down … when we see they are needing that spirometry we send them to Kenyatta National Hospital (Healthcare Worker, Kenya).*

In Tanzania, participants noted that despite the recommendation to have peak flow meters at the primary healthcare level, none were available and not a single facility (including the referral hospital) offered spirometry. In Kenya, Malawi, Sudan, and Uganda, the use of spirometry was reported to be limited to tertiary hospitals with international research collaborations, where training and equipment are maintained through grants from external partners. Although some policy level stakeholders thought otherwise, health workers at the lower-level hospitals in all countries expressed dissatisfaction with the lack of peak flow meters and spirometers. In Malawi, there were conflicting reports on the distribution of spirometry services across facilities. Whilst policymakers claimed that all tertiary hospitals had spirometers, healthcare workers reported that spirometers were only available at one teaching hospital as part of a research project.

Lack of CRD training and guideline dissemination

According to majority of key informants in Kenya, Uganda, and Tanzania most healthcare workers had not received training on the latest diagnostic procedures for CRD, including the use of spirometry:

> *"Honestly, we have not received any training for CRD; we used to go for TB trainings only" (IDI, Healthcare worker, Tanzania)*

Malawian participants described a successful pilot decentralising CRD training, but this was yet to be scaled-up nationally. Participants in all contexts emphasized the need improve in-service training countrywide. In-service training opportunities were *ad hoc* and often *'erratic'* (Sudan); mostly supported by specific programmes, including research projects (e.g. Uganda), non-governmental organizations and pharmaceutical companies promoting specific products; and characterised by low coverage of necessary staff.

> *"…we started a program with some collaborative partners, … they bring health workers from some districts, and we come and do modules to understand for example what asthma is, how*

For peer review only - http://bmjopen.bmj.com/site/about/guidelines.xhtml

its diagnosed what is the best treatment, because in [medical] school frankly, people don't learn so much for the benefit of the wider community (MOH consultant physician Uganda)."

Most of the available training focused on TB. In Tanzania this was to the exclusion CRD. In Kenya, CRD were added on to TB training. Healthcare workers in Kenya reported difficulties in accessing professional training, within the devolved government setup. Lack of financial sponsorship from government, and a requirement that they resign if they applied for fulltime study imposed steep opportunity costs.

Standard processes of communicating guidelines from national policy level to service delivery levels were felt to be inadequate; for example, healthcare workers in Kenya described learning about asthma management guidelines incidentally, during internet use or informal discussions with colleagues. There was limited awareness of treatment guideline updates with many clinicians still prescribing available oral salbutamol instead of inhalers, despite lack of evidence for efficacy. Likewise, in Tanzania, although the CRD guidelines were available on the Ministry of Health website and copies were seen at the sites, participants seemed unaware of their content.

Limited availability of drugs and lack of confidence in management

Restrictive policy guidelines and user-fees imposed barriers to accessing corticosteroid inhalers. For example, beclomethasone is classified as a central facility level medicine in Malawi, that required international procurement protocols prone to delays of 4-7 months. In Tanzania only bronchodilators, injectable and oral steroids for acute asthma are allowed at lower levels of service provision. Similarly, in Uganda some medicines were restricted at the primary level. In contrast in Kenya bronchodilators and corticosteroids have been included in pharmacy order forms for primary health care facilities where they are provided free, implying shifts in practice to enhance access. In Sudan, asthma medications including inhalers and corticosteroids are supposed to be provided free of charge especially for patients in acute attacks. However, these are often not available in emergency rooms at both district and national levels, and when available, they are not always free of charge.

"Some of the services for asthma might be provided for free in the emergency room, but the majority of it is not provided for free although it should [be]" (KII-S4, Gezira State MoH official).

Additionally, respondents from all five countries reported frequent drug stockouts even of the few CRD treatments included on essential medicines lists. At the time of data collection, the Kenya Medical Supplies Authority had stopped supplies to counties with outstanding bills. At primary care level in Tanzania and Uganda drugs other than oral salbutamol were unavailable.

Frontline clinicians at primary and district level felt ill-equipped to meet the complexity of cases. Healthcare workers explained that they lacked confidence in managing chronic CRD, although acute asthma attacks would be treated (nebulisers, oral aminophylline and adrenalin injections were all mentioned).

"We end up using treatments we are not supposed to (for example oral salbutamol) because that is what is available (Uganda - Clinical officer HC III).

Key informants in Kenya and Uganda, mentioned measures to enhance treatment capacity through review of the curricula for in-service training of clinical and medical officers, and for Kenya, the development of 'module 13' for community health volunteers, on identifying and managing asthma.

"We are working closely with KMTC (Kenya Medical Training College) itself, the Kenya Clinical Officers Council and the Nursing Council of Kenya to give NCD's more prominence in the pre-service training (KII_MOH, Kenya)

Most healthcare workers, however referred chronic asthma and other undiagnosed CRD conditions to higher level facilities, but even at this level limited capacity for management was described. All five countries reported very few pulmonologists. Malawi for example, had only one specialist respiratory clinician, who often did not receive referrals because of low awareness among healthcare workers.

Poor reporting and reduced health care worker awareness

For peer review only - http://bmjopen.bmj.com/site/about/guidelines.xhtml

Low healthcare workers' diagnostic capacities implied that subsequent data inputs into the health information system may not provide reliable evidence of the CRD disease burden. Furthermore, the appropriate coding of diseases overall, is undermined by shortage of health information officers, and essential reporting tools.

> *"…then you find most of our medical personnel record "chronic cough" which is not a diagnosis … we are championing for this cadre of staff (health records information officers) because they are very critical otherwise, we would continue making wrong decisions based on poor data (KII-Non-State Actor, Kenya)*

This poor reporting was felt in turn to reduce awareness of CRD among health care workers of the need for clinical follow-up and of the overall scale of the problem.

DISCUSSION

Based on data from five Sub-Saharan African countries (Kenya, Malawi, Sudan, Tanzania, and Uganda) we identified how neglect of CRD at strategic and service levels form two intersecting vicious cycles as illustrated in Figure 1.

Figure 1

Whilst specific weaknesses varied by context, common cycles were discernible. At strategic policy level, low diagnostic capacity, weak recording and reporting systems limited the availability of reliable data on the burden of lung diseases, negatively affecting inclusion in policy, and in turn budgetary allocations for diagnostic equipment, training, and medicines. At the service level, lack of budgetary allocations for equipment and training of staff constrained diagnostic capacity, which, along with limited availability of appropriate medicines reduced service delivery quality and collection of appropriate data within healthcare facilities. Providers felt frustrated at their lack of capacity to diagnose and treat these conditions; they were aware that that their own skills to support patients with CRD over time were limited.

To strengthen health systems in these countries and others in similar contexts, it will be important to break these vicious cycles,[25] although the most strategic entry points may vary by country. For example, variations in the development of policy strategies specific to lung health suggest policy change is more important in some contexts with NCD policy and TB control policy both providing possible avenues of entry[26]. Improvements in diagnostic capacity, along with improving accuracy of reporting, may create impetus for policy commitments and corresponding budgetary allocations, required to improve availability of medicines. Concurrent improvements in systems 'software' such as communication, advocacy, and accountability are required to realise policy changes in practice[27, 28]. For example, strategic communication of local data and accountability for responsiveness to this is needed, not only on burden of disease and service use but also on stockouts, broken equipment, or vacant posts[29]. Ultimately a team approach (including managers, clinical providers, health information officers and community actors) will be needed to ensure a coherent approach and feed-back loops. The World Health Organization, and civil society groups, such as professional thoracic societies and researchers have an important role of developing a sustainable integrated response through their guidelines and practice in LMIC contexts. Effective communication of guidelines and protocols needs to be accompanied by operational plans to enact them. For example, it is now clear that treating asthma patients with short acting beta agonists alone is associated with risk (exacerbations and thus likely deaths) even in mild asthma. This has led to a revision of the current Global Initiative for Asthma (GINA) guidelines for asthma control advocate use of inhaled corticosteroid-formoterol for the management of mild asthma and yet many of the study countries restrict availability at the primary health care facilities most likely to see cases[30].

For peer review only - http://bmjopen.bmj.com/site/about/guidelines.xhtml

It is likely to be too simplistic to assume that changes at one point in the cycle will lead to the desired changes in a linear way throughout the cycle, since challenges in a complex system are likely to be multi-causal[31]. Evidence is only one, often small, element in policymaking[32]. In turn, written policy may not be translated into budgetary allocations or materialise as equipment and medicine availability due to intense budgetary competition; the role of donor priorities; 'leakage' of resources through the system; procurement and maintenance challenges, amongst others. Decentralisation offers opportunities for local (burden of disease) data and demands to influence budgetary allocations, and for enhancing primary healthcare services bringing CRD services closer to patients' residence. Decentralization may however also mean greater political struggles over priorities[33, 34] and increased fragmentation across the national health system. In many contexts, inadequate human resources and high staff attrition rates in rural areas provides a significant challenge to maintaining trained providers. Interventions in one part of the system may have unintended consequences in another[28]; where provider workloads are high, improved diagnostic capacity for certain diseases may deprioritise others, including by increasing service utilisation. Efforts will therefore be needed to track changes throughout the system, and beyond CRDs.

Finally, management of CRD, like other chronic conditions, requires continuity of care, effective linkage between health facilities and community systems, and patient empowerment[12, 35]. Significant communication is therefore required by providers, including community health workers, to increase awareness of CRD and support patients with transition to self-management of their conditions.

Health systems in LMICs are currently designed to respond to acute, mostly communicable, illnesses usually through disease-specific vertical programmes. Despite some adaptation to treat people living with HIV, these systems often respond poorly to chronic conditions such as CRD. Key features of care models for other non-communicable diseases which could be adapted are having ear marked funding for organized and equipped healthcare teams, sustained supply of medicines, continuity of care, and strong linkages between healthcare facilities and community health systems. Integration of policy and services has benefits of a more holistic approach in addressing multimorbidity and can for example reduce poor prescribing practice [36]. Rather than adding a new vertical programme, or a new set of diseases to existing programmes, we advocate for a system-wide approach to a range of chronic diseases.

Strengths and limitations

The main strengths and limitations both relate to the fact that the five studies were conducted independently, by different research teams with variations in the topic guides allowing for contextual insights. This allowed us to compare local strategies in dealing with CRD and share experiences and learn from them. The KII and IDI are appropriate in investigating and triangulating perspectives of study participants, enhancing rigour in the data collection. A larger number of district hospitals were sampled in Sudan and a focus on urban sites in Kenya, Malawi and Uganda with the inclusion of rural sites in Tanzania, and Sudan. While these independently conducted studies established similar findings in diagnosis and management of CRD, suggesting important systemic challenges across the SSA countries, the findings should nevertheless be interpreted with caution, as environmental exposures and service delivery contexts may differ with the distribution of healthcare facilities and professionals relatively higher in urban settings. We did not include CRD patients, community health workers and at-risk community members' perspectives in our study but focused on the overall, issues that affect the planning and care of the patients. Due to constraints in data collection, saturation was not achieved in the Ugandan site.

Recommendations and conclusions

The COVID-19 pandemic has brought into sharp focus the urgent need to reform and strengthen healthcare systems to effectively respond to people with chronic health conditions, including CRD, an important risk factor to COVID-19 severity. In the five SSA countries studied, interlinked gaps at the policy strategic level, and healthcare delivery levels, undermine appropriate provision of services for people with CRD. Lack of diagnostic capacity is a major link between the vicious cycles, influencing

For peer review only - http://bmjopen.bmj.com/site/about/guidelines.xhtml

both the ability to manage cases within services and lack of accurate data on CRD to inform policy responses and resource allocation. Improvements are required across all key elements of the service delivery systems, including pre- and in-service training for diagnosis and management, guideline dissemination, diagnostic equipment, recording and reporting. Additionally, there is urgent need to enhance reliable, affordable access to drugs, particularly the inhaled corticosteroid-formoterol inhaler in the early stages of asthma management in children, adolescents, and adults[37]. Enhancing collection of population-level data to ascertain the true burden of disease may be an important entry point to drive policy change ensuring that people living with CRD are not 'left behind' in the development of universal healthcare.

Figure 1 caption

Figure 1: A vicious cycle of neglect of CRD at the strategic policy level and service level

For peer review only

For peer review only - http://bmjopen.bmj.com/site/about/guidelines.xhtml

Author contributions

1. Stephen Mulupi- literature search, data collection, analysis, interpretation, writing
2. Irene Ayakaka: literature search, data collection & analysis, interpretation, writing
3. Nicole Kozak: literature search data collection & analysis, writing
4. Elizabeth Shayo: Data collection & analysis, writing
5. Elhafiz Abdalla: Data collection & analysis, writing
6. Rashid Osman: Data collection & analysis, writing
7. Stella Mpagama: Data collection & analysis, writing
8. Rachel Tolhurst: Study design, data analysis, interpretation, writing
9. Uzochukwu Egere: Study design, interpretation, writing
10. Martha Chinouya: Study design, interpretation, writing
11. Kingsley Chikaphupha: data analysis, interpretation, writing
12. Asma El Sony: Interpretation, writing
13. Hellen Meme: Interpretation, writing
14. Rose Oronje: Interpretation, writing
15. Nyanda Elias Ntinginya: Interpretation, writing
16. Angela Obasi: Study design, Interpretation, writing
17. Miriam Taegtmeyer: Study design, data analysis, interpretation, writing

Declaration of competing interests

None declared

Patient and Public involvement:

Patients or the public were NOT involved in the design, or conduct, or reporting, or dissemination plans of our research

Data availability Statement

No additional data available

For peer review only

For peer review only - http://bmjopen.bmj.com/site/about/guidelines.xhtml

References

1. World Health Organization. Non communicable diseases, Fact sheet. In: WHO, editor. 2017.
2. Soriano JB, Abajobir AA, Abate KH, Abera SF, Agrawal A, Ahmed MB, et al. Global, regional, and national deaths, prevalence, disability-adjusted life years, and years lived with disability for chronic obstructive pulmonary disease and asthma, 1990–2015: a systematic analysis for the Global Burden of Disease Study 2015. The Lancet Respiratory Medicine. 2017;5(9):691-706
3. Soriano JB, Kendrick PJ, Paulson KR, Gupta V, Abrams EM, Adedoyin RA, et al. Prevalence and attributable health burden of chronic respiratory diseases, 1990–2017: a systematic analysis for the Global Burden of Disease Study 2017. The Lancet Respiratory Medicine. 2020;8(6):585-96
4. Marais BJ, Loennroth K, Lawn SD, Migliori GB, Mwaba P, Glaziou P, et al. Tuberculosis comorbidity with communicable and non-communicable diseases: integrating health services and control efforts. The Lancet infectious diseases. 2013;13(5):436-48
5. Samb B, Desai N, Nishtar S, Mendis S, Bekedam H, Wright A, et al. Prevention and management of chronic disease: a litmus test for health-systems strengthening in low-income and middle-income countries. The Lancet. 2010;376(9754):1785-97
6. World Health Organization. Global Tuberculosis Report, 2019. Geneva: World Health Organization; 2019.
7. Byrne AL, Marais BJ, Mitnick CD, Lecca L, Marks GB. Tuberculosis and chronic respiratory disease: a systematic review. International Journal of Infectious Diseases. 2015;32:138-46
8. Amaral AF, Coton S, Kato B, Tan WC, Studnicka M, Janson C, et al. Tuberculosis associates with both airflow obstruction and low lung function: BOLD results. European Respiratory Journal. 2015;46(4):1104-12
9. Menezes AMB, Hallal PC, Perez-Padilla R, Jardim J, Muino A, Lopez M, et al. Tuberculosis and airflow obstruction: evidence from the PLATINO study in Latin America. European Respiratory Journal. 2007;30(6):1180-5
10. Manji M, Shayo G, Mamuya S, Mpembeni R, Jusabani A, Mugusi F. Lung functions among patients with pulmonary tuberculosis in Dar es Salaam – a cross-sectional study. BMC Pulmonary Medicine. 2016;16(1):58
11. Banda HT, Thomson R, Mortimer K, Bello GA, Mbera GB, Malmborg R, et al. Community prevalence of chronic respiratory symptoms in rural Malawi: Implications for policy. PloS one. 2017;12(12):e0188437
12. Bischoff A, Ekoe T, Perone N, Slama S, Loutan L. Chronic disease management in Sub-Saharan Africa: whose business is it? International Journal of Environmental Research and Public Health. 2009;6(8):2258-70
13. Siddharthan T, Grigsby M, Morgan B, Kalyesubula R, Wise RA, Kirenga B, et al. Prevalence of chronic respiratory disease in urban and rural Uganda. World Health Organization Bulletin of the World Health Organization. 2019;97(5):318-27
14. Meghji J, Nadeau G, Davis KJ, Wang D, Nyirenda MJ, Gordon SB, et al. Noncommunicable lung disease in sub-Saharan Africa. A community-based cross-sectional study of adults in urban Malawi. American journal of respiratory and critical care medicine. 2016;194(1):67-76
15. van Gemert F, van der Molen T, Jones R, Chavannes N. The impact of asthma and COPD in sub-Saharan Africa. Primary Care Respiratory Journal. 2011;20(3):240
16. Arokiasamy P, Kowal P, Capistrant BD, Gildner TE, Thiele E, Biritwum RB, et al. Chronic noncommunicable diseases in 6 low-and middle-income countries: findings from wave 1 of the World Health Organization's study on global Ageing and adult health (SAGE). American journal of epidemiology. 2017;185(6):414-28
17. Kibirige D, Sanya RE, Nantanda R, Worodria W, Kirenga B. Availability and affordability of medicines and diagnostic tests recommended for management of asthma and chronic obstructive pulmonary disease in sub-Saharan Africa: a systematic review. Allergy, Asthma & Clinical Immunology. 2019;15(1):14

For peer review only - http://bmjopen.bmj.com/site/about/guidelines.xhtml

18. Kibirige D, Kampiire L, Atuhe D, Mwebaze R, Katagira W, Muttamba W, et al. Access to affordable medicines and diagnostic tests for asthma and COPD in sub Saharan Africa: the Ugandan perspective. BMC pulmonary medicine. 2017;17(1):179
19. Crotty M. Introduction: The research process. The foundations of social research: Meaning and perspective in the research process. 1998:1-17
20. Scotland J. Exploring the philosophical underpinnings of research: Relating ontology and epistemology to the methodology and methods of the scientific, interpretive, and critical research paradigms. English language teaching. 2012;5(9):9-16
21. World Health Organization. EVERYBODY'S BUSINESS. STRENGTHENING HEALTH SYSTEMS TO IMPROVE HEALTH OUTCOMES: WHO'S FRAMEWORK FOR ACTION. 2007
22. Ritchie J, Spencer L, Bryman A, Burgess RG. Analysing qualitative data. 1994.
23. Government of the United Republic of Tanzania.

Strategic and Action Plan for the Prevention And Control of Non Communicable Diseases in Tanzania 2016 – 2020. Ministry of Health, Community Development, Gender, Elderly and Children

2016.04-03-2020
24. Ministry of Health. National Strategic Plan for Tuberculosis, Leprosy and Lung Health 2019-2023. National Tuberculosis, Leprosy and Lung Disease Program; 2019.
25. English M, Irimu G, Agweyu A, Gathara D, Oliwa J, Ayieko P, et al. Building learning health systems to accelerate research and improve outcomes of clinical care in low-and middle-income countries. PLoS medicine. 2016;13(4):e1001991
26. Juma PA, Mohamed SF, Mwagomba BLM, Ndinda C, Mapa-Tassou C, Oluwasanu M, et al. Non-communicable disease prevention policy process in five African countries authors. BMC public health. 2018;18(1):961
27. Gilson L. Trust and the development of health care as a social institution. Social science & medicine. 2003;56(7):1453-68
28. De Savigny D, Adam T. Systems thinking for health systems strengthening: World Health Organization; 2009.
29. Sacks E, Morrow M, Story WT, Shelley KD, Shanklin D, Rahimtoola M, et al. Beyond the building blocks: integrating community roles into health systems frameworks to achieve health for all. BMJ global health. 2019;3(Suppl 3):e001384
30. Global Initiative For Asthma. Pocket guide for asthma management and prevention (For adults and children older than 5 years). In: GINA, editor. Fontana, USA 2020.
31. Pourbohloul B, Kieny M-P. Complex systems analysis: towards holistic approaches to health systems planning and policy. SciELO Public Health; 2011. p. 242-.
32. Maher A, Sridhar D. Political priority in the global fight against non–communicable diseases. Journal of global health. 2012;2(2)
33. McCollum R, Limato R, Otiso L, Theobald S, Taegtmeyer M. Health system governance following devolution: comparing experiences of decentralisation in Kenya and Indonesia. BMJ global health. 2018;3(5):e000939
34. Barasa EW, Cleary S, English M, Molyneux S. The influence of power and actor relations on priority setting and resource allocation practices at the hospital level in Kenya: a case study. BMC health services research. 2016;16(1):536
35. Rabkin M, El-Sadr WM. Why reinvent the wheel? Leveraging the lessons of HIV scale-up to confront non-communicable diseases. Global public health. 2011;6(3):247-56
36. Epping-Jordan J, Pruitt S, Bengoa R, Wagner EH. Improving the quality of health care for chronic conditions. BMJ Quality & Safety. 2004;13(4):299-305
37. Reddel HK, Bacharier LB, Bateman ED, Brightling CE, Brusselle GG, Buhl R, et al. Global Initiative for Asthma (GINA) Strategy 2021–Executive summary and rationale for key changes. The Journal of Allergy and Clinical Immunology: In Practice. 2021

For peer review only - http://bmjopen.bmj.com/site/about/guidelines.xhtml

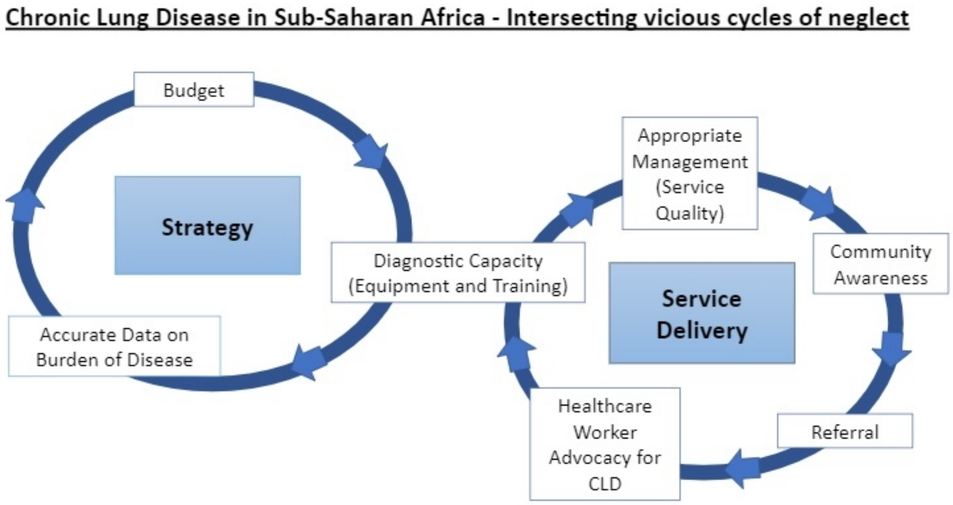

200x103mm (96 x 96 DPI)

Appendix 1: Key informant interview guide for national-level Stakeholders-Kenya

1. Please describe to me your role in this organization. *Probe[How long have you been in this role? Which areas do you work within Kenya? Do you have other work outside the country?]*

2. Overall, how long have you been involved in NCD-related activities? Probe (what exactly drew you to NCD work?)

NCD management is gaining attention across the world. In which ways are you involved in making policies for NCD control in Kenya? *(Probe: is there a specific policy that you are particularly involved in? which one? How are you involved? Policy content, research, advocacy, financing, service provision)*

3.

4. Is chronic respiratory disease one of the NCDs you work with? (If yes – what do you mean by chronic respiratory disease? Please describe the work you do in relation to this? Whose responsibility is CRD in Kenya?

5. What do you think of the health policy on CRD in Kenya? (*Probe:* Is it appropriate? How well is it implemented? What are the key challenges in its implementation? What do you think could be done differently to make implementation better?).

6.

7. Please tell me who your partners are within Kenya. (Explain 'partners' to mean any organizations that you collaborate in any way on CRD work. Probe: national government, county governments, international organizations, CBO, politicians, media, any others?)

8. *If national/county governments are not mentioned in 4- what is your relationship with national/county government? How accessible are you to government decision makers? Are there counties that you partner with? Do you have representation in any of the government committees?*

9. How do you relate with the partners you have mentioned? *(Probe for joint policy development activities, representation in technical committees locally and internationally, advocacy, research, financing, political support).*

10. Among these partners, who do you think is the most powerful in influencing NCD policy development in Kenya? *(Probe: Why do you say so? Is there a partner that you feel should be included in NCD management efforts in Kenya? Why?)*

For peer review only

11. What has worked well in terms of patient care pathways? Why do you say so?

12. What has not worked well so far in terms of patient care pathways? *(Probe: Why do you say so? In your opinion, what could be done differently?)*

13. Over the next 5 years, what are your priorities in NCD control? What about for chronic respiratory disease? What opportunities do you see?

Thank the respondent and end the interview.

For peer review only

Appendix 2: Key informant interviews for policy-makers in national and county governments

**Institution/ Department**	
**Duration**	

1. Please describe to me your role in this department Probe[How long have you been in this role?]
2. Overall, how long have you been involved in NCD-related activities? *(Probe: what exactly drew you to NCD work?)*
3. NCD management is gaining attention across the world. In which ways are you involved in making policies for NCD control in Kenya? (Probe: is there a specific policy that you are particularly involved in? which one? How are you involved?)
4. Is chronic respiratory disease one of the NCDs you work with? (probes - If yes – what do you mean by chronic respiratory disease? Can you describe the work you do in relation to this? If no – can you share more about why not?)
5. Whose responsibility is CRD in Kenya?
6. Please tell me who your key partners are within Kenya. (Explain 'partners' to mean any organizations that you collaborate in any way on CRD work. Probe: national government, county governments, international organizations, CBO, politicians, media, any others?)
7. How do you relate with the partners you have mentioned? (Probe for joint policy development activities, representation in technical committees locally and internationally, advocacy, research, financing, political support).
8. Among these partners, who do you think is most influential CRD policy development in Kenya? Why do you say so? Is there a partner that you feel should be included in CRD management efforts in Kenya? Why?
9. (As appropriate) What is your relationship with national/county government in CRD management? How do you ensure that policy guidelines are adhered to? Do you offer any trainings?

For peer review only

10. What has worked well in terms of patient care pathways? Why do you say so?

11. What has not worked well in terms of patient care pathways so far? Why do you say so?

10. Over the next 5 years, what are your priorities in NCD control? What about for chronic respiratory disease? What opportunities do you see?

Thank the respondent and end the interview.

For peer review only

For peer review only

APPENDIX 2_KEY INFORMANT INTERVIEWS WITH STAKEHOLDERS_SUDAN AND TANZANIA

IMPALA Phase1 Topic Guide Two_V2.0_23July2018

Interview Topic Guide for Health Workers (including patient vignettes)

Interview ID NO: _______________ Facilitator Initials: ____________ Note-taker Initials: _________

Type of Participant _________________ Audio file ID __________

Country/Health facility: ______________________ Date of Interview ____________________

Where do you work? ______________________________

What is your job title? ____

How long have you worked as (job title)? ___________

Current Disease Priorities within the Health Facility

Can you tell me more about the services that you provide at this health facility?

What are the main priority diseases and illnesses for this facility?

Why are these the main priorities?

Can you tell me about the context of diseases such as Asthma, COPD and TB at this facility?

Readiness for integration of CLD services

Can you tell me more about the services you provide for patients with TB and other lung diseases at this facility?

- Prompt specifically for: Asthma, COPD, occupational lung disease
- How does this link to the community health system? e.g. community health workers, informal health providers etc.
- Please tell me more about how CLDs are diagnosed?
- Please tell me more about how CLDs are managed?

How ready do you feel your health facility is to be able to properly manage and integrate CLDs into routine service/programme delivery?

- ***Case Detection:***
    i. What equipment do you feel is needed to diagnose and manage patients with CLDs?
    ii. What are the challenges with ensuring this equipment if available, functional and used within your facility? What helps?

For peer review only

iii. Do you have a standard case definition for any chronic lung diseases (asthma, COPD etc) to guide you in case detection? (if yes, get some details and request a copy).

iv. Based on your experience in this facility can you comment on how long it takes to detect CLD? (probe for early/late detection, severity of cases when received)

- ***Standardised treatment and effective drug supply, including medical products and technology:***

i. Are there algorithms for staff in your facility to follow to diagnose and manage patients with CLDs? If yes, who was involved with developing these? Who showed you how to use them? What do you think about these algorithms?

ii. Are drugs for managing CLDs included in essential drug lists for your facility? Why? Why not? How available are these medicines? How often do you receive medicines? How much do they cost?

- ***Service delivery***

i. Can you tell me more about the services currently available for patients with TB and other chronic lung diseases (e.g. asthma and COPD) at your facility?

ii. How do you think the quality of these services could be improved?

iii. Based on your experiences, what do you think effective CLD care looks like?

iv. What do you think are the main needs for patients with CLDs?

v. How prepared is your facility to meet these needs? What changes need to be made to meet these needs?

b. How do you think services for CLD could be integrated with other services? (e.g. TB/NCDs etc).

- ***Health workforce***

i. Who do you think should be involved in the management of CLD within your facility? What should their roles and responsibilities be?

ii. Are there adequate numbers of staff to manage CLDs in your facility? Why? Why not?

iii. How are staff currently trained to manage CLDs? (pre-service training, in-service training?) How does this vary depending on job role? How could this be improved?

iv. What supervision structures are in place for health staff involved in the management of CLD within this facility? What works well about supervision? What could be improved?

v. What is needed to ensure staff are ready to diagnose and manage patients with CLDs?

- ***Reporting, recording of CLD cases and analysing CLD data for action***

i. What data is currently captured in relation to CLD in existing health information management systems?

ii. Where is CLD data reported? And how often? (probe for the flow of information)

For peer review only

iii. What analysis is done regarding CLDs? How is this information used?

What are the main barriers to diagnosing and managing patients with CLDs?

- How can these barriers be overcome?

What are the things that help with diagnosis and management currently?

What would make it easier to diagnose and manage patients with CLDs?

What do you think would be the main benefits of integrating the management of CLDs within existing health programmes?

- Which programmes or services would allow for integration?

What do you think would be the main challenges of integrating the management of CLDs within existing health programmes?

- How can these potential threats be avoided?

For peer review only

The following type of patient vignettes will also be used with health workers to assess their current skills for CLD management. It will be made clear to the participants that this is not a test and the results of the study will not be shared with supervisors/colleagues etc.

EXERCISES – Module 9

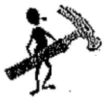

Revise what you have learnt and practise filling in the Asthma Treatment Card and Quarterly Reports.

The following patients have arrived with respiratory symptoms and are suspected cases of asthma. Answer the questions. If it is a case of persistent asthma, record the information on the Asthma Treatment Card.

Patient 1
Man, 60 years old. Retired from work in textile industry.
Clinical signs:
- episodes of coughing with breathlessness and sputum expectoration for the last 3 weeks
- has increased purulence of sputum for the last 5 days
- traces of blood in expectoration once or twice
- temperature has been 38 degrees for the last 3 days

History:
- episodes of coughing in the morning for the last 5 years
- breathlessness after effort for the last year
- temperature has been normal, but 5 kg weight loss over the last year
- no history of allergic disease or tuberculosis
- no known co-morbidity
- Smoking: ex-smoker (smoked 30 cig/day over period of more than 20 years; stopped smoking 3 years ago)

Clinical examination:
- Normal apart from some ronchi and wheezing.

1. What is or what are your suspected diagnoses?…………………………………
2. What do you decide to do? What do you prescribe?
……………………………………………………………………………………………
……………………………………………………………………………………………
……………………………………………………………………………………………

After 8 days, patient improves: coughing, expectoration and breathlessness decrease. Weight is stable. Bacteriological examinations are negative, in particular AFB smear.
Predicted PEF: 536 l/min
PEF measurements are as follows:
PEF before bronchodilator = 280 l/min
PEF after bronchodilator = 290 l/min
However, as the breathlessness has not completely disappeared, the doctor prescribes a short course of prednisone, with salbutamol as needed. After this treatment, the patient comes back and the PEF is measured:
PEF after bronchodilator after 8 days of prednisone = 300 l/min

3. What is the patient's PEF variability?
……………………………………………………………………………………………
……………………………………………………………………………………………
4. What is the most probable diagnosis?
……………………………………………………………………………………………
5. What is the severity of the disease?
……………………………………………………………………………………………
……………………………………………………………………………………………

If your diagnosis is asthma, complete an Asthma Treatment Card.

6. Teach/Check patient inhaler technique

7. Teach/Check patient ability to self-control attacks
8. How would you manage the patient if he had an acute asthma attack?
9. Please describe how you would respond if the patient told you he was no longer able to carry out his work, due to his breathlessness and he was feeling quite low as a result
    a. What else might you want to consider when managing this patient given his recent low mood?
    b. Are there any other service sectors that you might want to link this patient with?

For peer review only

Appendix 3_a: Key informant interview guide for Stakeholders- Uganda

1. Please describe your current role in this organization. Probe [How long have you been in this role? What other roles /positions related to non-communicable diseases do you hold?]

2. Overall, how long have you been involved in NCD-related activities? Probe [how did you come to be involved in NCD work?)

3. NCDs are recognised for being the biggest cause of disease worldwide and are steadily gaining attention in LMICs where they cause avertable premature deaths, what policies are in place for their management in Uganda? [what is the extent of attention received for NCDs in Uganda?]

4. What role do you play in making policies for NCD management in Uganda? [How involved are you in the policy making process? What specific policy stage where you most involved; content, research, advocacy, financing, service provision?]

5. In what ways do you think the policy for NCD management in Uganda is lacking? In which ways is it strong? [what changes would you make to the policy if you had to?]

6. How would you define Chronic lung diseases? In your opinion how big of a problem are CLDs in Uganda?

7. What specific policies are in place for CLD management in Uganda? [Apart from TB policy that is explicit and managed by the TB control program? What guides CLD management at health facilities in Uganda?]

8. Who are some of your partners within Uganda. ['Partners' here refers to any institutions, persons, organizations that you collaborate in any way on CLD work. Probe: national government partners, international organizations and donors, NGOs (international and local), community based organisations, politicians, media, any others? What roles do they mainly play?]

9. How do you relate with the partners you have mentioned? [Probe for joint policy development activities, representation in technical committees locally and internationally, advocacy, research, financing, political support].

10. Among these partners, who do you think are the most powerful in influencing NCD/CLD policy development in Uganda? Who of the partners have a high interest in CLD policy? [Probe: Why do you say so? Who are other partners that you feel should be included in NCD/CLD management efforts in Uganda? Why?]

11. What are the main sources of funding for NCD/CLD management in Uganda?

12. What in your opinion are barriers to implementation of CLD policy at the frontline/health facility level? [what could enhance the implementation of policies at the health facility level?]

13. Who is responsible for ensuring policies are adhered to? [Probes; What role do you have in ensuring that policy guidelines are adhered to? Do you offer any policy related training to frontline workers and implementers of the policies?]

14. What role can patients and their families play in the policy development process for NCD/CLD management in Uganda?

15. Over the next 5 years, what are your priorities in NCD/CLD management in Uganda?

For peer review only

Thank the respondent and end the interview.

Appendix 3_b: Key informant interviews for clinical services directors/facility in-charges at KCCA health facilities- Uganda

1. Please describe your role (s) in this health facility? [Probes: How long have you held this role?]

2. How would you define chronic lung diseases?

3. I am interested in what policies are in place for the management of CLD in this facility and how management of CLDs happens in this facility. Do you have guidelines specific to CLD management? What are your experiences in implementing those guidelines? [Probes; what services for the diagnosis and management of CLD are available? Screening, diagnostics, treatment, follow up.]

4. What was the role your role in development of CLD management policy? What was your role in designing the implementation of the CLD policy? [Probes; if not involved who was? How does the implementation work?]

5. What has worked well so far in treating CLD patients in this facility? [Explain each of the things that have worked well, what has not worked well? Further probes; drugs and medications, patient literacy, patient education and family support?]

6. How can CLD services be improved in this facility?

7. What in your opinion can be done to improve the policies and their implementation? [Do you think you should be involved as a frontline manager in the development of CLD policy? Why? Who else should be involved?]

8. What role can patients play in the development of CLD policy and in the implementation of policy?

9. What in your opinion is the role of involving frontline health workers in developing policies?

10. What is the place of training on a policy at implementation level? [Probes; Were health workers here trained in CLD management? How often does such training occur and who organises the trainings?]

(Respondent may ask questions)

For peer review only

Appendix 4_ In-depth interview for healthcare workers: clinical staff e.g. nurse, facility in-charge, CRD specialist

1. Please describe to me your roles in this department (probe: how long have you been in this role? How big is your team? Please describe their roles?)

2. Do you see patients with chronic respiratory diseases? What conditions do you mostly see? How would you know if someone has CRD?. What challenges do you face in diagnosing CRD?

3. What makes people with chronic respiratory symptoms come here? (probe- are they referred here? From where? Who refers them?) Please describe what happens to these patients from the time they come in. (Probe: Screening and diagnostics, treatment, follow up, health education).

4. How does referral work here? Where are patients referred from? Where to? How frequently do you refer patients? After referral, do you maintain contact with the patient? How? Do you get feedback? What happens after discharge?

5. Please tell me what has worked well so far in: diagnosis of g CRD patients

6. Please tell me what has **not** worked well in diagnosis of CRD patients. (For each of the observations, why do you say so?)

7. Please tell me what has worked well so far in: treatment of CRD patients.

8. Please tell me what has **not** worked well in treatment of CRD patients. (For each of the observations, why do you say so?) Probe availability of drugs, timeliness of county governments, affordability of drugs, follow ups).

9. Did you receive any training specific to CRD patients? When was that? Do you have guidelines specific to CRD management? What are your experiences in implementing those guidelines?

10. What are your views of how services for CRD can be improved?

For peer review only

For peer review only

Appendix 4: In-depth interview guide for healthcare workers: Pharmacist

1. Please describe to me your roles in this department (how long have you been in this role? How big is your team? Please describe their roles?)

2. I am interested in understanding the process of treatment for patients who come with chronic respiratory problems. Please describe how these patients get services from the pharmacy. (Probe: prescriptions, drugs access).

3. Please tell me about the preferred drugs for CRD. Which of these do you stock? How do you procure these drugs? What are your experiences of availability of these drugs? Are there any that are covered for by NHIF?

4. Apart from drugs, do you procure any equipment used in the diagnosis of CRD e.g. peak flow meters?

5. Please tell me what has worked well so far in treating CRD patients.

6. Please tell me what has not worked well in treating CRD patients. *(Probe For each of the observations, why do you say so? availability of drugs, timeliness of county governments, affordability of drugs, follow ups).*

7. Did you receive any training on CRD management? (If yes: when?)

8. Do you have guidelines specific to CRD management? What are your experiences in implementing those guidelines?

9. What are your views of how services for CRD can be improved in this county?

For peer review only

Appendix 5: Interview guide for Community Health Extension Officer (CHEW)

1. Please tell me about your role as a CHEW.
2. Is chronic respiratory disease an issue in this CHU?
3. What is your involvement in CRD in your CHU?
4. Please describe how referrals for people with CRD to this facility works (probe: how patients are referred, any documentation from the CHV, records kept by CHEW).
5. How frequently are CRD patients referred? How does it work? What is your involvement?
6. After initial treatment, how do you follow up with patients' treatment? *(Probe: how do you get feedback about patients' condition, supporting follow up)*
7. Did you receive any specific training on CRD management? (If yes, when? Do you have any guidelines for supporting your community work? Have the CHV been trained in CRD case detection?)
8. Do you conduct any outreach services on CRD? How frequently do you do this? What is involved in the outreach?
9. Apart from county government, who are the other partners that you work with? (Probe: international organizations, local organizations)
10. What has worked well so far? Why do you say so?
11. What has not worked well so far? Why do you say so? What could be done differently to improve partnership between the health facility and community?

For peer review only

Appendix 6: In-depth Interview guide for Laboratory staff:

1. Please describe your roles in this department (how long have you been in this role? How big is your team? What does your day to day job involve?).

2. Please describe how patients with cough and/or breathlessness are referred to the lab? Please describe how sputum tests are done here? How frequently do you conduct these tests? Please describe the procedures involved in tests for people with cough and/or breathlessness?

3. In your experience, what are the major outcomes of the tests? How long does it take to get results? How are these results communicated to the patient? After lab, where does the patient go to?

4. Probe: what happens to patients with smear negative results for TB? Are other tests done? Are there patients who keep coming back to this facility? What do you do for them?

5. Please tell me what has worked well so far in testing people with chronic respiratory symptoms.

6. Please tell me what has not worked well in testing CRD patients. (Probe for each of the observations, why do you say so? availability of equipment, reagents).

7. Did you receive any training on CRD management? (If yes: when?)

8. Do you have guidelines specific to CRD management? What are your experiences in implementing those guidelines?

9. What are your views of how services for CRD can be improved in this county?

For peer review only

For peer review only

Appendix_5_Interview_guide healthcare workers_Sudan and Tanzania

IMPALA Phase1 Topic Guide Two_V2.0_23July2018
Interview Topic Guide for Health Workers (including patient vignettes)

Interview ID NO: _______________ Facilitator Initials: ____________ Note-taker Initials: _________	
Type of Participant _________________ Audio file ID __________	
Country/Health facility: _______________________	Date of Interview ____________________
Where do you work? ______________________________	
What is your job title? ____	
How long have you worked as (job title)? ___________	

Current Disease Priorities within the Health Facility

Can you tell me more about the services that you provide at this health facility?

What are the main priority diseases and illnesses for this facility?

Why are these the main priorities?

Can you tell me about the context of diseases such as Asthma, COPD and TB at this facility?

Readiness for integration of CLD services

Can you tell me more about the services you provide for patients with TB and other lung diseases at this facility?

- Prompt specifically for: Asthma, COPD, occupational lung disease
- How does this link to the community health system? e.g. community health workers, informal health providers etc.
- Please tell me more about how CLDs are diagnosed?
- Please tell me more about how CLDs are managed?

How ready do you feel your health facility is to be able to properly manage and integrate CLDs into routine service/programme delivery?

- ***Case Detection:***
    i. What equipment do you feel is needed to diagnose and manage patients with CLDs?
    ii. What are the challenges with ensuring this equipment if available, functional and used within your facility? What helps?

For peer review only

iii. Do you have a standard case definition for any chronic lung diseases (asthma, COPD etc) to guide you in case detection? (if yes, get some details and request a copy).

iv. Based on your experience in this facility can you comment on how long it takes to detect CLD? (probe for early/late detection, severity of cases when received)

- ***Standardised treatment and effective drug supply, including medical products and technology:***

i. Are there algorithms for staff in your facility to follow to diagnose and manage patients with CLDs? If yes, who was involved with developing these? Who showed you how to use them? What do you think about these algorithms?

ii. Are drugs for managing CLDs included in essential drug lists for your facility? Why? Why not? How available are these medicines? How often do you receive medicines? How much do they cost?

- ***Service delivery***

i. Can you tell me more about the services currently available for patients with TB and other chronic lung diseases (e.g. asthma and COPD) at your facility?

ii. How do you think the quality of these services could be improved?

iii. Based on your experiences, what do you think effective CLD care looks like?

iv. What do you think are the main needs for patients with CLDs?

v. How prepared is your facility to meet these needs? What changes need to be made to meet these needs?

b. How do you think services for CLD could be integrated with other services? (e.g. TB/NCDs etc).

- ***Health workforce***

i. Who do you think should be involved in the management of CLD within your facility? What should their roles and responsibilities be?

ii. Are there adequate numbers of staff to manage CLDs in your facility? Why? Why not?

iii. How are staff currently trained to manage CLDs? (pre-service training, in-service training?) How does this vary depending on job role? How could this be improved?

iv. What supervision structures are in place for health staff involved in the management of CLD within this facility? What works well about supervision? What could be improved?

v. What is needed to ensure staff are ready to diagnose and manage patients with CLDs?

- ***Reporting, recording of CLD cases and analysing CLD data for action***

i. What data is currently captured in relation to CLD in existing health information management systems?

ii. Where is CLD data reported? And how often? (probe for the flow of information)

For peer review only

iii. What analysis is done regarding CLDs? How is this information used?

What are the main barriers to diagnosing and managing patients with CLDs?

- How can these barriers be overcome?

What are the things that help with diagnosis and management currently?

What would make it easier to diagnose and manage patients with CLDs?

What do you think would be the main benefits of integrating the management of CLDs within existing health programmes?

- Which programmes or services would allow for integration?

What do you think would be the main challenges of integrating the management of CLDs within existing health programmes?

- How can these potential threats be avoided?

For peer review only

The following type of patient vignettes will also be used with health workers to assess their current skills for CLD management. It will be made clear to the participants that this is not a test and the results of the study will not be shared with supervisors/colleagues etc.

EXERCISES – Module 9

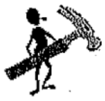

Revise what you have learnt and practise filling in the Asthma Treatment Card and Quarterly Reports.

The following patients have arrived with respiratory symptoms and are suspected cases of asthma. Answer the questions. If it is a case of persistent asthma, record the information on the Asthma Treatment Card.

Patient 1
Man, 60 years old. Retired from work in textile industry.
Clinical signs:
- episodes of coughing with breathlessness and sputum expectoration for the last 3 weeks
- has increased purulence of sputum for the last 5 days
- traces of blood in expectoration once or twice
- temperature has been 38 degrees for the last 3 days

History:
- episodes of coughing in the morning for the last 5 years
- breathlessness after effort for the last year
- temperature has been normal, but 5 kg weight loss over the last year
- no history of allergic disease or tuberculosis
- no known co-morbidity
- Smoking: ex-smoker (smoked 30 cig/day over period of more than 20 years; stopped smoking 3 years ago)

Clinical examination:
- Normal apart from some ronchi and wheezing.

1. What is or what are your suspected diagnoses?…………………………………
2. What do you decide to do? What do you prescribe?
……………………………………………………………………………………………
……………………………………………………………………………………………
……………………………………………………………………………………………

After 8 days, patient improves: coughing, expectoration and breathlessness decrease. Weight is stable. Bacteriological examinations are negative, in particular AFB smear.
Predicted PEF: 536 l/min
PEF measurements are as follows:
PEF before bronchodilator = 280 l/min
PEF after bronchodilator = 290 l/min
However, as the breathlessness has not completely disappeared, the doctor prescribes a short course of prednisone, with salbutamol as needed. After this treatment, the patient comes back and the PEF is measured:
PEF after bronchodilator after 8 days of prednisone = 300 l/min

3. What is the patient's PEF variability?
……………………………………………………………………………………………
……………………………………………………………………………………………
4. What is the most probable diagnosis?
……………………………………………………………………………………………
5. What is the severity of the disease?
……………………………………………………………………………………………
……………………………………………………………………………………………

If your diagnosis is asthma, complete an Asthma Treatment Card.

6. Teach/Check patient inhaler technique

7. Teach/Check patient ability to self-control attacks
8. How would you manage the patient if he had an acute asthma attack?
9. Please describe how you would respond if the patient told you he was no longer able to carry out his work, due to his breathlessness and he was feeling quite low as a result
    a. What else might you want to consider when managing this patient given his recent low mood?
    b. Are there any other service sectors that you might want to link this patient with?

For peer review only

Appendix 6_Topic Guide- Malawi

I really want to begin to understand diagnosis and treatment of chronic respiratory disease in the Malawi health system, and understand factors that have advanced and interfered with diagnosis and treatment

1. Tell me how your **job relates** to chronic respiratory disease (excluding TB). Probe: what is your experience with CRD

2. Tell me about medication supply and availability.
   Probe: what is your perspective on regulation, what is your experience and understanding of common obstacles that occur.
   Access to medication, constraints of:
   Market forces/transparency/donor agenda and funding

3. How do you see Malawian health system changing in recent years addressing chronic health conditions?
   Probe. Specifically CRD, what barriers seen in other countries experiencing.
   - Chronic Care clinic piloted in 2014 in area 25,
   - Package of essential NCD diagnosis and treatment in primary care was trailed in Kasungu District 2012, scale up 2015

4. How do you see clinicians using new Malawi **treatment guidelines** for Chronic respiratory disease diagnosis, and treatment? Spirometry/ Peak flow meter

5. What are some **problem-solving techniques** you use or see clinicians using to provide care to patients seeking medical care who do not have TB?

6. Can you tell me **influencers on actors and action** of NCD and CRD policy? Expand on system thinking in health decision making and policy design?
   Actors/context/process
   Policy communities/networks/venue
   Actors /institution that make decision

7. What do you consider to be the key issues that you see in Malawian health system that create barriers to better treatment of chronic respiratory disease?

   Tell me, have you heard about the Triage Project here in Malawi
   Probe: when did you become involved, what were some key events you saw happen, have you seen any predicted or unpredicted changes or challenges
   Anything else you would like to add?

For peer review only - http://bmjopen.bmj.com/site/about/guidelines.xhtml

For peer review only

For peer review only - http://bmjopen.bmj.com/site/about/guidelines.xhtml

Standards for Reporting Qualitative Research (SRQR)

O'Brien B.C., Harris, I.B., Beckman, T.J., Reed, D.A., & Cook, D.A. (2014). Standards for reporting qualitative research: a synthesis of recommendations. *Academic Medicine, 89(9)*, 1245-1251.

No. Topic	Item	Page
**Title and abstract**		
S1 Title	Concise description of the nature and topic of the study identifying the study as qualitative or indicating the approach (e.g., ethnography, grounded theory) or data collection methods (e.g., interview, focus group) is recommended	Title page
S2 Abstract	Summary of key elements of the study using the abstract format of the intended publication; typically includes objective, methods, results, and conclusions	Please refer to abstract page-2
**Introduction**		
S3 Problem formulation	Description and significance of the problem/phenomenon studied; review of relevant theory and empirical work; problem statement	Please refer to Page 3
S4 Purpose or research question	Purpose of the study and specific objectives or questions	Page 3
**Methods**		
S5 Qualitative approach and research paradigm	Qualitative approach (e.g., ethnography, grounded theory, case study, phenomenology, narrative research) and guiding theory if appropriate; identifying the research paradigm (e.g., positivist, constructivist/interpretivist) is also recommended	
S6 Researcher characteristics and reflexivity	Researchers' characteristics that may influence the research, including personal attributes, qualifications/experience, relationship with participants, assumptions, or presuppositions; potential or actual interaction between researchers' characteristics and the research questions, approach, methods, results, or transferability	Please refer to last sentence in the data collection section:<br><br>"Interviews were conducted by experienced qualitative researchers" pp4
S7 Context	Setting/site and salient contextual factors; rationale[a]	Context is described in 2nd paragraph of the

For peer review only

		introduction- pp3
S8 Sampling strategy	How and why research participants, documents, or events were selected; criteria for deciding when no further sampling was necessary (e.g., sampling saturation); rationale[a]	Methods section pp3. Healthcare workers and policy makers sampled for their roles in management of chronic lung diseases and policy making, respectively.
S9 Ethical issues pertaining to human subjects	Documentation of approval by an appropriate ethics review board and participant consent, or explanation for lack thereof; other confidentiality and data security issues	"Ethics" section, pp4.
S10 Data collection methods	Types of data collected; details of data collection procedures including (as appropriate) start and stop dates of data collection and analysis, iterative process, triangulation of sources/methods, and modification of procedures in response to evolving study findings; rationale[a]	Page 4, data collection section
S11 Data collection instruments and technologies	Description of instruments (e.g., interview guides, questionnaires) and devices (e.g., audio recorders) used for data collection; if/how the instrument(s) changed over the course of the study	Data collection, page 4
S12 Units of study	Number and relevant characteristics of participants, documents, or events included in the study; level of participation (could be reported in results)	Table 1 and 2
S13 Data processing	Methods for processing data prior to and during analysis, including transcription, data entry, data management and security, verification of data integrity, data coding, and anonymization/deidentification of excerpts	Intercountry analysis, page 4
S14 Data analysis	Process by which inferences, themes, etc., were identified and developed, including researchers involved in data analysis; usually references a specific paradigm or approach; rationale[a]	Please refer to the Intercountry analysis pp4, researcher roles listed under 'Author contributions' page 10
S15 Techniques to enhance trustworthiness	Techniques to enhance trustworthiness and credibility of data analysis (e.g., member checking, audit trail, triangulation); rationale[a]	Please refer to the Data collection section- verbatim transcripts (page 4); excerpts from transcripts used to illustrate specific findings (page 5)
**Results/Findings**		
S16 Synthesis and interpretation	Main findings (e.g., interpretations, inferences, and themes); might include development of a theory or model, or integration with prior research or theory	Findings section- data analysis uses the WHO health systems building blocks
S17 Links to empirical data	Evidence (e.g., quotes, field notes, text excerpts, photographs) to substantiate analytic findings	Verbatim quotes used in the Findings section

Discussion		
S18 Integration with prior work, implications, transferability, and contribution(s) to the field	Short summary of main findings; explanation of how findings and conclusions connect to, support, elaborate on, or challenge conclusions of earlier scholarship; discussion of scope of application/generalizability; identification of unique contribution(s) to scholarship in a discipline or field	Please refer to discussion section, pp 8
S19 Limitations	Trustworthiness and limitations of findings	Please refer to limitations section, page 9
**Other**		
S20 Conflicts of interest	Potential sources of influence or perceived influence on study conduct and conclusions; how these were managed	None declared
S21 Funding	Sources of funding and other support; role of funders in data collection, interpretation, and reporting	Role of funding source, page 4

[a]The rationale should briefly discuss the justification for choosing that theory, approach, method, or technique rather than other options available, the assumptions and limitations implicit in those choices, and how those choices influence study conclusions and transferability. As appropriate, the rationale for several items might be discussed together.

For peer review only
